# Rational design of a water-soluble, lipid-compatible fluorescent probe for Cu(i) with sub-part-per-trillion sensitivity†
†Electronic supplementary information (ESI) available: Synthetic procedures, compound characterization, and select spectroscopic data. See DOI: 10.1039/c5sc03643g


**DOI:** 10.1039/c5sc03643g

**Published:** 2015-12-01

**Authors:** M. T. Morgan, A. M. McCallum, C. J. Fahrni

**Affiliations:** a School of Chemistry and Biochemistry and Petit Institute for Bioengineering and Bioscience , Georgia Institute of Technology , 901 Atlantic Drive , Atlanta , GA 30332-0400 , USA . Email: fahrni@chemistry.gatech.edu ; Fax: +1 404 894 2294 ; Tel: +1 404 385 1164

## Abstract

Fluorescence probes represent an attractive solution for the detection of the biologically important Cu(i) cation; however, achieving a bright, high-contrast response has been a challenging goal. Concluding from previous studies on pyrazoline-based fluorescent Cu(i) probes, the maximum attainable fluorescence contrast and quantum yield were limited due to several non-radiative deactivation mechanisms, including ternary complex formation, excited state protonation, and colloidal aggregation in aqueous solution. Through knowledge-driven optimization of the ligand and fluorophore architectures, we overcame these limitations in the design of CTAP-3, a Cu(i)-selective fluorescent probe offering a 180-fold fluorescence enhancement, 41% quantum yield, and a limit of detection in the sub-part-per-trillion concentration range. In contrast to lipophilic Cu(i)-probes, CTAP-3 does not aggregate and interacts only weakly with lipid bilayers, thus maintaining a high contrast ratio even in the presence of liposomes.

## Abstract 2

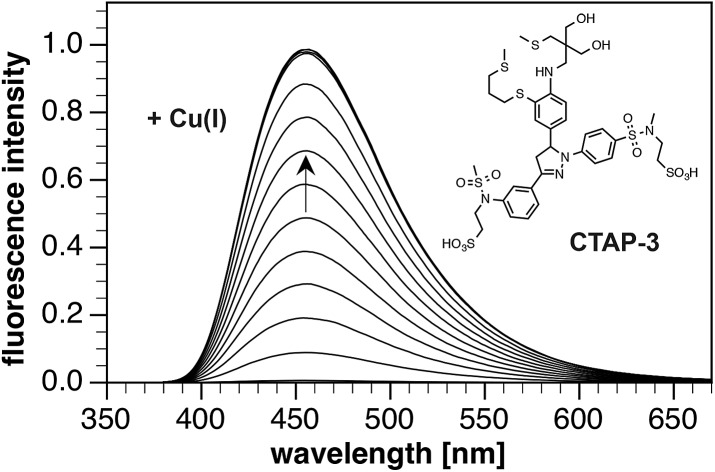
Knowledge-driven optimization of the ligand and fluorophore architectures yielded an ultrasensitive Cu(i)-selective fluorescent probe featuring a 180-fold fluorescence contrast and 41% quantum yield.

## Introduction

Synthetic fluorescent probes have become a mainstay of analytical chemistry for the low-cost detection of a wide array of metal cations.^1^–^8^ Due to the importance of Cu(i) in biology, the design of fluorescent probes for this cation has attracted considerable attention.^9^–^11^ Despite numerous efforts, the robust detection of Cu(i) remains challenging because of the inherent instability of free aquocopper(i) as well as the lipophilicity of thioether ligands typically employed for selective coordination.^12^ While a lipophilic architecture may be advantageous for cellular applications by allowing the probe to diffuse across lipid bilayers, such molecules are also prone to colloidal aggregation in aqueous solution, as we previously demonstrated for several Cu(i)-selective fluorescent probes.^13^ Because aggregation can dramatically alter the photophysical properties of the fluorophore, de-aggregation upon partitioning into a lipid phase may produce an analyte-independent fluorescence signal, thus rendering the interpretation of cellular staining patterns difficult. To avoid colloidal aggregation without compromising cell membrane permeability, we designed the water-soluble Cu(i)-probe CTAP-2 (**1**) composed of a hydroxylated thioether-arylamine ligand and a sulfonated triarylpyrazoline fluorophore.^13^ Operating on a photoinduced electron transfer (PET) fluorescence switching mechanism, CTAP-2 gave a 65-fold emission enhancement upon saturation with Cu(i), but the modest fluorescence quantum yield of 8% limited its detection sensitivity. Although sulfonation of the 1-aryl ring of CTAP-2 was sufficient to confer water solubility and to suppress aggregation in aqueous solution, we noticed that the probe was still able to participate in hydrophobic interactions with lipid membranes, presumably *via* the 3-aryl ring, thus resulting in a strong analyte-independent fluorescence response. This observation provided the impetus for the design of CTAP-3 (**2**) described in this report. This water-soluble, Cu(i)-selective fluorescent probe is not only compatible with lipid bilayers but also features much improved signaling characteristics, with an unprecedented 180-fold contrast ratio, a fluorescence quantum yield of 41%, and a limit of detection in the sub-part-per-trillion (ppt) concentration range.
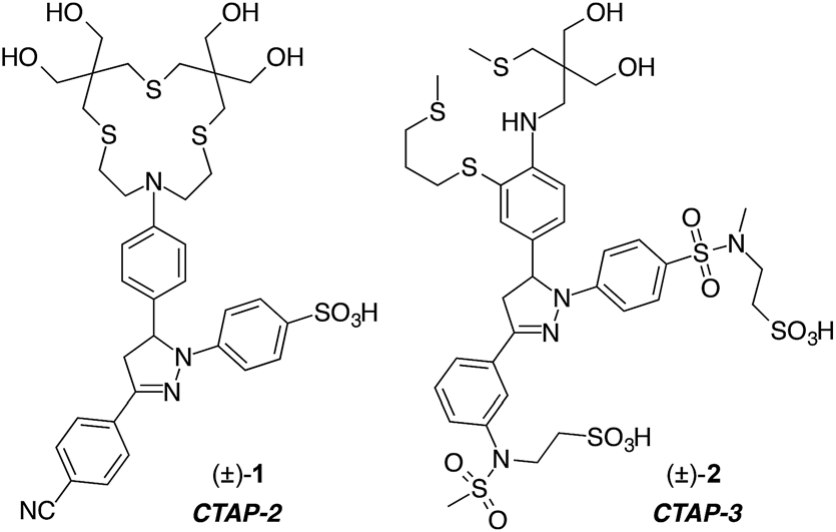


## Results and discussion

Detailed photophysical studies revealed that the fluorescence contrast and quantum yield of our second generation Cu(i)-selective fluorescent probe CTAP-2 are compromised by two independent quenching pathways. First, excited state protonation of the fluorophore by H_2_O competes with fluorescence emission,^14^ thus restricting the maximum attainable quantum yield. Second, significant residual PET quenching occurs in the Cu(i)-bound form due to partial dissociation of the Cu(i)–N bond, presumably driven by ternary complex formation with solvent molecules.^15^ As outlined in the following section, we used a knowledge-driven design approach to systematically address both of these challenging issues within a hydrophilic, lipid bilayer-compatible fluorophore platform.

### Suppression of excited state proton transfer

Previous studies on 1,3,5-triarylpyrazolines indicated that excited-state protonation can be suppressed by powerful electron withdrawing groups attached to the 1-aryl ring.^16^ We therefore replaced the moderately electron-withdrawing sulfonate group (Hammett parameter *σ*_p_ = 0.36)^17^ of CTAP-2 with a strongly electron-withdrawing *N*,*N*-dialkylsulfonamide (*σ*_p_ = 0.65) in which the anionic sulfonate moiety is tethered to the sulfonamide nitrogen through an ethylene spacer. We found that the 2-sulfoethyl group could be conveniently introduced by base-catalyzed conjugate addition of ethenesulfonyl fluoride to the corresponding secondary sulfonamide,^18^ and a similar methodology has recently been developed independently by Sharpless *et al.* for a wider array of functional groups.^19^ To further reduce lipophilicity in a synthetically convenient manner, we replaced the 3-aryl cyano-group (*σ*_p_ = 0.66) of CTAP-2 with a second sulfonamide branch. We first evaluated the effect of these modifications *via* model compound **3**, in which PET can be suppressed through protonation of the dimethylamino group at acidic pH. In 1 mM aqueous HCl, **3** gave a fluorescence quantum yield of 59%, more than twice that of the corresponding CTAP-2 fluorophore analog containing a PET-inactive *N*,*N*,*N*-trimethylanilinium substituent in the 5-position of the pyrazoline ring (28% in neutral H_2_O or 1 mM HCl).^14^ Furthermore, time-dependent fluorescence decay measurements of **3** in deuterated *vs.* non-deuterated 1 mM hydrochloric acid revealed a modest lifetime change from 3.99 to 3.35 ns (Fig. S2†), corresponding to a small solvent isotope effect of 1.2. In light of the significantly larger value of 1.7 observed for the CTAP-2 fluorophore analog,^14^ the improved quantum yield of **3** under acidic conditions can be largely ascribed to inhibition of excited-state protonation.

### Ligand design

Because the interaction strength between Cu(i) and the electron donor portion of the ligand controls how effectively PET quenching is suppressed, the ligand design plays a major role in optimizing the fluorescence contrast. According to previous studies with *N*-arylthiazacrown Cu(i)-complexes, steric interference between the aniline ring and ligand backbone is alleviated through displacement of the arylamine nitrogen donor from the Cu(i) center by a solvent molecule, resulting in residual PET quenching.^15^
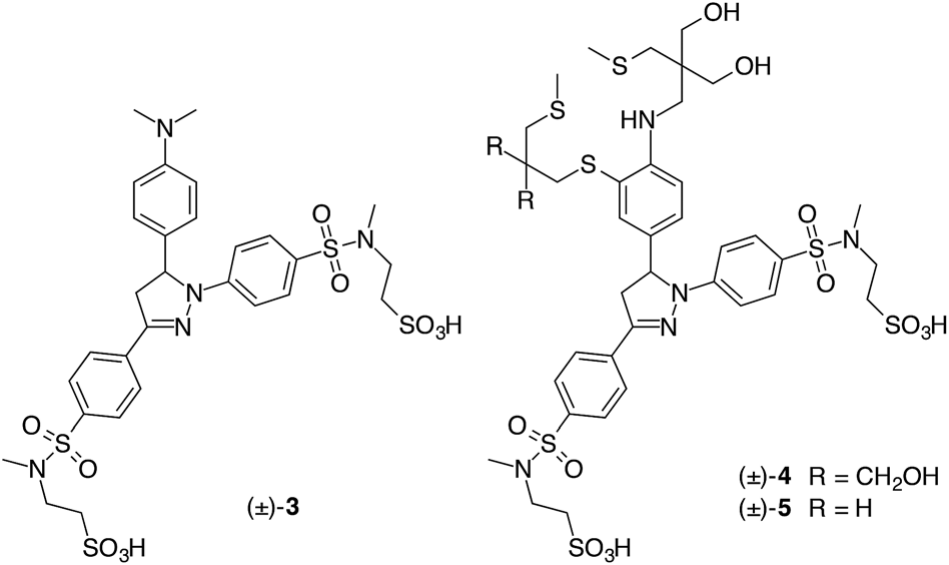


To minimize the internal strain upon Cu(i) coordination, we devised probe **4** in which the aniline moiety is integrated into the backbone of the arylamine–thioether framework. Based on our previous observation that the rigidity of a benzo-fused macrocyclic ligand may also hinder Cu(i) coordination,^14^ we switched to an acyclic topology for increased conformational flexibility. We previously demonstrated that this approach can generate excellent fluorescence turn-on responses for Cu(i)-probes in methanol,^20^ and it was later adopted by others in the design of Cu(i)-selective fluorescent probes.^21^,^22^ Compared to CTAP-2, probe **4** showed improvements in both contrast ratio (1 : 100) and fluorescence quantum yield (11%) upon saturation with Cu(i); however, the recovered quantum yield remained more than 5-times below that of reference compound **3** featuring the same fluorophore. Based on the crystal structure of the Cu(i) complex of a closely related acyclic ligand,^20^ we suspected that the hydroxymethyl substituents may still produce steric congestion upon Cu(i) coordination, thus weakening the Cu(i)–N interaction. We therefore eliminated one of the hydroxymethyl pairs to arrive at probe **5**, which showed further improvements in quantum yield (14%) and contrast ratio (1 : 160). While the latter is consistent with strong inhibition of PET upon Cu(i) coordination, the recovery of less than 25% of the intrinsic fluorophore quantum yield implies either substantial residual PET quenching or some other nonradiative deactivation pathway not present in reference compound **3**. Time-resolved fluorescence measurements of Cu(i)-saturated **5** offered further insights into the origin of this behavior (Fig. S3†). The fluorescence decay profile fit well to a biexponential model with components of 1.64 ns (21%) and 0.87 ns (79%), thus indicating the presence of at least two equilibrating coordination species with varying degrees of PET suppression.^15^

### Optimization of the PET driving force

As a large PET driving force may accentuate the difference in fluorescence lifetimes between species with only subtle variations in PET donor ability,^23^ we hypothesized that the limited fluorescence recovery of **5** could be due to an excessive PET driving force rather than weak Cu(i)–N coordination. If this is the case, then decreasing the driving force should improve both contrast ratio and quantum yield. While we have previously fine-tuned the PET accepting abilities of pyrazoline fluorophores *via* the 1-aryl substituents,^24^,^25^ in this case the strongly electron-withdrawing 1-aryl sulfonamide group serves to prevent fluorescence quenching by excited-state protonation,^14^,^16^ and we therefore chose to reduce the electron-withdrawing power of the 3-aryl ring instead. Given the Hammett substituent constants *σ*_p_ of 0.65 for –SO_2_NMe_2_*vs.* 0.24 for –N(Me)SO_2_Me,^17^ simple transposition of the sulfonamide group should substantially reduce its electron-withdrawing effect while preserving the solubilizing properties. Because a π-donor in conjugation with the pyrazoline imine nitrogen might increase its photobasicity, the transposed sulfonamide group was installed in the *meta*-position (*σ*_m_ = 0.21) of the 3-aryl ring to complete the optimization process for CTAP-3.

### Synthesis

**Scheme 1:**
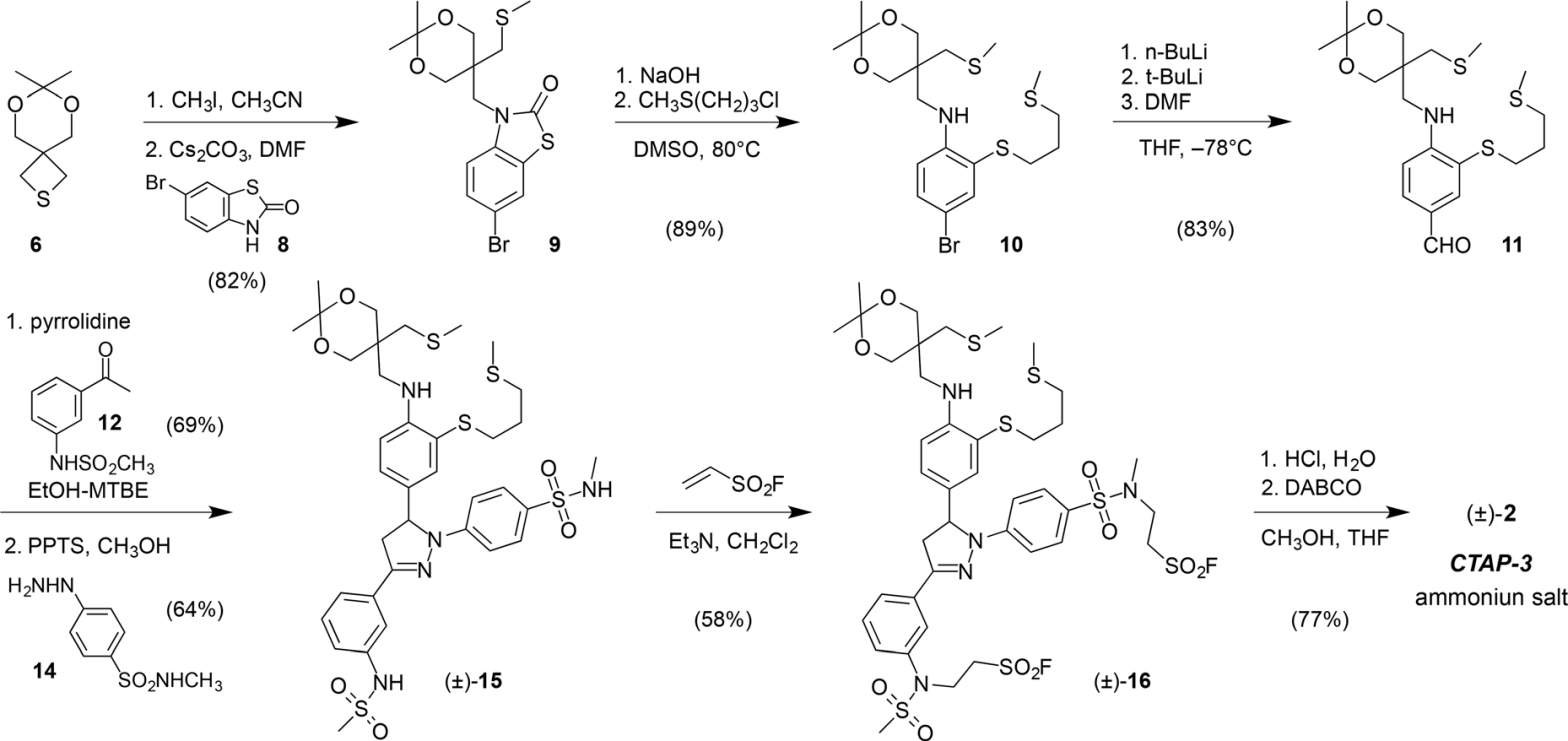
Synthesis of fluorescent probe **CTAP-3 (±)-2**.

CTAP-3 was assembled in 8 linear steps and 12% overall yield starting from the versatile thietane precursor **6** previously utilized for the synthesis of CTAP-2 (Scheme 1).^13^ Ring-opening with methyl iodide followed by nucleophilic substitution with 6-bromobenzothiazolinone **8** and a one-pot hydrolysis-*S*-alkylation yielded the ligand framework as bromide **10**, which could be efficiently converted to triarylpyrazoline **15** in three steps. Treatment with ethenesulfonyl fluoride in the presence of triethylamine resulted in selective alkylation of the acidic sulfonamide moieties over the arylamine NH, which represents an inverted chemoselectivity compared to that previously reported for ethenesulfonyl fluoride alone.^26^ In contrast to the isomeric sulfonyl fluoride precursor of probe **5**, which contains only *N*-alkylsulfonamides (see Scheme S4†), sulfonyl fluoride **16** could not be efficiently hydrolyzed to a bis-sulfonate with NaOH due to competing elimination of the more acidic *N*-arylsulfonamide unit. Instead, hydrolysis was effected under buffered conditions using 1,4-diazabicyclo[2,2,2]octane (DABCO) as a nucleophilic catalyst. HPLC purification using ammonium bicarbonate as a volatile background electrolyte^13^ delivered CTAP-3 as the water-soluble bis-ammonium salt after lyophilization.

### Photophysical characterization and solution chemistry

**Fig. 1:**
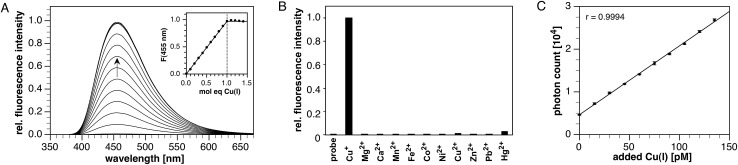
(A) Titration of CTAP-3 (4 μM) in pH 6 buffer (10 mM MES, 100 nM MCL-1) with 0.4 μM aliquots of Cu(i) generated *in situ* by reduction of Cu(ii)SO_4_ with 100 μM sodium ascorbate (*λ*_ex_ = 365 nm). (B) Fluorescence response of CTAP-3 (4 μM) in pH 7 buffer (10 mM PIPES, 100 nM MCL-1) to divalent cations (20 μM transition metals, 10 mM Ca(ii) and Mg(ii)) and to Cu(i) generated by *in situ* reduction of 5 μM Cu(ii) with 100 μM sodium ascorbate. The probe responded normally to Cu(i) in the presence of all competing ions (not shown). Excitation at 365 nm. (C) Titration of CTAP-3 (2 nM) in ascorbate buffer (200 μM ascorbic acid, 170 μM KOH, 100 pM MCL-3) with 15 pM (0.95 part per trillion) aliquots of Cu(ii)SO_4_ reduced *in situ* to Cu(i). Excitation at 355 nm (10 nm monochromator bandpass) with 370/36 nm sharp cutoff bandpass filter. Emission collected at 455 nm (10 nm bandpass). Error bars represent the standard deviation over 6 successive 5 second measurements for each titration point.

Stock solutions of CTAP-3 at millimolar concentrations can be prepared directly in water without the need for an organic co-solvent. Diluted to micromolar concentrations in neutral aqueous buffer, CTAP-3 gives a large 180-fold fluorescence increase upon saturation with Cu(i) to reach a maximum quantum yield of 41% (Fig. 1A). Despite the use of 18.2 MΩ•cm Milli-Q water for buffer preparation, we observed an increased background fluorescence due to nanomolar levels of adventitious copper, and accurate determination of the enhancement factor thus required supplementation of the buffer with the high affinity chelator MCL-1 (100 nM)^27^ as a sequestrant. Consistent with the formation of a complex with 1 : 1 stoichiometry, the fluorescence increase plateaus at 1 molar equivalent of Cu(i) (Fig. 1A, inset). As the free probe is essentially non-fluorescent, the emission maximum at 455 nm remains unchanged throughout the molar ratio titration. The absorption maximum shifts slightly from 367 to 364 nm upon Cu(i) addition (Fig. S1†), presumably due to a small electrostatic field effect. The time-resolved fluorescence decay profile for the Cu(i)-saturated probe fit well to a monoexponential model with a lifetime of 2.48 ns (*χ*^2^ = 1.59, Fig. S3†), indicating effective suppression of PET upon Cu(i) coordination. As illustrated in Fig. 1B, CTAP-3 exhibits excellent selectivity towards Cu(i) and does not respond to any of the biologically relevant divalent transition metal cations. In the presence of Cu(ii), the probe undergoes a very slow and irreversible reaction to produce 4% of the maximum fluorescence intensity of the Cu(i)-saturated form after 30 min; however, no significant interference was observed at short time scales. The fluorescence emission of CTAP-3 remains quenched over a large pH range (Fig. S4†) and increases only below pH 4 upon protonation of the aniline moiety.

Acidification below pH 1.5 again results in fluorescence quenching, indicating the presence of a second protonation equilibrium that must be associated with the fluorophore moiety. Non-linear least squares fitting of the fluorescence response over the entire pH range yielded p*K*_a_'s of 2.00 ± 0.01 and 1.32 ± 0.01 for the two protonation equilibria. Competition titration of CTAP-3 with the previously reported Cu(i) affinity standard MCL-2 (ref. 27) at pH 6.0 (10 mM MES buffer, 0.1 M KClO_4_) revealed a Cu(i) complex stability constant of log *K* = 10.29 ± 0.06 (Fig. S5†). Given the low p*K*_a_ of 2.0 for the aniline nitrogen, the apparent Cu(i) affinity of CTAP-3 is expected to remain constant above pH 4.

### Limit of detection

The high contrast and fluorescence quantum yield offered by CTAP-3 should, in principle, permit the detection of Cu(i) with unprecedented sensitivity. Furthermore, the substantial Stokes shift of 5300 cm^–1^, corresponding to an 88 nm separation between the absorption and emission maxima, allows the emission intensity of CTAP-3 to be recorded without interference from Raman scattering, even at low nanomolar probe concentrations. Using a conventional bench-top fluorimeter equipped with a 75 W xenon lamp excitation source and 10 nm bandpass for the excitation and emission monochromators, we titrated a 2 nM solution of CTAP-3 with 15 pM aliquots of CuSO_4_ in the presence of excess ascorbate as reductant. At each titration step, the fluorescence emission was collected at 465 nm with excitation at 355 nm and an integration time of 5 seconds (Fig. 1C), yielding a linear intensity response, which was fully reversible upon addition of the higher-affinity pH-independent Cu(i)-chelator MCL-3 (ref. 27) (log *K* = 13.8). Because initial experiments indicated a background contamination with copper around 100 pM, despite the use of HPLC-grade water and high-purity ascorbic acid, the data were acquired in the presence of 100 pM MCL-3 (ref. 27) as a sequestrant. Remarkably, the difference in fluorescence intensity between aliquots was always at least 6 times the standard deviation within each aliquot reading, implying that the actual limit of detection, defined as the concentration of analyte yielding at least a 3 : 1 signal-to-noise ratio, is less than 7.5 pM or 0.5 parts-per-trillion (ppt) of Cu(i).

### Lipid compatibility

**Fig. 2:**
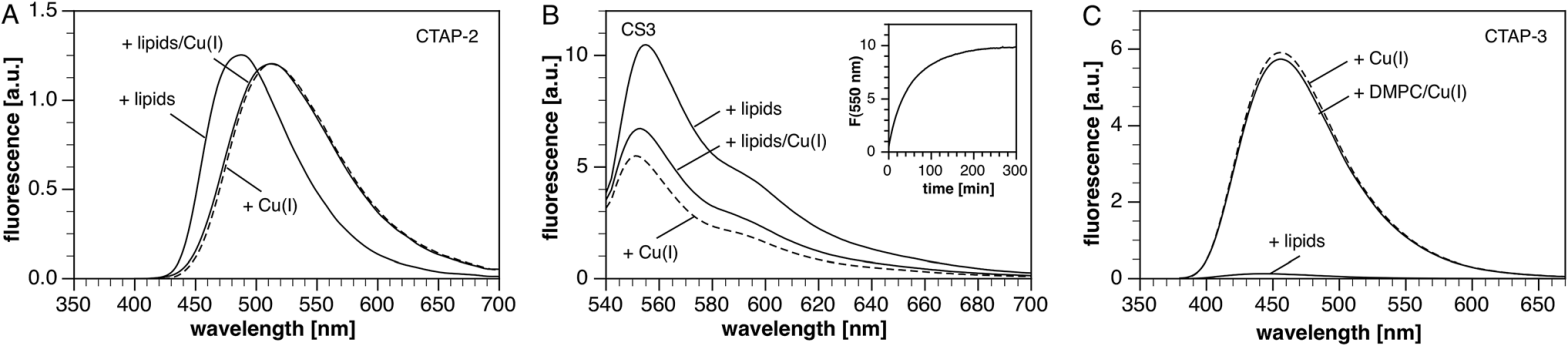
(A) Fluorescence response of CTAP-2 (2 μM, *λ*_ex_ 380 nm) upon saturation with Cu(i) in the presence of DMPC/DMPG liposomes (4 : 1 mixture, 100 μM) in pH 7.0 buffer at 25 °C (10 mM PIPES, 0.1 M KClO_4_, 100 nM MCL-1). (B) Fluorescence response of coppersensor-3 (CS3, 2 μM) under the same set of conditions (*λ*_ex_ 530 nm). Inset: time-dependent evolution of the fluorescence intensity at 550 nm prior to addition of Cu(i). (C) Fluorescence response of CTAP-3 (2 μM) under the same set of condition used in (A) for CTAP-2 (*λ*_ex_ 365 nm).

Fluorescent probes that exist as colloidal aggregates in aqueous solution are likely to show very different signaling behavior in the presence of a lipid bilayer. Even in the absence of an aggregation equilibrium, association with a lipid bilayer can affect the fluorescence characteristics due to the lower environmental polarity. To gauge the importance of this effect for CTAP-3 in comparison to previous Cu(i) probes, we evaluated the signaling response in the presence of liposomes as model membranes. Given the variable mixture of neutral and anionic lipid head groups of biological membranes, we prepared liposomes composed of a 4 : 1 ratio of zwitterionic dimyristoyl phosphatidylcholine (DMPC) and anionic dimyristoyl phosphatidylglycerol (DMPG). Both lipids are stable towards peroxidation and hydrolysis during storage, and exhibit similar phase transition temperatures.^28^ Despite its high water-solubility and non-aggregating properties, we noted that CTAP-2 still produced a strong liposome-mediated turn-on response, reaching roughly the same fluorescence intensity as that of the Cu(i)-bound form in the absence of liposomes (Fig. 2A). The emission maximum is substantially blue-shifted, as expected for a triarylpyrazoline fluorophore in a lower polarity environment.^29^,^30^ Upon addition of Cu(i) to the liposome–CTAP-2 solution, the fluorescence spectrum shifts to match that observed in the absence of liposomes, suggesting that the Cu(i)-bound form does not significantly associate with the lipid bilayers. To test the effect of lipid bilayers on a colloidal Cu(i)-selective probe,^13^ we measured the fluorescence response of the widely employed^31^–^35^ BODIPY-based coppersensor-3 (CS3)^36^ under the same conditions. Consistent with slow dissolution of a colloidal aggregate into the lipid bilayer,^37^ CS3 responded with a strong, time-dependent fluorescence increase (Fig. 2B, inset) when added to aqueous buffer containing DMPC–DMPG liposomes. The rate of the response varies with stirring rate and requires several hours to reach completion. Upon saturation with Cu(i), the fluorescence maximum is slightly shifted towards shorter wavelength and the fluorescence intensity drops by approximately 40% (Fig. 2B). Surprisingly, the liposome-mediated turn-on response in the absence of Cu(i) is approximately 50% stronger than the Cu(i)-mediated response in the absence of liposomes. In contrast to CTAP-2 and CS3, the background fluorescence of CTAP-3 is only slightly elevated upon addition of liposomes, and the probe still offers a greater than 60-fold fluorescence enhancement upon saturation with Cu(i) (Fig. 2C). As observed for CTAP-2, the fluorescence emission spectrum of the Cu(i)-saturated probe remained unaltered in the presence of liposomes compared to neat buffer. Interestingly, a control experiment with CTAP-3 in neutral DMPC liposomes also yielded a 60-fold fluorescence enhancement upon addition of Cu(i), demonstrating that the weakness of the interaction between CTAP-3 and liposomes cannot be attributed simply to electrostatic repulsion of the dianionic probe.

## Conclusions

In summary, systematic optimization of the ligand and fluorophore framework yielded a water-soluble Cu(i)-selective fluorescent probe with unprecedented detection sensitivity. The probe directly dissolves in water and offers a strong turn-on response even in the presence of liposomes. This lipid compatibility, which has not been demonstrated for any other Cu(i)-selective fluorescence turn-on probe, opens the possibility of interrogating protein-mediated Cu(i) transport across lipid bilayers. Because lipophilic turn-on probes such as CS3 change their fluorescence properties in the presence of membranes, the interpretation of their fluorescence response within the complex chemical environment of biological systems should be approached with caution.^38^ Despite the many challenges surrounding the detection of the redox-labile Cu(i) cation, this work demonstrates that the design of high-contrast probes can be achieved after carefully optimization of the probe properties.

## Supplementary Material

Supplementary information
