# Supplementary material for: Rational design of a water-soluble, lipid-compatible fluorescent probe for Cu(i) with sub-part-per-trillion sensitivity
Source: Chem Sci. 2015 Dec 1;7(2):1468–73. doi: 10.1039/c5sc03643g (PMC5201193; doi:10.1039/c5sc03643g)
Supplement: Supplementary file 1 [file SC-007-C5SC03643G-s001.pdf]

Electronic Supplementary Material (ESI) for Chemical Science.
This journal is © The Royal Society of Chemistry 2015

Supplementary Information for

Rational Design of a Water-Soluble, Lipid-Compatible Fluorescent Probe for Cu(I) with Sub-Part-Per-Trillion Sensitivity

M. Thomas Morgan, Adam M. McCallum and Christoph J. Fahrni*

School of Chemistry and Biochemistry and Petit Institute for Bioengineering and Bioscience, Georgia Institute of Technology, 901 Atlantic Drive, Atlanta, Georgia 30332, USA

Table of Contents

1. Synthetic procedures S2
   ^{1}H-, ^{13}C-, and ^{19}F-NMR spectra S14

2. Absorption and Fluorescence Spectroscopy S38
   Fluorescence Enhancement Factors and Quantum Yields S38
   Time-resolved Fluorescence Spectroscopy S39
   Analyte Selectivity (CTAP-3) S40
   Molar-ratio Titration of CTAP-3 with Cu(I) S40
   Determination of the Detection Limit S40
   Determination of the Protonation Constant of CTAP-3 S41
   Cu(I) Binding Affinity of CTAP-3 S41
   Preparation of Liposomes S43

3. References S44

1. Synthetic Procedures

Materials and Reagents. Ethenesulfonyl fluoride (ESF),[1] thietane **6**,[2] 6-bromobenzothiazolin-2-one,[3] and benzothiazolin-2-one-6-carboxaldehyde[4] **21** were prepared as previously described. All other starting materials were commercially available and used without further purification. NMR: Spectra were recorded at 400 MHz (^{1}H, ppm vs. internal TMS, referenced directly or indirectly via the known residual proton signal of the solvent), 376 MHz (^{19}F, ppm vs. internal CCl_3F), and 100 MHz (^{13}C, ppm vs. TMS, referenced to $CDCl_3$ (77 ppm) or CD_3OD (49 ppm) chemical shifts). Spectra were recorded at ambient temperature (20-23°C) unless stated otherwise. For ^{1}H spectra, the abbreviation "ad" denotes an apparent doublet with additional partially resolved coupling (AA'XX' or AA'MM' spin system); only the largest (first order) coupling constant is given for these systems. In cases where the product as isolated contained a substantial amount of solvent, the solvent content was calculated from the initial ^{1}H NMR integrals and a second ^{1}H NMR spectrum was acquired after removal of solvent by repeated dissolution in $CDCl_3$ followed by concentration to dryness. MS: Spectra were acquired by the Georgia Tech Mass Spectrometry Facility. Column chromatography: Flash chromatography on Sorbent Technologies, general purpose silica gel (60 Å pore size, 250 mesh).

1.1 Synthesis of Pyrazoline Probe (±)-2 (CTAP-3)

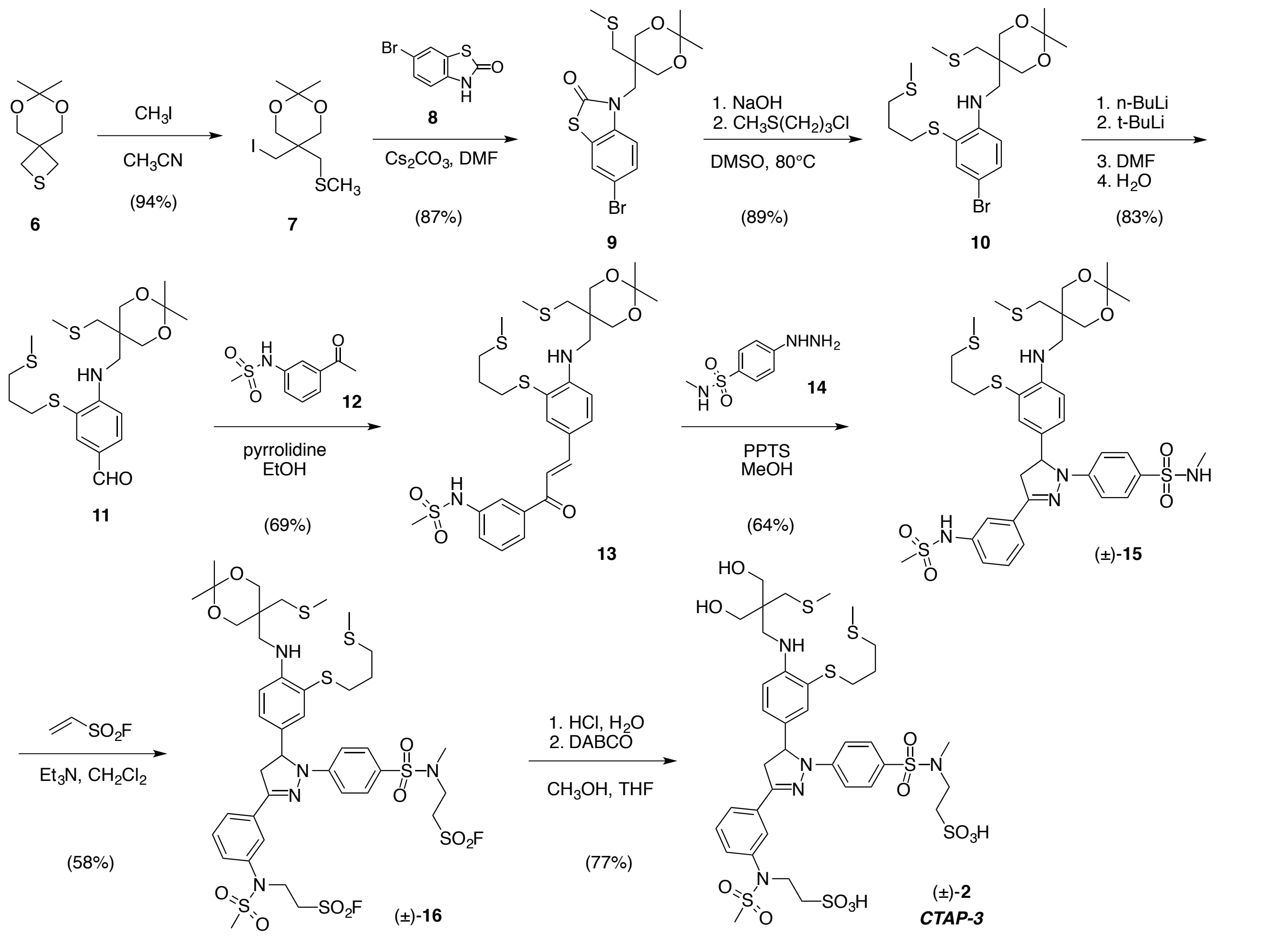

Scheme S1: Overview for the synthesis of pyrazoline probe (±)-**2** (CTAP-3)

5-(Iodomethyl)-2,2-dimethyl-5-((methylthio)methyl)-1,3-dioxane (**7**). A mixture of thietane **6** (10.1 g, 57.8 mmol),[2] methyl iodide (4.3 mL, 1.2 equiv.), powdered K_2CO_3 (200 mg), and acetonitrile (15 mL) was stirred under argon at 60°C for 20 hours. The mixture was concentrated under reduced pressure, and the oily residue was taken up in dichloromethane (50 mL), stirred with silica gel (3 g), and filtered through a pad of silica gel. The silica gel was washed with a further 50 mL of dichloromethane, and the combined filtrate and washing were concentrated under reduced pressure to give the product **7** as a colorless oil. Yield 17.2 g (54.5 mmol, 94%). ^{1}H NMR ($CDCl_3$) δ 1.41 (s, 3H), 1.42 (s, 3H), 2.19 (s, 3H), 2.69 (s, 2H), 3.42 (s, 2H), 3.73 (d, J = 11.8 Hz, 2H), 3.80 (d, J = 11.8 Hz, 2H). Note: in some samples, a partly resolved long-range coupling (< 0.5 Hz) is observed for the methyl signals at 1.41 and 1.42 ppm. ^{13}C NMR ($CDCl_3$) δ 12.9, 17.9, 23.1, 24.0, 37.1, 39.2, 66.3, 98.6. EI-MS *m/z* 316 ([M]$^+$, 65), 301 (67), 131 (82), 101 (55), 83 (82), 61 (100), 55 (62). EI-HRMS *m/z* calcd for $C_9H_{17}IO_2S$ 315.9994, found 315.9999.

Benzothiazolinone **9.** A mixture of 6-bromobenzothiazolin-2-one[3] (**8**, 4.37 g, 19.0 mmol), iodide **7** (6.61 g, 1.1 equiv.), Cs_2CO_3 (9.3g, 1.5 equiv.), and DMF (10 mL) was stirred at 90°C under argon overnight. The mixture was diluted with water (100 mL) and extracted with MTBE (100 mL). The extract was washed with 5% aqueous NaOH (100 mL) followed by water (100 mL) + brine (10 mL), dried with $MgSO_4$, filtered, and concentrated to give a yellow oil that solidified on contact with methanol. The resulting material was recrystallized from methanol under stirring to give a colorless crystalline powder. Yield 6.90 g (87%). ^{1}H NMR ($CDCl_3$) δ 1.47 (s, 6H), 2.13 (s, 3H), 2.66 (s, 2H), 3.62 (d, J = 12.5 Hz, 2H), 4.00 (d, J = 12.5 Hz, 2H), 4.14 (s, 2H), 7.44 (dd, J = 8.7, 1.7 Hz, 1H), 7.47 (dd, J = 8.7, 0.6 Hz, 1H), 7.54 (dd, J = 1.7, 0.6 Hz, 1H). ^{13}C NMR ($CDCl_3$) δ 18.4, 21.4, 26.3, 38.1, 40.2, 46.4, 64.6, 98.5, 112.9, 115.8, 124.1, 124.8, 129.6, 137.4, 170.7. EI-MS *m/z* 419 (27) 417 ([M]$^+$, 25), 404 (35), 402 (32), 361 (35), 359 (32), 328 (50), 326 (45), 135 (52), 97 (100), 82 (80), 61 (70). EI-HRMS *m/z* calcd for [M]$^+$ $C_{16}H_{20}NO_3S_2{}^{79}Br$ 417.0068, found 417.0050.

Bromide **10.** Benzothiazolinone **9** (4.80 g, 11.5 mmol) was dissolved in DMSO (40 mL) under argon at 80°C. Aqueous NaOH (5 M, 8.3 mL, 3.6 equiv.) was injected as a slow stream into the rapidly stirred solution. After 30 minutes, the reaction was complete by TLC (5:1 hexane-EtOAc). The mixture was cooled to 60°C and acetic acid (0.85 mL, 1 equiv) was added, followed by 1-chloro-3-methylthiopropane (1.43 g, 1 equiv.) in DMSO (2 mL). After 30 minutes, a trace of the intermediate thiophenol remained distinguishable by TLC, so a further 0.1 equiv. (143 mg) of 1-chloro-3-methylthiopropane was added. After 15 minutes, TLC indicated complete consumption of the thiophenol. The mixture was partitioned between water (300 mL) and MTBE (140 mL), and the organic layer was washed twice with water (200 mL) + brine (10 mL), dried with $MgSO_4$, and concentrated. The residue was separated by column chromatography (hexanes-MTBE) to give product **10** as a colorless oil. Yield 4.93 g (10.3 mmol, 89%). ^{1}H NMR ($CDCl_3$) δ 1.45 (s, 3H), 1.46 (s, 3H), 1.83 (p, J = 7.1 Hz, 2H), 2.06 (s, 3H), 2.16 (s, 3H), 2.58 (t, J = 7.0 Hz, 2H), 2.60 (s, 2H), 2.81 (t, J = 7.1 Hz, 2H), 3.38 (d, J = 6.6 Hz, 2H), 3.71 (d, J = 12.0 Hz, 2H), 3.78 (d, J = 12.0 Hz, 2H), 5.32 (t, J = 6.5 Hz, 1H), 6.71 (d, J = 8.8 Hz, 1H), 7.28 (dd, J = 8.8, 2.4 Hz, 1H), 7.48 (d, J = 2.4 Hz, 1H). ^{13}C NMR ($CDCl_3$) δ 15.4, 17.8, 21.8, 25.6, 28.5, 32.8, 33.6, 38.3, 38.7, 45.9, 65.4, 98.5, 107.6, 111.7, 119.1, 132.8, 137.6, 148.7. EI-MS *m/z* 481 (70), 479 ([M]$^+$, 65), 306 (43), 304 (39), 216 (40), 214 (35), 89 (100), 61 (63). EI-HRMS *m/z* calcd for [M]$^+$ $C_{19}H_{30}NO_2S_3{}^{79}Br$ 479.0622, found 479.0610.

Aldehyde **11.** An oven-dried 50 mL round-bottom flask was charged with bromide **10** (642 mg, 1.34 mmol), sealed with a rubber septum, and flushed with argon. Anhydrous THF (14 mL) was

added, and the solution was cooled in a dry ice-acetone bath. After 15 min, *n*-butyllithium (2.5 M in hexanes, 1.1 mL, 2 equiv.) was added dropwise to the stirred solution. After 5 min, *t*-butyllithium (1.6 M in pentane, 2.5 mL, 3 equiv.) was added dropwise. After 30 min, anhydrous DMF (1.0 mL, 10 equiv.) was added, the cooling bath was removed, and the mixture was quenched with water once the temperature rose to ~0°C. The mixture was partitioned between water (100 mL) and MTBE (100 mL), and the organic layer was washed with water + brine (100 mL + 10 mL), dried with $MgSO_4$, and concentrated. The residue was separated by column chromatography (hexanes-MTBE) to give product **11** as a pale yellow oil. Yield 458 mg (83%). ^{1}H NMR ($CDCl_3$) δ 1.46 (s, 3H), 1.49 (s, 3H), 1.84 (p, *J* = 7.1 Hz, 2H), 2.05 (s, 3H), 2.17 (s, 3H), 2.57 (s, 2H), 2.59 (t, *J* = 7.0 Hz, 2H), 2.83 (t, *J* = 7.1 Hz, 2H), 3.56 (d, *J* = 6.5 Hz, 2H), 3.70 (d, *J* = 12.2 Hz, 2H), 3.82 (d, *J* = 12.2 Hz, 2H), 6.16 (t, *J* = 6.4 Hz, 1H), 6.91 (d, *J* = 8.6 Hz, 1H), 7.72 (dd, *J* = 8.6, 2.0 Hz, 1H), 7.92 (d, *J* = 2.0 Hz, 1H), 9.70 (s, 1H). ^{13}C NMR ($CDCl_3$) δ 15.3, 17.8, 21.1, 26.3, 28.5, 32.7, 33.7, 38.2, 38.7, 45.5, 65.4, 98.7, 109.2, 117.5, 126.1, 133.1, 138.2, 154.2, 189.6. EI-MS *m/z* 429 ($[M]^+$, 85), 254 (38), 164 (34), 89 (100), 61 (40). EI-HRMS calcd for $C_{20}H_{31}NO_3S_3$ 429.1466, found 429.1475.

N-(3-Acetylphenyl)methanesulfonamide **12**.[5] *m*-Aminoacetophenone (9.09 g, 67.3 mmol) was dissolved in pyridine (27 mL, 5 equiv.), and the solution was cooled in an ice bath under a slow stream of argon. Methanesulfonyl chloride (6.8 mL, 1.3 equiv.) was added dropwise to the stirred solution. After 5 min, the reaction was quenched by adding crushed ice and poured over a slurry of crushed ice (~100 g) and concentrated HCl (25 mL). The resulting red emulsion was extracted with dichloromethane (100 mL) + MTBE (200 mL). The yellow organic layer (top), dried with $MgSO_4$, filtered, and concentrated. The residue was recrystallized from cyclohexane-ethyl acetate to give the product **12** as a slightly yellowish crystalline powder. Yield 10.41 g (48.8 mmol, 73%) A further 1.26 g of pure product was recovered from the emulsion layer remaining after extraction, bringing the total yield to 11.67 g (81%). ^{1}H NMR ($CDCl_3$) δ 2.63 (s, 3H), 3.06 (s, 3H), 7.38 (br. s, 1H), 7.48 (t, *J* = 7.9 Hz 1H), 7.56 (ddd, *J* = 8.1, 2.3, 1.1 Hz, 1H), 7.77 (dt, *J* = 7.7, 1.2 Hz, 1H), 7.84 (t, *J* = 1.8 Hz, 1H). ^{13}C NMR ($CDCl_3$) δ 26.7, 39.6, 120.0, 125.1, 125.2, 130.0, 137.5, 138.4, 197.7.

Chalcone **13.** A solution of aldehyde **11** (975 mg, 2.27 mmol), *N*-(3-acetylphenyl)methanesulfonamide **12** (484 mg, 1 equiv.) and pyrrolidine (95 μL, 0.5 equiv.) in ethanol (6 mL) was stirred for 25 hours at 45°C, producing a red, oily biphasic mixture. A small aliquot (~50 μL) of the lower phase was removed, concentrated, and separated by column chromatography in a Pasteur pipette (2:2:1 hexanes-dichloromethane-MTBE). The presumed chalcone product (bright orange band) was crystallized from MTBE-hexane. The bulk reaction mixture was diluted with 6 mL MTBE, stirred until homogeneous, and seeded with the crystalline material. The resulting crystalline slurry was stirred for 4 days at 30°C, diluted into toluene (100 mL) and washed with a mixture of sat. aqueous Na_2CO_3 (15 mL), sat. aqueous $NaHCO_3$ (15 mL) and water (70 mL) followed by 1 M NaH_2PO_4 (25 mL). The organic layer was dried with Na_2SO_4 and concentrated, and the residue was separated by column chromatography (2:2:1 hexanes-dichloromethane-MTBE). The resulting orange, glassy, slightly impure product was crystallized from MTBE to give chocolate-colored leaflets that turned yellow-orange after drying under vacuum. Yield 981 mg (1.57 mmol, 69%). ^{1}H NMR ($CDCl_3$) δ 1.47 (s, 3H), 1.49 (s, 3H), 1.86 (p, *J* = 7.1 Hz, 2H), 2.06 (s, 3H), 2.18 (s, 3H), 2.60 (s, 2H), 2.61 (t, *J* = 7.0 Hz, 2H), 2.85 (t, *J* = 7.2 Hz, 2H), 3.06 (s, 3H), 3.53 (d, *J* = 6.5 Hz, 2H), 3.72 (d, *J* = 12.1 Hz, 2H), 3.82 (d, *J* = 12.1 Hz, 2H), 5.92 (t, *J* = 6.5 Hz, 1H), 6.88 (d, *J* = 8.7 Hz, 1H), 7.37 (d, *J* = 15.4 Hz, 1H), 7.51 (t, *J* = 7.9 Hz, 1H), 7.61 (dd, *J* = 8.7, 2.0 Hz, 1H), 7.72 (ddd, *J* = 8.1, 2.3, 0.9 Hz, 1H), 7.81 (d, *J* = 2.0 Hz, 1H), 7.83-7.85 (m, 1H), 7.97 (d, *J* = 15.4 Hz, 1H), 8.10 (br. s, 1H), 8.14 (br. t, *J* = 1.9 Hz, 1H).

^{13}C NMR ($CDCl_3$) δ 15.4, 17.8, 21.4, 26.1, 28.6, 32.8, 33.8, 38.3, 38.8, 39.5, 45.5, 65.5, 98.7, 110.0, 116.0, 117.7, 120.6, 123.5, 124.1, 124.8, 129.8, 132.1, 137.3, 138.2, 139.9, 146.8, 151.9, 189.7. EI-MS *m/z* 624 ($[M]^+$, 45), 198 (10), 121 (12), 89 (100), 73 (45), 61 (37). EI-HRMS m/z calcd for $[M]^+$ $C_{29}H_{40}N_2O_5S_4$ 624.1820, found 624.1833.

Triarylpyrazoline (±)-**15**. A mixture of chalcone **13** (343 mg, 549 μmol) and PPTS (276 mg, 2 equiv.) in methanol (5 mL) was boiled for 15 min, then concentrated to an oily residue under a stream of argon. Arylhydrazine **14** (144 mg, 1.3 equiv.) and methanol (3 mL) were added, and the mixture was stirred under argon in a sealed vessel at 90°C for 2 hours. The mixture was concentrated to dryness, and the residue was taken up in acetone (5 mL) + 2,2-dimethoxypropane (3 mL). The mixture was boiled for 15 min, allowed to cool, and partitioned between water (100 mL) and dichloromethane (50 mL). The organic layer was collected, and the aqueous layer was extracted with dichloromethane (20 mL). The combined organic layers were dried with Na_2SO_4 and concentrated, and the residue was separated by column chromatography (dichloromethane-MTBE) to give the product as a pale yellow glassy solid. Yield 282 mg (349 μmol, 64%). ^1H NMR ($CDCl_3$) δ 1.440 (s, 3H), 1.444 (s, 3H), 1.69 (p, *J* = 7.1 Hz, 2H), 2.00 (s, 3H), 2.14 (s, 3H), 2.44-2.55 (m, 2H), 2.59 (s, 2H), 2.60 (d, *J* = 5.4 Hz, 3H), 2.73 (t, *J* = 7.1 Hz, 2H), 3.05 (s, 3H), 3.15 (dd, *J* = 17.3, 5.9 Hz, 1H), 3.34-3.43 (m, 2H), 3.70 (d, *J* = 12.0 Hz, 2H), 3.79 (d, *J* ≈ 12 Hz, overlaps with subsequent signal, 2H), 3.81 (dd, *J* = 17.3, 12.2 Hz, 1H), 4.45 (q, *J* = 5.5 Hz, 1H), 5.23 (dd, *J* = 12.2, 6.0 Hz, 1H), 5.37 (t, *J* = 6.6 Hz, 1H), 6.79 (d, *J* = 8.6 Hz, 1H), 7.06 (br. s, 1H), 7.07 (dd, *J* = 8.5, 2.2 Hz, 1H), 7.11 (ad, *J* = 9.0 Hz, 2H), 7.24 (d, *J* = 2.2 Hz, 1H), 7.29 (ddd, *J* = 8.0, 2.2, 1.0 Hz, 1H), 7.38 (t, *J* = 7.9 Hz, 1H), dt (*J* = 7.9, 1.2 Hz, 1H), 7.61-7.74 (m, 3H). ^{13}C NMR ($CDCl_3$) δ 15.3, 17.8, 21.7, 25.8, 28.4, 29.4, 32.6, 33.4, 38.3, 38.7, 39.5, 43.5, 45.9, 63.1, 65.4, 65.5, 98.6, 110.8, 112.8, 117.8, 118.0, 121.2, 122.9, 127.0, 127.7, 128.7, 128.9, 130.0, 133.3, 133.7, 137.3, 147.1, 148.5, 149.4. ESI-MS *m/z* 808 ($[M+H]^+$, 100), 663 (12). ESI-HRMS m/z calcd for $[M+H]^+$ $C_{36}H_{50}N_5O_6S_5$ 808.2365, found 808.2364.

Bis(sulfonyl fluoride) derivative (±)-**16.** A solution of triarylpyrazoline (±)-**15** (169 mg, 209 μmol), triethylamine (87 μL, 3 equiv.), and ethenesulfonyl fluoride (105 μL, 6 equiv.) in dry dichloromethane (2 mL) was stirred under argon for 3 hours, then diluted with toluene (2 mL) and concentrated to dryness. The residue was separated by column chromatography (1:1 dichloromethane-hexanes plus increasing MTBE) and the resulting glassy product was crystallized from dichloromethane-MTBE to give an ivory-colored crystalline powder. Yield 125 mg (122 μmol, 58%). ^1H NMR ($CDCl_3$) δ 1.44 (br. s, 6H), 1.72 (p, *J* = 7.1 Hz, 2H), 2.01 (s, 3H), 2.15 (s, 3H), 2.46-2.57 (m, 2H), 2.59 (s, 2H), 2.75 (t, *J* = 7.1 Hz, 2H), 2.81 (s, 3H), 3.01 (s, 3H), 3.19 (dd, *J* = 17.4, 6.0 Hz 1H), 3.40 (d, *J* = 6.6 Hz, 2H), 3.48-3.52 (m, 2H), 3.70 (d, *J* = 11.8 Hz, 2H), 3.71-3.76 (m, 4H), 3.80 (d, *J* = 11.8 Hz, 2H), 3.86 (dd, *J* = 17.4, 12.2 Hz, 1H), 4.28 (br. t, *J* ≈ 7 Hz, 2H), 5.27 (dd, *J* = 12.2, 6.0 Hz, 1H), 5.41(t, *J* = 6.6 Hz, 1H), 6.81 (d, *J* = 8.6 Hz, 1H) 7.09 (dd, *J* = 8.5, 2.2 Hz, 1H), 7.16 (ad, *J* = 9.0 Hz, 2H), 7.27 (d, *J* = 2.2 Hz, 1H), 7.37 (ddd, *J* = 8.0, 2.1, 1.0 Hz, 1H), 7.51 (t, *J* = 7.9 Hz, 1H), 7.60 (ad, *J* = 9.1 Hz, 2H), 7.72 (dt, *J* ≈ 8.0, 1.2 Hz, 1H), 7.76 (t, *J* = 1.8 Hz, 1H). ^{19}F NMR ($CDCl_3$) δ 56.3 (t, *J* = 4.9 Hz, 1F), 57.3 (t, *J* = 4.3 Hz, 1F). ESI-MS *m/z* 1028 ($[M+H]^+$, 100), 663 (14). ESI-HRMS m/z calcd for $[M+H]^+$ $C_{40}H_{56}F_2N_5O_{10}S_7$ 1028.2040, found 1028.2040.

Pyrazoline probe (±)-**2** (CTAP-3). Bis(sulfonyl fluoride) derivative (±)-**16** (60 mg, 58 μmol) was stirred in a mixture of methanol (1 mL), THF (0.5 mL), and 1 M aqueous HCl (116 μL, 2 equiv.). The resulting suspension was heated briefly to boiling until the starting material completely dissolved and then concentrated to ~0.3 mL under a stream of argon. Methanol (0.5 mL), 1 M aqueous DABCO

(0.7 mL) and THF (0.3 mL) were added, and the mixture was stirred overnight and then concentrated to dryness. The product was isolated as the ammonium salt by HPLC (30x1 cm R-18 column, ambient temperature, gradient of acetonitrile (27-33%) in 0.5% aqueous NH_4HCO_3 over 20 min, 4 mL/min flow rate, t_R = 15.3 min) to give a pale yellow glassy solid after drying under high vacuum. Isolated yield 38 mg (37 μmol) separated from 83% of the total crude material, 77%. ^{1}H NMR (CD_3OD) δ 1.62 (p, J = 7.0 Hz, 2H), 1.93 (s, 3H), 2.09 (s, 3H), 2.39-2.52 (m, 2H), 2.66 (s, 2H), 2.72 (s, 3H), 2.73 (t, J = 7.0 Hz, 2H), 2.98-3.02 (m, 4H), 3.03 (s, 3H), 3.20 (dd, J = 17.6, 5.6 Hz, 1H), 3.21 (s, 2H), 3.37-3.41 (m, 2H), 3.59 (m, 4H), 3.93 (dd, J = 17.6, 12.1 Hz, 1H), 4.12-4.16 (m, 2H), 5.41 (dd, J = 12.1, 5.6 Hz, 1H), 6.72 (d, J = 8.6 Hz, 1H), 7.11 (dd, J = 8.5, 2.2 Hz, 1H), 7.21 (ad, J = 9.0 Hz, 2H), 7.24 (d, J = 2.2 Hz, 1H), 7.44-7.47 (m, 1H), 7.50 (t, $J \approx$ 7.7 Hz, 1H), 7.59 (ad, J = 9.1 Hz, 2H), 7.80 (br. t, J = 1.7 Hz, 1H), 7.85 (dt, J = 7.6, 1.4 Hz, 1H). ^{13}C NMR (CD_3OD) δ 13.8, 16.3, 28.0, 32.0, 32.3, 24.5, 36.3, 36.6, 42.9, 44.7, 45.7, 46.3, 49.4, 50.0, 63.0, 63.3, 110.3, 112.6, 117.4, 125.2, 125.4, 126.1, 127.4, 128.6, 128.7, 129.0, 129.5, 133.3, 133.9, 139.8, 147.4, 149.2, 149.5. ESI-HRMS m/z calcd for $[M]^{2-}$ $C_{37}H_{51}N_5O_{12}S_7$ 490.5795, found 490.5787.

1.2 Synthesis of Reference Compound (±)-3

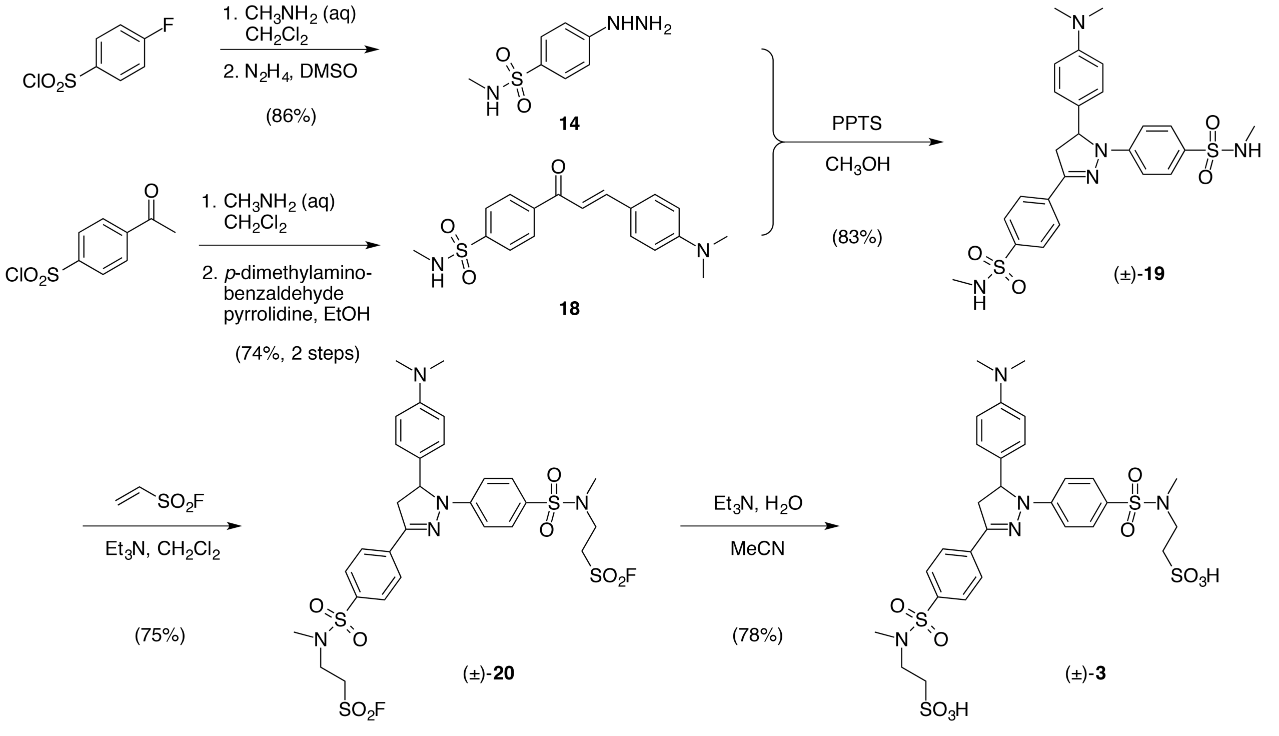

Scheme S2: Overview for the synthesis of reference compound (±)-**3**.

4-Hydrazinyl-*N*-methylbenzenesulfonamide **14**. A solution of 4-fluorobenzenesulfonyl chloride (8.49 g, 43.6 mmol) in dichloromethane (60 mL) was cooled in an ice bath and methylamine solution (7 ml, 40% aqueous, d = 0.9 g/mL, 4 equiv.) was added slowly under stirring. Gentle boiling occurred, and the ice bath was removed once this had subsided. After 15 minutes, the mixture was diluted with crushed ice and carefully acidified with concentrated HCl (10 mL). The organic layer was separated, and the aqueous layer was extracted with dichloromethane (2 x 20 mL). The combined organic layers were dried with Na_2SO_4 and concentrated. The residue was transferred to a 50 mL round-bottom flask and dissolved in DMSO (12 mL). The flask was sealed under argon, and hydrazine (4.1 mL, 3.0

equiv.) was added. The reaction vessel was vented to an oil bubbler, and the mixture was stirred at 50°C overnight. A further 2 mL (1.5 equiv.) of hydrazine were added, and the mixture was stirred at 60°C for 24 hours. After cooling, the mixture was slowly diluted with cold water (100 mL), and the resulting precipitate was collected by filtration, washed with cold water, and recrystallized from ethanol to give the product **14** as colorless needles. Yield 7.54 g (37.5 mmol, 86%). Mp 140-141°C. ^{1}H NMR (DMSO-d$_6$) δ 2.32 (d, *J* = 5.2 Hz, 3H), 4.19 (br. s, 2H), 6.82 (ad, *J* = 8.9 Hz, 2H), 6.94 (q, *J* = 5.2 Hz, 1H), 7.45 (ad, *J* = 8.9 Hz, 2H), 7.54 (br. s, 1H). ^{13}C NMR (DMSO-d$_6$) δ 28.7, 110.0, 124.5, 128.3, 155.2. EI-MS *m/z* 201 ([M]$^+$, 100), 171(50), 123 (55), 107 (60), 90 (40). EI-HRMS *m/z* calcd for $C_7H_{11}N_3O_2S$ 201.0572, found 201.0574.

4-Acetyl-*N*-methylbenzenesulfonamide **17**. A solution of 4-acetylbenzenesulfonyl chloride (4.52 g, 20.7 mmol) in dichloromethane (45 mL) was cooled in an ice bath, and methylamine solution (7 ml, 40% aqueous, d = 0.9 g/mL, 4 equiv.) was added slowly under rapid stirring. After 20 minutes, the solution was acidified with aqueous HCl, and the organic layer was separated. The aqueous layer was extracted with dichloromethane (2 x 15 mL), and the combined organic layers were dried with $MgSO_4$ and concentrated. The residue was recrystallized from boiling ethyl acetate-cyclohexane under slow stirring to give the product **7** as a colorless crystalline powder. Yield 4.09 g (19.2 mmol, 93%). ^{1}H NMR (CDCl$_3$) δ 2.67 (s, 3H), 2.69 (d, *J* = 5.3 Hz, 3H), 4.92 (br, q, *J* = 5.2 Hz, 1H), 7.95-7.98 (m, 2H), 8.08-8.11 (m, 2H). ^{13}C NMR (CDCl$_3$) δ 26.9, 29.3, 127.4, 128.9, 140.0, 142.7, 196.9. EI-MS *m/z* 213 ([M]$^+$, 55), 198 (100), 134 (25), 119 (24), 91(20), 76 (27). EI-HRMS *m/z* calcd for $C_9H_{11}NO_3S$ 213.0460, found 213.0461.

Chalcone **18**. Acetophenone derivative **7** (480 mg, 2.25 mmol) and 4-*N,N*-dimethylamino-benzaldehyde (320 mg, 2.14 mmol) were stirred in ethanol (4 mL) at 50°C until completely dissolved, and pyrrolidine (180 µL, 2.14 mmol) was then added. After 1 hour, the deep red solution was cooled under rapid stirring to initiate crystallization of the product, and the resulting orange slurry was stirred overnight at 50°C. After cooling, the product was collected by filtration, washed with cold ethanol, and dried by suction and then under vacuum to give an orange crystalline powder. Yield 583 mg (1.69 mmol, 79%). Mp 153-154°C. ^{1}H NMR (CDCl$_3$) δ 2.70 (s, 3H), 3.05 (s, 6H), 4.88 (br, s, 1H), 6.68 (ad, *J* = 9.0 Hz, 2H), 7.26 (d, *J* = 15.4 Hz, 1H), 7.55 (ad, *J* = 8.9 Hz, 2H), 7.79 (d, *J* = 15.4 Hz, 1H), 7.97 (ad, *J* = 8.6 Hz, 2H), 8.08 (ad, *J* = 8.6 Hz, 2H). ^{13}C NMR (CDCl$_3$) δ 29.3, 40.0, 111.8, 116.0, 122.0, 127.3, 128.8, 130.8, 141.6, 142.6, 147.5, 152.4, 189.5. EI-MS *m/z* 344 ([M]$^+$, 100), 343 (25), 250 (24), 174 (33).

Pyrazoline derivative (±)-**19.** A mixture of chalcone **18** (393 mg, 1.14 mmol), arylhydrazine **14** (321 mg, 1.4 equiv.), PPTS (400 mg, 1.4 equiv.) and methanol (4 mL) was stirred under argon in a sealed vessel at 90°C for 3 hours. The mixture was poured into water (50 mL) and an attempt was made to extract the product with toluene (50 mL). A large amount of insoluble material remained so MTBE (25 mL) and dichloromethane (25 mL) were added, resulting two clear liquid phases after agitation and settling. The organic layer (top) was separated, dried with Na_2SO_4, and concentrated. The residue was subjected to column chromatography (DCM-MTBE) to give the product as a yellow glassy solid containing 0.6 molar equiv. of MTBE by ^{1}H NMR. Crystallization from dichloromethane-hexane under stirring gave a yellow-green, strongly fluorescent crystalline powder. Yield 501 mg (0.949 mmol, 83%) Mp ~155°C (dec.). ^{1}H NMR (CDCl$_3$) δ 2.59 (d, *J* = 5.4 Hz, 3H), 2.66 (d, *J* = 5.4 Hz, 3H), 2.92 (s, 6H), 3.19 (dd, *J* = 17.3, 6.0 Hz, 1H), 3.84 (dd, *J* = 17.3, 12.3 Hz, 1H), 4.48 (q, *J* = 5.4 Hz, 1H), 4.78 (q, *J* = 5.4 Hz, 1H), 5.34 (dd, *J* = 12.3, 6.0 Hz, 1H), 6.66 (ad, *J* = 8.8 Hz, 2H), 7.09 (ad, *J* = 8.8

Hz, 2H), 7.16 (ad, J = 9.0 Hz, 2H), 7.63 (ad, J = 9.0 Hz, 2H), 7.83-7.88 (m, 4H). ^{13}C NMR ($CDCl_3$) δ 29.27, 29.29, 40.4, 43.3, 63.7, 112.9, 113.0, 126.3, 126.5, 127.5, 127.6, 128.0, 128.7, 136.4, 138.5, 146.9, 147.4, 150.2. MALDI-HRMS calcd for $[M]^+$ $C_{25}H_{29}N_5O_4S_2$ 527.1661, found 527.1671.

Bis(sulfonyl fluoride) derivative (±)-**20.** Triarylpyrazoline (±)-**19** (102 mg, 193 μmol) and triethylamine (54 μL, 2 equiv.) were stirred in dry dichloromethane (4 mL) under argon. Ethenesulfonyl fluoride (48 μL, d = 1.32 g/mL, 3.0 equiv.) was added. TLC (10:1 dichloromethane-MTBE) indicated almost complete consumption of the starting material (*Rf* 0.2) after 8 minutes and formation of two products (*Rf* 0.5 and 0.8). The latter is the desired product and the former is presumably the mono-N-alkylated intermediate. After 4 hours, the starting material was completely consumed but the intermediate remained. After 18 hours the reaction had progressed very little since the 4 hour point, so a further 3 equiv. of ethenesulfonyl fluoride were added. After 10 minutes, the intermediate at *Rf* 0.5 had been completely consumed. The mixture was diluted with toluene (3 mL) and evaporated to dryness under a stream of argon in a 40°C bath. The residue was subjected to column chromatography (2:1 dichloromethane-hexanes with a gradient from 0 to 3.3% MTBE) to give the product as a yellow glassy solid containing 0.74 molar equiv. (8 mass%) of MTBE calculated from the ^{1}H NMR integrals. Yield 117 mg raw (108 mg corrected for solvent content, 145 μmol, 75%). ^{1}H NMR ($CDCl_3$) δ 2.80 (s, 3H), 2.89 (s, 3H), 2.94 (s, 6H), 3.21 (dd, J = 17.4, 6.0 Hz, 1H), 3.47-3.51 (m, 2H), 3.55-3.59 (m, 2H), 3.69-3.79 (m, 4H), 3.87 (dd, J = 17.4, 12.3 Hz, 1H), 5.37 (dd, J = 12.3, 6.0 Hz, 1H), 6.68 (ad, J = 8.9 Hz, 2H), 7.10 (ad, J = 8.8 Hz, 2H), 7.19 (ad, J = 9.0 Hz, 2H), 7.59 (ad, J = 9.1 Hz, 2H), 7.83 (ad, J = 8.7 Hz, 2H), 7.90 (ad, J = 8.7 Hz, 2H). ^{19}F NMR ($CDCl_3$) δ 56.3 (t, J = 4.8 Hz, 1F), 56.6 (t, J = 4.7 Hz, 1F). ESI-HRMS calcd for $[M+H]^+$ $C_{29}H_{36}O_8N_5F_2S_4$ 748.1409, found 748.1405.

Reference triarylpyrazoline (±)-**3.** Sulfonyl fluoride (±)-**20** (74 mg of material containing 8% MTBE, 91 μmol) was stirred in 1 mL of a mixture containing acetonitrile (45%), triethylamine (45%) and water (10 %). After 10 min, TLC (10:1 dichloromethane-MTBE) indicated a mixture of the starting material (*Rf* 0.8), a small amount of single-elimination product (*Rf* 0.5) and a trace of double elimination product (*Rf* 0.2, identical to triarylpyrazoline (±)-**19**), as well as hydrolysis products (*Rf* ≈0). After stirring overnight, only the hydrolysis products and double-elimination product remained. The mixture was concentrated to dryness under a stream of argon, and the residue was taken up in methanol (2 mL) + concentrated ammonia (~100 μL) and concentrated again. The residue was dissolved in water (2 mL), and the product was isolated by RP-HPLC using a gradient of 25-33% CH3CN in 0.1% aqueous $(NH_4)HCO_3$ to give a yellow glassy solid after repeated evaporation with methanol and vacuum drying. The ^{1}H NMR spectrum of this material is consistent with a mixed ammonium-triethylammonium salt containing 44 mol% Et_3NH^+, corresponding to a formula weight of 815 g/mol. Yield 74 mg (71 μmol, 78%). ^{1}H NMR (CD_3OD) δ 1.30 (t, J = 7.3 Hz, 4H (0.44 Et_3NH^+)) 2.72 (s, 3H), 2.83 (s, 3H), 2.90 (s, 6H), 2.98-3.04 (m, 4H), 3.19 (dd, J ≈ 17.7, 5.8 Hz, 1H, partly obscured by subsequent signal), 3.20 (q, J = 7.3 Hz, 2.6H (0.44 Et_3NH^+)), 3.34-3.43 (m, 2H), 3.47-3.51 (m, 2H), 3.95 (dd, J = 17.6, 12.2 Hz, 1H), 5.48 (dd, J = 12.2, 5.8 Hz, 1H), 6.76 (ad, J = 8.8 Hz, 2H), 7.13 (ad J = 8.8 Hz, 2H), 7.23 (ad, J = 9.0 Hz, 2H), 7.58 (ad, J = 9.0 Hz, 2H), 7.86 (ad, J = 8.7 Hz, 2H), 7.99 (ad, J = 8.7 Hz, 2H). ^{13}C NMR (CD_3OD) δ 35.8, 35.9, 41.1, 44.2, 47.67, 47.70, 50.63, 50.74, 64.9, 114.2, 114.9, 127.1, 127.72, 127.74, 128.9, 129.9, 131.0, 138.1, 138.5, 148.6, 149.6, 151.6 (prior to acquisition of the ^{13}C NMR spectrum, Et_3N was removed by extraction with MTBE from a solution of the mixed salt in dilute aqueous ammonia). ESI-HRMS *m/z* calcd for $[M]^{2-}$ $C_{29}H_{35}N_5O_{10}S_4$ 370.5639, found 370.5630.

1.3 Synthesis of Pyrazoline Derivative (±)-4

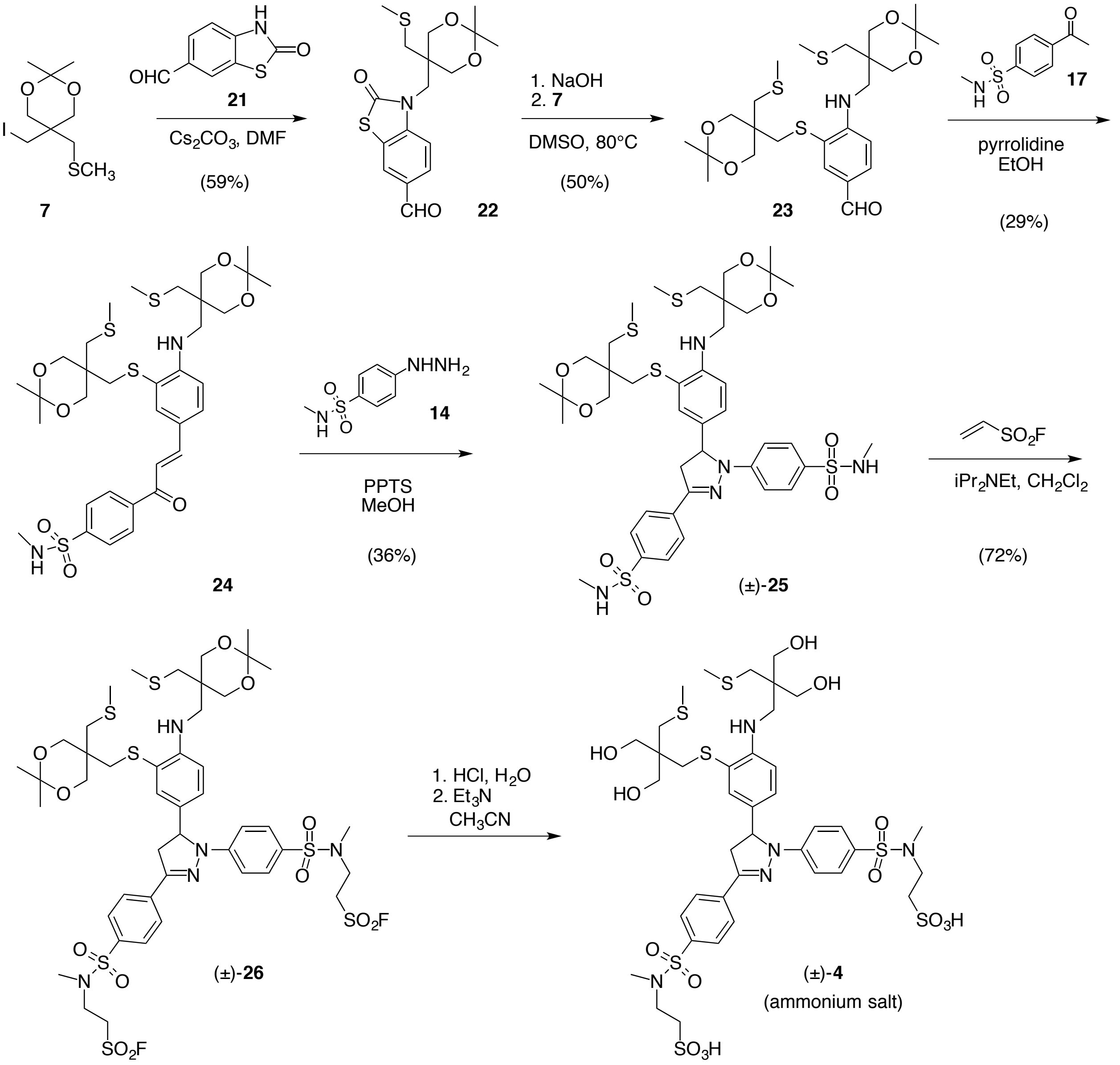

Scheme S3: Overview for the synthesis of pyrazoline derivative (±)-**4**.

Benzothiazolinone **22**. A mixture of benzothiazolin-2-one-6-carboxaldehyde[4] (**21**, 255 mg, 1.42 mmol), iodide **7** (562 mg, 1.25 equiv.), and K_2CO_3 (600 mg, 3 equiv.) in DMF (5 mL) was stirred at 90°C for 12 hours. The mixture was diluted into a solution of 1 M NaOH in 20% aqueous methanol (100 mL), and the resulting emulsion was extracted with MTBE (100 mL). The extract was washed with a further 100 mL of the aqueous-methanolic NaOH solution, dried with Na_2SO_4, and concentrated under reduced pressure to a yellow oily residue. Crystallization from diethyl ether-pentane under stirring gave the product as a slightly tan crystalline powder. Yield 306 mg (833 μmol, 59%). Mp 134-134.5°C. ^{1}H NMR ($CDCl_3$) δ 1.49 (br. s, 6H), 2.13 (s, 3H) 2.66 (s, 2H), 3.64 (d, *J* = 12.5 Hz, 2H), 4.03 (d, *J* = 12.5 Hz, 2H), 4.24 (s, 2H), 7.74 (d, *J* = 8.5 Hz, 1H), 7.87 (dd, *J* = 8.5, 1.6 Hz, 1H), 7.97 (d, *J* = 1.6 Hz, 1H), 9.95 (s, 1H). ^{13}C NMR ($CDCl_3$) δ 18.4, 21.0, 26.7, 38.1, 40.1, 46.6, 64.7, 98.6, 111.7,

123.3, 123.4, 129.4, 132.0, 143.1, 171.4, 190.2. EI-HRMS *m/z* calcd for C_{17} $H_{21}NO_4S_2$ 367.0912, found 367.0921.

Aldehyde **23**. A solution of intermediate **22** (253 mg, 688 µmol) in DMSO (10 mL) was heated to 80°C under argon, and 15% aqueous NaOH (830 µL, 4.5 equiv.) was added under rapid stirring. After 50 minutes, iodide **7** (272 mg, 1.25 equiv. was added as a solution in DMSO (2 mL). After 30 minutes, the mixture was partitioned between water (80 mL) and MTBE (80 mL). The organic layer was separated, washed with twice with water (80 mL) plus brine (10 mL), dried with $MgSO_4$, and concentrated. The residue was separated by column chromatography (hexanes-MTBE) to give the product **23** as a pale yellow oil. Yield 181 mg (342 µmol, 50%). ^{1}H NMR ($CDCl_3$) δ 1.39 (s, 3H), 1.41 (s, 3H), 1.46 (s, 3H), 1.48 (s, 3H), 2.12 (s, 3H), 2.17 (s, 3H), 2.59 (s, 2H), 2.75 (s, 2H), 2.98 (s, 2H), 3.58 (d, *J* = 6.6 Hz, 2H), 3.71 (d, *J* = 12.2 Hz, 2H), 3.77 (s, 4H), 3.83 (d, *J* = 12.2 Hz, 2H), 6.11 (t, *J* = 6.6 Hz, 1H), 6.93 (d, *J* = 8.6 Hz, 1H), 7.71 (dd, *J* = 8.6, 2.0 Hz, 1H), 7.98 (d, *J* = 2.0 Hz, 1H), 9.69 (s, 1H). ^{13}C NMR ($CDCl_3$) δ 17.7, 21.1, 23.6, 23.7, 26.3, 38.0, 38.1, 38.5, 38.8, 38.9, 45.4, 65.3, 66.1, 66.2, 98.5, 98.6, 109.4, 118.0, 126.3, 133.0, 138.1, 154.0, 189.6. EI-MS m/z 529 ($[M]^+$, 100), 354 (25), 180 (27), 164 (40), 117 (55), 61 (50). EI-HRMS *m/z* calcd for $[M]^+$ $C_{25}H_{39}NO_5S_3$ 529.1990, found 529.1993.

Chalcone **24**. A solution of aldehyde **23** (764 mg, 1.44 mmol), acetophenone derivative **17** (308 mg, 1.44 mmol), and pyrrolidine (241 µL, 2.88 mmol) in ethanol (6 mL) was stirred for 2 days at 50°C. The resulting oily biphasic mixture was poured into water (50 mL) + 1 M NaH_2PO_4 (10 mL) and extracted with MTBE (60 mL). The extract was dried with $MgSO_4$ and concentrated, and the residue was separated by column chromatography (2:1 DCM-hexanes + 0→17% MTBE) to give the product as an orange-red glassy solid. Yield 305 mg (420 µmol, 29%). ^{1}H NMR ($CDCl_3$) δ 1.39 (s, 3H), 1.42 (s, 3H), 1.47 (s, 3H), 1.49 (s, 3H), 2.13 (s, 3H), 2.17 (s, 3H), 2.61 (s, 2H), 2.71 (d, *J* = 5.3 Hz, 3H), 2.76 (s, 2H), 3.00 (s, 2H), 3.55 (d, *J* = 6.6 Hz, 2H), 3.72 (d, *J* = 12.1 Hz, 2H), 3.78 (s, 4H), 3.83 (d, *J* = 12.1 Hz, 2H), 4.67 (q, *J* = 5.3 Hz, 1H), 5.89 (t, *J* = 6.6 Hz, 1H), 6.91 (d, *J* = 8.7 Hz, 1H), 7.29 (d, *J* = 15.5 Hz, 1H), 7.53 (dd, *J* = 8.7, 2.1 Hz, 1H), 7.73 (d, *J* = 15.5 Hz, 1H), 7.80 (d, *J* = 2.1 Hz 1H), 7.98 (ad, *J* = 8.6 Hz, 2H), 8.11 (ad, *J* = 8.6 Hz, 2H). ^{13}C NMR ($CDCl_3$) δ 17.8, 21.3, 23.5, 23.9, 26.2, 26.9, 29.3, 38.1, 38.2, 38.6, 38.9, 39.1, 45.5, 65.4, 66.3, 98.5, 98.6, 110.2, 116.8, 118.3, 123.4, 127.3, 128.9, 131.7, 137.0, 141.8, 142.4, 146.5, 151.8, 189.3. EI-MS *m/z* 724 ($[M]^+$, 100), 375 (40), 373 (25), 198 (25), 131 (30), 117 (83), 61 (92). EI-HRMS *m/z* calcd for $[M]^+$ $C_{34}H_{48}N_2O_7S_4$ 724.2344, found 724.2350.

Pyrazoline (±)-**25**. This compound was synthesized according to the procedure described for triarylpyrazoline (±)-**28** using chalcone **24** (162 mg, 223 µmol), PPTS (112 mg, 2 equiv.), and arylhydrazine **14** (90 mg, 2 equiv.). Yield 74 mg (81 µmol, 36%), yellow glassy solid. ^{1}H NMR ($CDCl_3$) δ 1.35 (s, 3H), 1.39 (s, 3H), 1.44 (s, 6H), 2.06 (s, 3H), 2.14 (s, 3H), 2.60-2.63 (m, 5H), 2.66-2.69 (m, 5H), 2.88 (d, *J* = 12.9 Hz, 1H), 2.94 (d, *J* = 12.9 Hz, 1H), 3.19 (dd, *J* = 17.3, 6.0 Hz 1H), 3.41 (dd, *J* = 6.6, 2.1 Hz, 2H), 3.68-3.81 (m, 8H), 3.84 (dd, *J* = 17.3, 12.3 Hz, 1H), 4.37 (q, *J* = 5.5 Hz, 1H), 4.59 (q, *J* = 5.4 Hz, 1H), 5.30 (dd, *J* = 12.3, 6.0 Hz, 1H), 5.33 (t, *J* = 6.6 Hz, 1H), 6.81 (d, *J* = 8.6 Hz, 1H), 7.05 (dd, *J* = 8.6, 2.2 Hz, 1H), 7.16 (ad, *J* = 9.0 Hz 2H), 7.32 (d, *J* = 2.2 Hz 1H), 7.65 (ad, *J* = 9.0 Hz, 2H), 7.84-7.89 (m, 4H). ^{13}C NMR ($CDCl_3$) δ 17.78, 17.84, 21.6, 23.68, 23.73, 25.9, 29.3, 29.4, 29.7, 38.0, 38.3, 38.6, 38.7, 43.2, 45.9, 63.4, 65.3, 65.38, 65.43, 66.2, 98.4, 98.5, 111.1, 113.1, 114.7, 118.6, 126.4, 127.5, 127.6, 127.8, 127.9, 128.75, 128.83, 132.9, 136.4, 138.6, 146.8, 147.4, 149.3. ESI-HRMS calcd for $[M+H]^+$ $C_{41}H_{58}N_5O_8S_5$ 908.2889, found 908.2889.

Bis(sulfonyl fluoride) derivative (±)-**26**. This compound was synthesized as described for sulfonyl fluoride (±)-**20** using triarylpyrazoline (±)-**25** (60 mg, 66 µmol), ethenesulfonyl fluoride (39 µL, 7 equiv.), and diisopropylethylamine (202 µL, 18 equiv.) with a reaction time of 2 hours. Yield 54 mg (48 µmol, 72%), yellow glassy solid. ^{1}H NMR ($CDCl_3$) δ 1.36 (s, 3H), 1.40 (s, 3H), 1.44 (s, 6H), 2.07 (s, 3H), 2.15 (s, 3H), 2.60 (s, 2H), 2.65 (d, *J* = 13.3 Hz 1H), 2.69 (d, *J* = 13.3 Hz, 1H), 2.82 (s, 3H), 2.90 (s, 3H), 2.91 (d, *J* = 12.8 Hz, 1H), 2.96 (d, *J* = 12.8 Hz, 1H), 3.21 (dd, *J* = 17.4, 6.0 Hz, 1H), 3.38-3.46 (m, 2H), 3.49-3.52 (m, 2H), 3.56-3.59 (m, 2H), 3.68-3.82 (m, 12H), 3.86 (dd, *J* = 17.4, 12.2 Hz, 1H), 5.31 (dd, *J* = 12.2, 6.0 Hz, 1H), 5.36 (t, *J* = 6.7 Hz, 1H), 6.82 (d, *J* = 8.6 Hz, 1H), 7.05 (dd, *J* = 8.5, 2.2 Hz, 1H), 7.19 (ad, *J* = 8.9 Hz, 2H), 7.34 (d, *J* = 2.2 Hz, 1H), 7.61 (ad, *J* = 9.1 Hz, 2H), 7.84 (ad, *J* = 8.7 Hz, 2H), 8.00 (ad, *J* = 8.7 Hz, 2H). ^{19}F NMR ($CDCl_3$) δ 56.3 (t, *J* = 4.8 Hz, 1F), 56.6 (t, *J* = 4.7 Hz,1F). ESI-HRMS calcd for $[M+H]^+$ $C_{45}H_{64}N_5O_{12}S_7F_2$ 1128.2559, found 1128.2560.

Pyrazoline probe (±)-**4**. Bis(sulfonyl fluoride) derivative (±)-**26** (50.6 mg, 44 µmol) was dissolved in acetonitrile (1 mL) , and 3 M aqueous HCl (207 µL, 14 equiv HCl, ~260 equiv. H_2O) was added to the stirred solution. After 15 min, triethylamine (350 µL, 56 equiv.) was added. The mixture was stirred overnight and then concentrated to dryness under a stream of nitrogen in a 40°C bath. The residue was taken up in methanol (2 mL) and K_2CO_3 (10 equiv) was added, followed by sufficient water for complete dissolution, and the mixture was concentrated to dryness to remove triethylamine. The residue was taken up in water, filtered, and a portion was purified by HPLC as described for pyrazoline (±)-**19** to give the ammonium salt as a yellow glassy solid. Isolated yield 3.5 mg (3.6 µmol, 8%). ^{1}H NMR (CD_3OD) δ 1.98 (s, 3H), 2.10 (s, 3H), 2.54 (d, *J* = 13.0 Hz, 1H), 2.57 (d, *J* = 13.0 Hz, 1H), 2.69 (s, 2H), 2.73 (s, 3H), 2.79 (d, *J* = 12.7 Hz, 1H), 2.83 (s, 3H), 2.84 (d, *J* ≈ 13 Hz, 1H, obscured by previous signal), 2.97-3.05 (m, 4H), 3.21-3.55 (m, 11H, partly obscured by CHD_2OH and CH_3OH signals), 3.71 (m, 4H), 3.95 (dd, *J* = 17.5, 12.2 Hz, 1H), 5.46 (dd, *J* = 12.2, 5.6 Hz, 1H), 6.72 (d, *J* = 8.6 Hz, 1H), 7.08 (dd, *J* = 8.6, 2.2 Hz, 1H), 7.27 (ad, *J* = 9.0 Hz, 2H), 7.35 (d, *J* = 2.2 Hz, 1H), 7.62 (ad, *J* = 9.1 Hz, 2H), 7.88 (ad, *J* = 8.6 Hz, 2H), 8.01 (ad, *J* = 8.6 Hz, 2H). ESI-HRMS *m/z* calcd for $[M]^{2-}$ $C_{39}H_{55}N_5O_{14}S_7$ 520.5901, found 520.5891.

1.4 Synthesis of Pyrazoline Derivative (±)-5

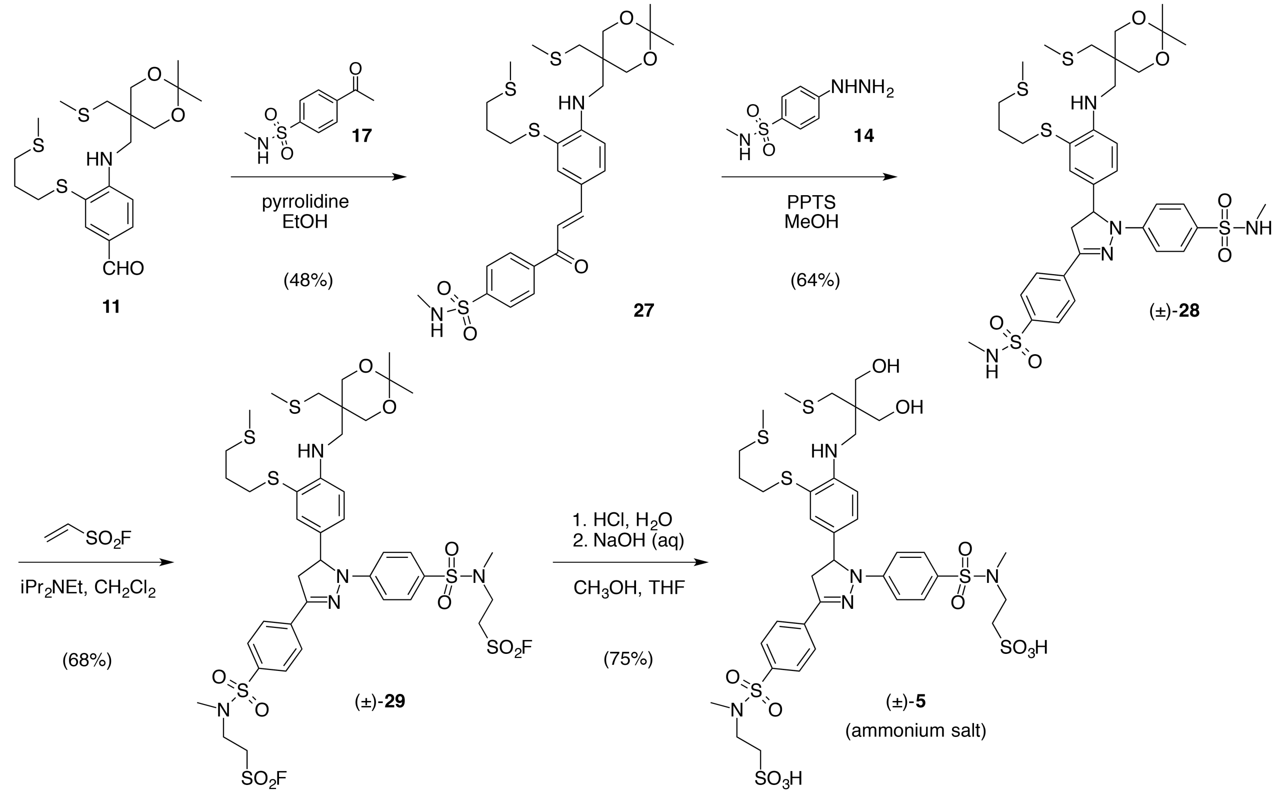

Scheme S4: Overview for the synthesis of pyrazoline derivative (±)-**5**.

Chalcone **27**. A solution of aldehyde **11** (420 mg, 978 μmol), acetophenone derivative **17** (208 mg, 1 equiv.), and pyrrolidine (163 μL, 2 equiv.) in ethanol (5 mL) was stirred at 45°C for two days. The resulting biphasic mixture was partitioned between water (100 mL) + 1 M NaH_2PO_4 (10 mL) and MTBE (100 mL). The organic layer was dried with MgSO4 and concentrated, and the residue was separated by column chromatography (DCM-MTBE) to give the product as a red-orange glassy solid. Yield 296 mg (474 μmol, 48%). ^{1}H NMR ($CDCl_3$) δ 1.47 (s, 3H), 1.49 (s, 3H), 1.85 (p, *J* = 7.1 Hz, 2H), 2.05 (s, 3H), 2.18 (s, 3H), 2.59 (s, 2H), 2.60 (t, *J* = 7.0 Hz, 2H), 2.70 (d, *J* = 5.3 Hz, 3H), 2.84 (t, *J* = 7.1 Hz, 2H), 3.53 (d, *J* = 6.5 Hz, 2H), 3.72 (d, *J* = 12.1 Hz, 2H), 3.83 (d, *J* = 12.1 Hz, 2H), 4.97 (q, *J* = 5.3 Hz, 1H), 5.96 (t, *J* = 6.5 Hz, 1H), 6.89 (d, *J* = 8.7 Hz, 1H), 7.29 (d, *J* = 15.5 Hz, 1H), 7.55 (dd, *J* = 8.7, 2.1 Hz, 1H), 7.74 (d, *J* = 15.4 Hz, 1H), 7.75 (d, *J* = 2.1 Hz, 1H), 7.98 (ad, *J* = 8.5 Hz, 2H), 8.10 (ad, *J* = 8.5 Hz, 2H). ^{13}C NMR ($CDCl_3$) δ 15.3, 17.7, 21.2, 26.2, 28.4, 29.3, 32.7, 33.7, 38.2, 38.7, 45.5, 65.4, 98.6, 110.0, 116.6, 117.6, 123.0, 127.3, 128.8, 131.8, 137.4, 141.7, 142.2, 146.5, 152.0, 189.2. EI-MS *m/z* 624 ($[M]^+$, 70), 449 (25), 89 (100), 61 (25). EI-HRMS *m/z* calcd for $[M]^+$ $C_{29}H_{40}N_2O_5S_4$ 624.1820, found 624.1804.

Triarylpyrazoline (±)-**28**. Chalcone **27** (126 mg, 202 μmol) and PPTS (101 mg, 2 equiv.) were stirred in boiling methanol for 15 min. The mixture was concentrated to dryness under a stream of argon and the residue was dissolved in methanol (1.5 mL). Arylhydrazine **14** (90 mg, 2 equiv.) was added, and the deep red solution was stirred in a sealed vessel under argon at 90°C for 4 hours. The resulting brownish-yellow solution was allowed to cool and concentrated to dryness. The residue was

dissolved in acetone (2 mL) and 2,2-dimethoxypropane (2 mL) and *p*-TsOH • H_2O (108 mg, 2 equiv.) were added. After stirring for 15 minutes, the reaction was quenched with triethylamine (240 μL, 6 equiv.), and the mixture was diluted with DCM (50 mL) and washed with H_2O (50 mL) + 1 M aqueous NaH_2PO_4 (10 mL). The aqueous layer was extracted with DCM (20 mL), and the combined organic layers were dried with Na_2SO_4 and concentrated to dryness. The residue was separated by column chromatography (DCM-MTBE) to give 105 mg (129 μmol, 64%) of the product as yellow glassy solid. ^{1}H NMR ($CDCl_3$) δ 1.44 (s, 6H), 1.69 (p, *J* = 7.1 Hz, 2H), 2.00 (s, 3H), 2.15 (s, 3H), 2.44-2.55 (m, 2H), 2.59 (s, 2H), 2.61 (d, *J* = 5.5 Hz, 3H), 2.68 (d, *J* = 5.4 Hz, 3H), 2.74 (t, *J* = 7.1 Hz, 2H), 3.19 (dd, *J* = 17.4, 5.9 Hz, 1H), 3.35-3.43 (m, 2H), 3.70 (d, *J* = 12.0 Hz, 2H), 3.79 (d, *J* ≈ 12 Hz, 2 H), 3.85 (dd, *J* = 17.4, 12.3 Hz, 1H), 4.42 (q, *J* = 5.5 Hz, 1H), 4.66 (q, *J* = 5.4 Hz, 1H), 5.31 (dd, *J* = 12.3, 5.9 Hz, 1H), 5.39 (t, *J* = 6.6 Hz, 1H), 6.80 (d, *J* = 8.6 Hz, 1H), 7.08 (dd, *J* = 8.5, 2.2 Hz, 1H), 7.15 (ad, *J* = 9.0 Hz, 2H), 7.25 (d, *J* = 2.2 Hz, 1H), 7.65 (ad, *J* = 9.1 Hz, 2H), 7.84-7.90 (m, 4H). ^{13}C NMR ($CDCl_3$) δ 15.4, 17.8, 21.6, 25.9, 28.4, 29.30, 29.34, 32.6, 33.4, 38.3, 38.6, 43.2, 45.9, 63.4, 65.4, 65.5, 98.6, 110.9, 113.0, 117.8, 126.4, 127.6, 127.6, 127.9, 128.5, 128.7, 133.3, 136.3, 138.6, 146.7, 147.4, 149.5. MALDI-HRMS m/z calcd for $[M+H]^+$ $C_{36}H_{50}N_5O_6S_5$ 808.2365, found 808.2395.

Bis(sulfonyl fluoride) derivative (±)-**29**. This compound was synthesized according to the procedure described for bis(sulfonyl fluoride) derivative (±)-**20** using triarylpyrazoline (±)-**28** (78 mg, 97 μmol), ethenesulfonyl fluoride (40 μL, 5 equiv.), and diisopropylethylamine (211 μL, 12.6 equiv.). Yield 67 mg (65 μmol, 68%). ^{1}H NMR ($CDCl_3$) δ 1.44 (s, 6H), 1.71 (p, *J* = 7.1 Hz, 2H), 2.01 (s, 3H), 2.15 (s, 3H), 2.49-2.56 (m, 2H), 2.58 (s, 2H), 2.75 (t, *J* = 7.1 Hz, 2H), 2.82 (s, 3H), 2.90 (s, 3H), 3.21 (dd, *J* = 17.4, 5.9 Hz, 1H), 3.40 (d, *J* = 6.7 Hz, 2H) 3.49-3.53 (m, 2H), 3.56-3.59 (m, 2H), 3.68-3.81 (m, 8H), 3.87 (dd, *J* = 17.4, 12.3 Hz, 1H), 5.32 (dd, *J* = 12.3, 5.9 Hz, 1H), 5.42 (t, *J* = 6.6 Hz, 1H), 6.81 (d, *J* = 8.6 Hz, 1H), 7.08 (dd, *J* = 8.5, 2.2 Hz, 1H), 7.19 (ad, *J* = 9.0 Hz, 2H), 7.26 (d, *J* = 2.2 Hz, 1H), 7.61 (ad, *J* = 9.1 Hz, 2H), 7.84 (ad, *J* = 8.7 Hz, 2H), 7.90 (ad, *J* = 8.7 Hz, 2H). ^{19}F NMR δ 56.3 (t, *J* = 4.8 Hz, 1F), 56.6 (t, *J* = 4.7 Hz, 1F). MALDI-HRMS m/z calcd for $[M+H]^+$ $C_{40}H_{56}N_5O_{10}S_7F_2$ 1028.2040, found 1028.2002.

Pyrazoline probe (±)-**5**. Sulfonyl fluoride (±)-**29** (48 mg, 47 μmol) was stirred in a mixture of methanol (1.5 mL), THF (0.5 mL), and 3 M aqueous HCl (47 μL, 3 equiv.). After 40 min, the mixture was concentrated to dryness under a stream of argon, and the residue was stirred in a mixture of methanol (2 mL), THF (1 mL), and 5 M NaOH (112 μL, 12 equiv.). After 4 hours, the reaction was quenched by adding a small piece of dry ice and concentrated to dryness. The residue was taken up in methanol (5 mL), filtered through cotton to remove $NaHCO_3$, and concentrated to dryness. The resulting material was taken up in 75% H_2O/25% CH_3CN and separated by RP-HPLC using a gradient of 30-34% CH_3CN in 0.1% aqueous $(NH_4)HCO_3$ to give the product ammonium salt as a yellow glassy solid after drying under high vacuum. Purification of 37.5% of the total crude material gave 13.3 mg (13 μmol, 75% yield). ^{1}H NMR (CD_3OD) δ 1.62 (p, *J* = 7.0 Hz, 2H), 1.93 (s, 3H), 2.09 (s, 3H), 2.39-2.52 (m, 2H), 2.66 (s, 2H), 2.71-2.74 (m, 5H), 2.83 (s, 3H), 2.97-3.04 (m, 4H), 3.21 (s, 2H), 3.22 (dd, *J* = 17.5, 5.7 Hz, 1H), 3.36-3.44 (m, 2H), 3.47-3.51(m, 2H), 3.59 (s, 4H), 3.94 (dd, *J* = 17.5, 12.2 Hz, 1H), 5.47 (dd, *J* = 12.2, 5.6 Hz, 1H), 6.73 (d, *J* = 8.6 Hz, 1H), 7.12 (dd, *J* = 8.5, 2.2 Hz, 1H), 7.23-7.25 (m, 3H), 7.61 (ad, *J* = 9.0 Hz, 2H), 7.87 (ad, *J* = 8.5 Hz, 2H), 8.00 (ad, *J* = 8.5 Hz, 2H). ^{13}C NMR (CD_3OD) δ 15.3, 17.7, 29.5, 33.4, 33.8, 35.9, 36.0, 37.8, 44.1, 46.2, 47.2, 47.7, 47.8, 50.7, 50.8, 64.7, 64.8, 111.7, 114.3, 118.9, 127.3, 127.8, 128.90, 128.95, 129.90, 130.0, 134.7, 138.1, 138.6, 148.6, 149.8, 151.1. ESI-HRMS *m/z* calcd for $[M]^{2-}$ $C_{37}H_{51}N_5O_{12}S_7$ 490.5795, found 490.5787.

^{1}H-NMR (CD$_3$OD, 400 MHz)

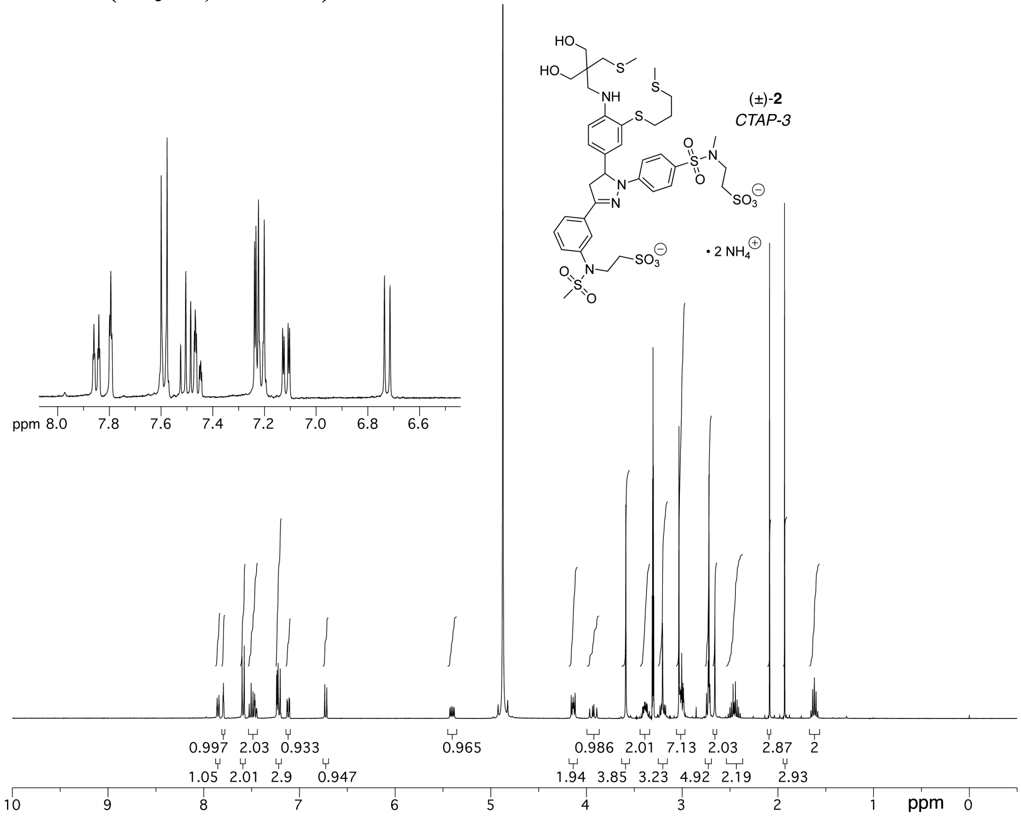

^{13}C-NMR (CD$_3$OD, 100 MHz)

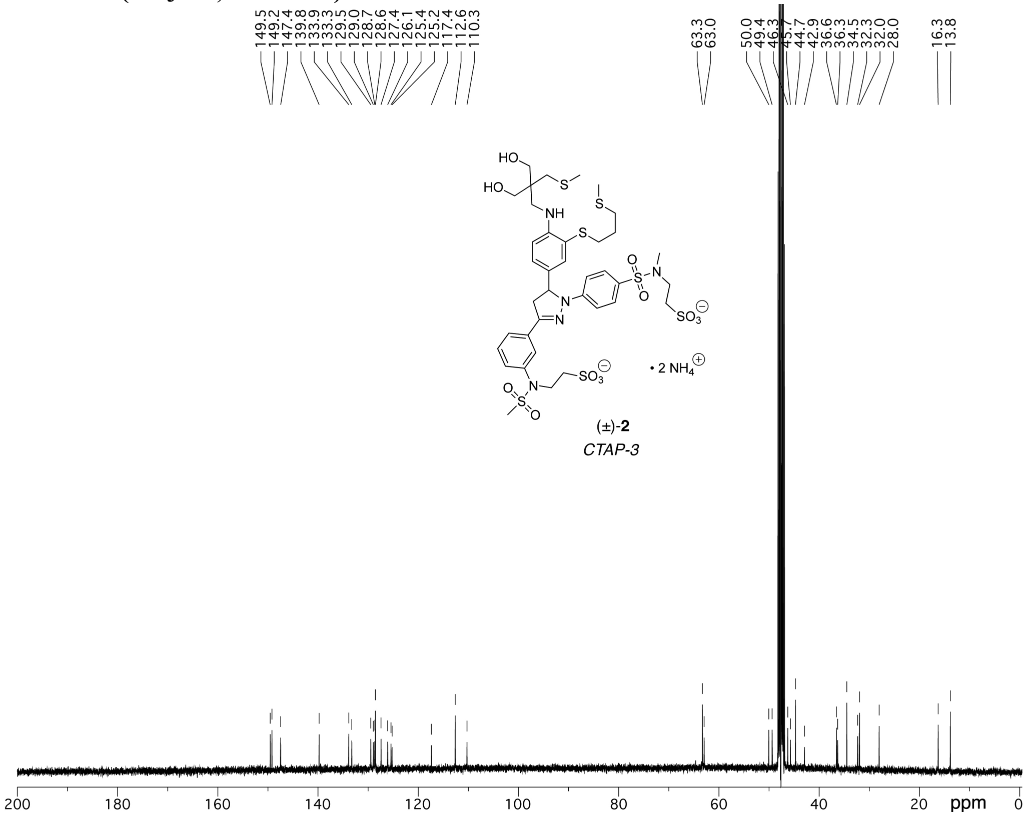

^{1}H-NMR (CD_3OD, 400 MHz)

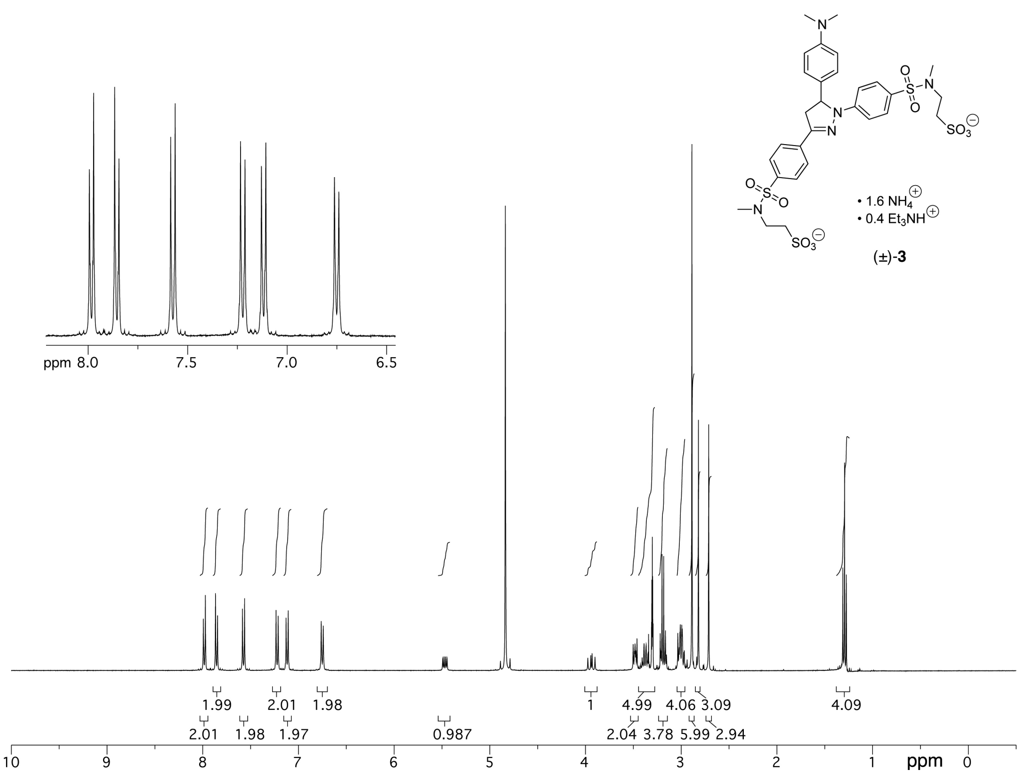

^{13}C-NMR (CD_3OD, 100 MHz)

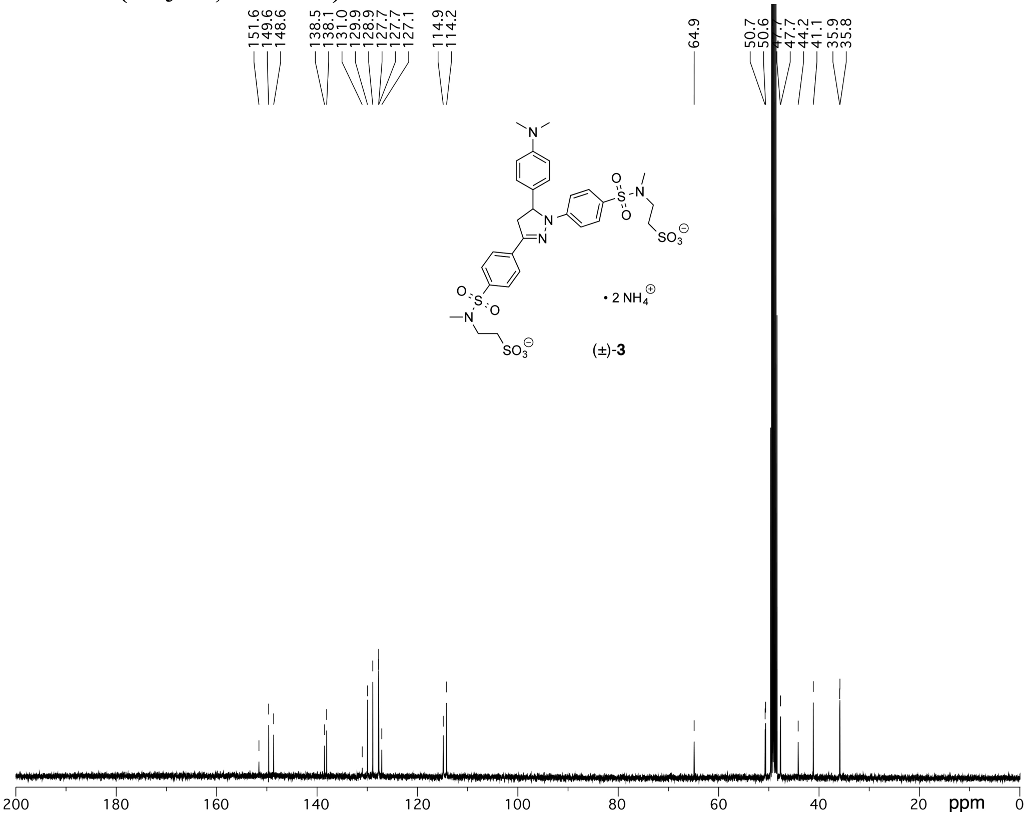

^{1}H-NMR (CD$_3$OD, 400 MHz)

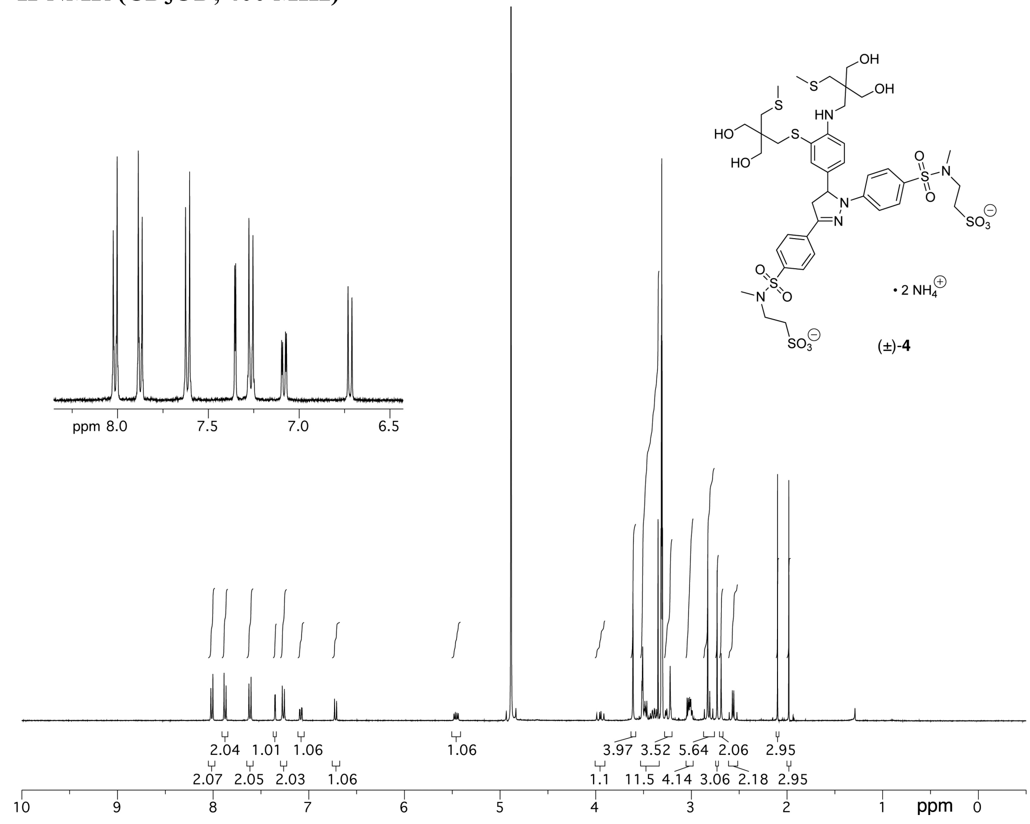

(±)-**4**

^{1}H-NMR (CD$_3$OD, 400 MHz)

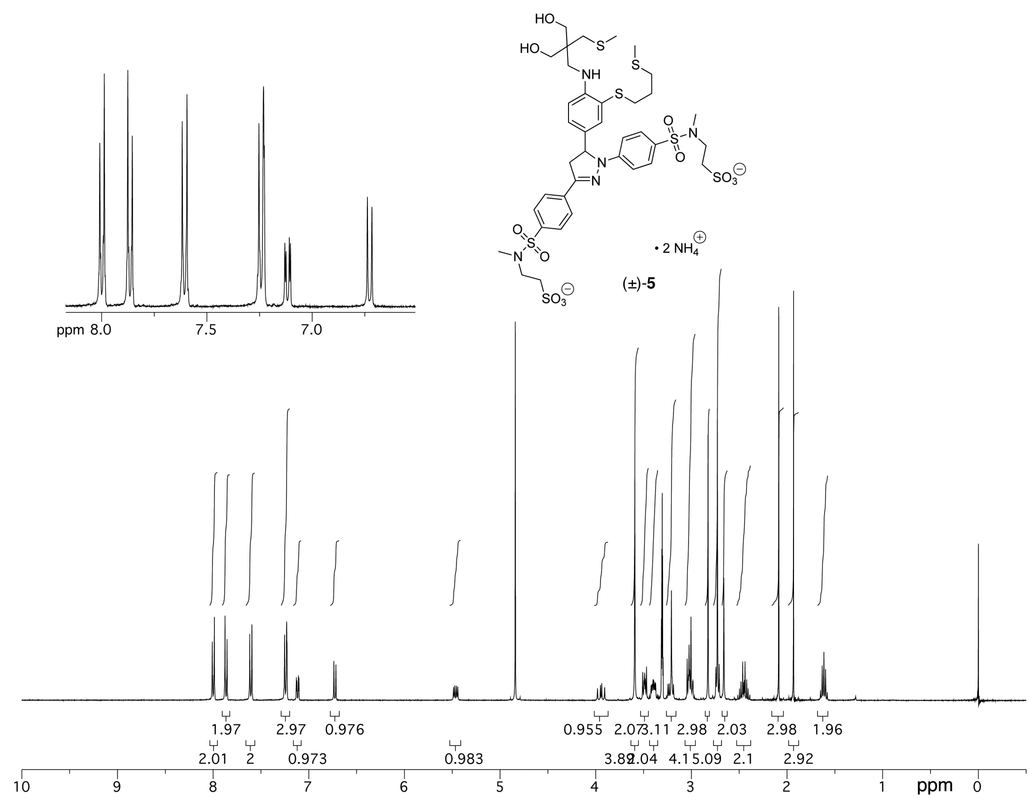

(±)-**5**

^{1}H-NMR ($CDCl_3$, 400 MHz)

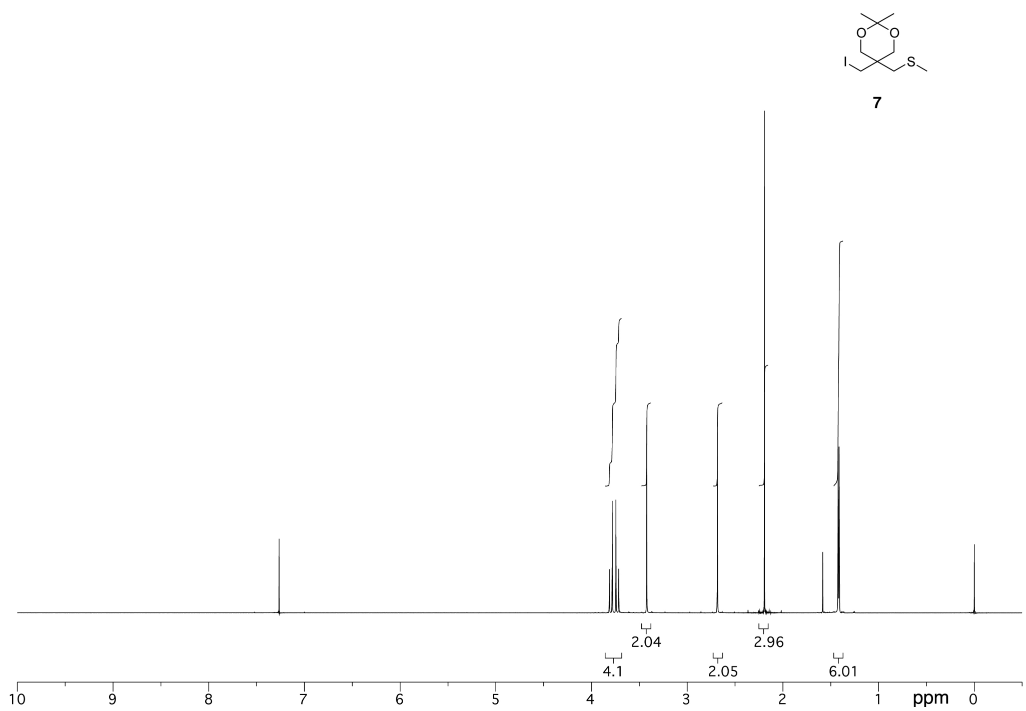

^{13}C-NMR ($CDCl_3$, 100 MHz)

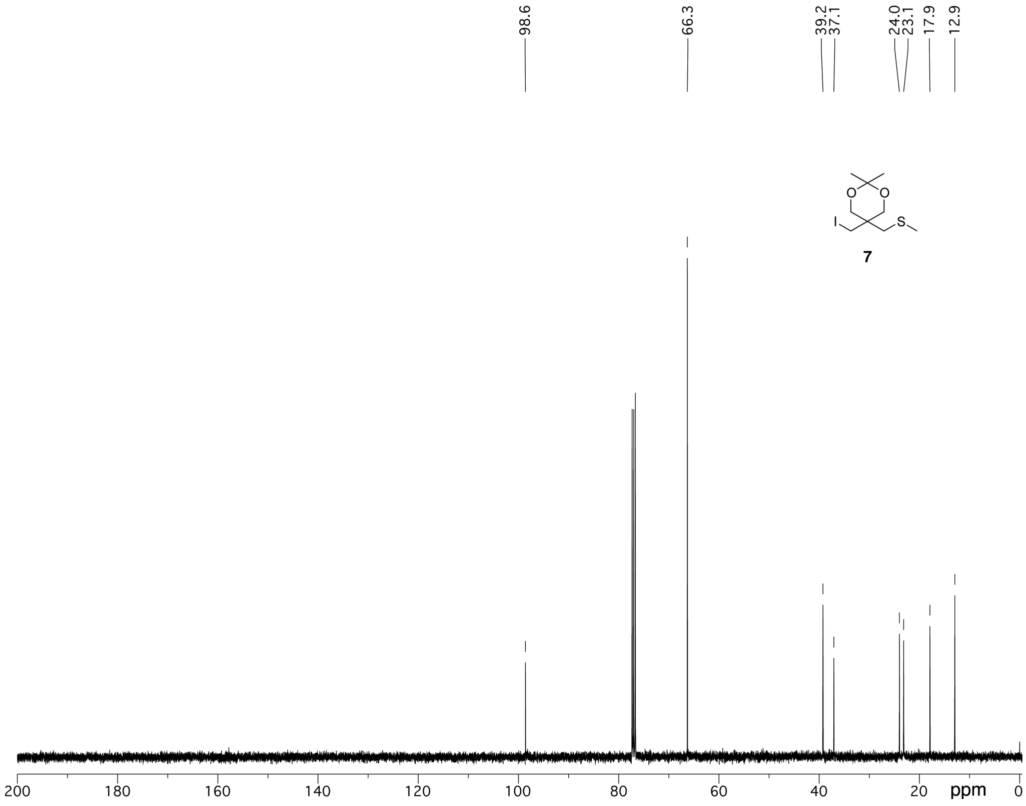

^{1}H-NMR ($CDCl_3$, 400 MHz)

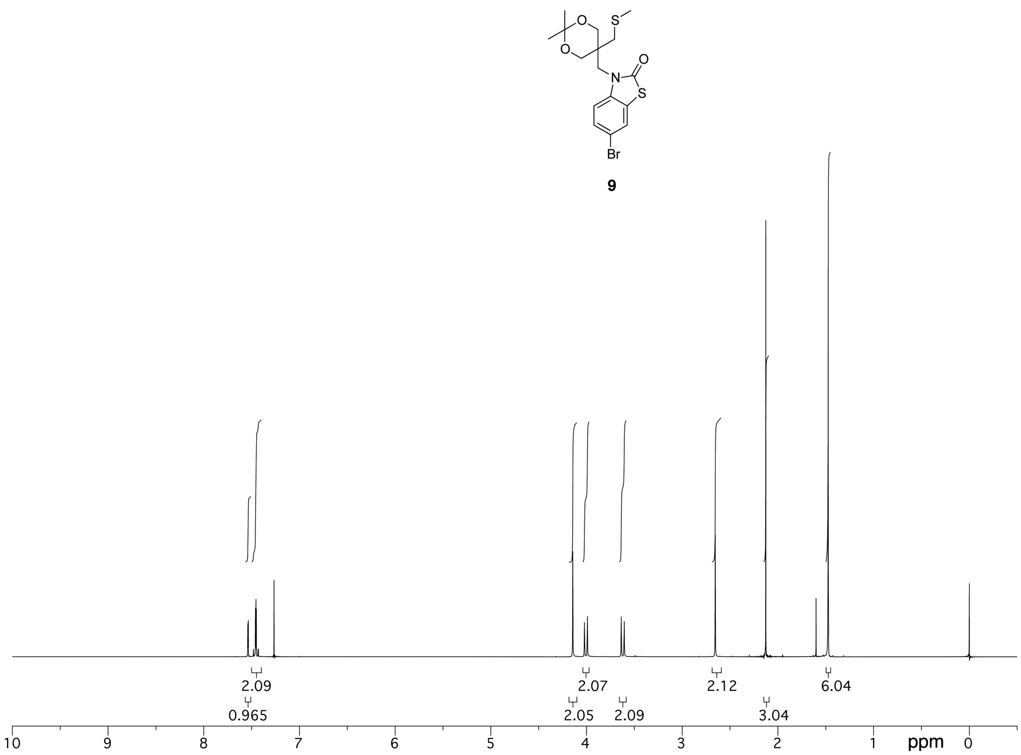

^{13}C-NMR ($CDCl_3$, 100 MHz)

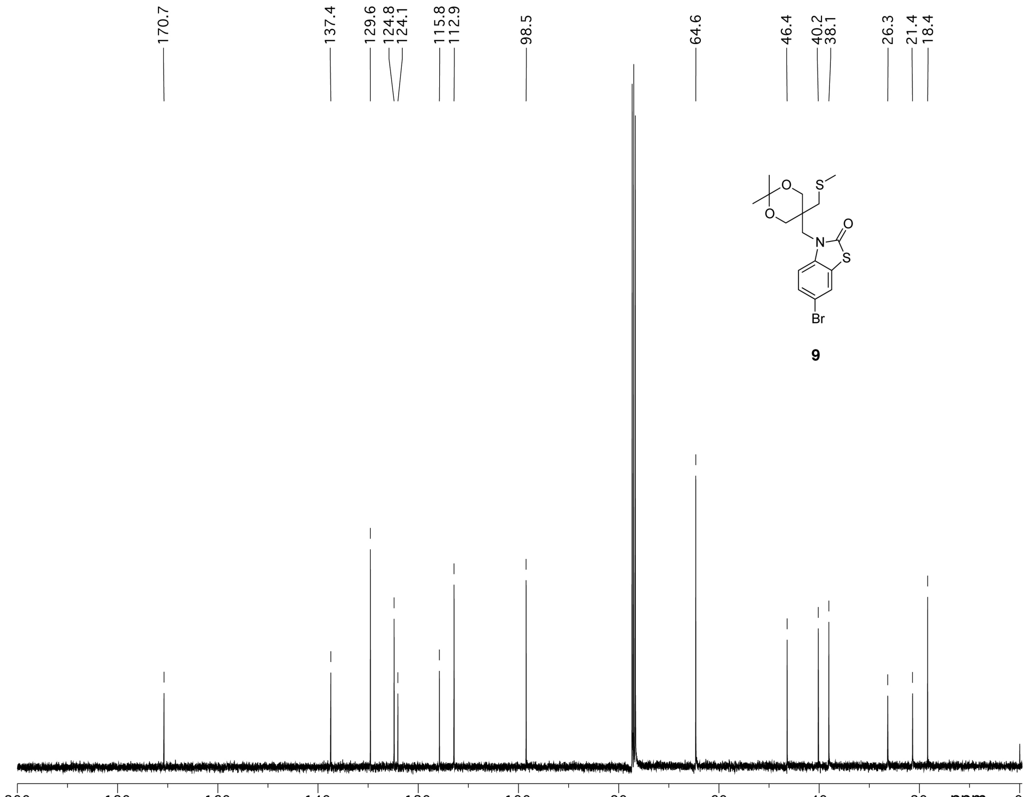

^{1}H-NMR ($CDCl_3$, 400 MHz)

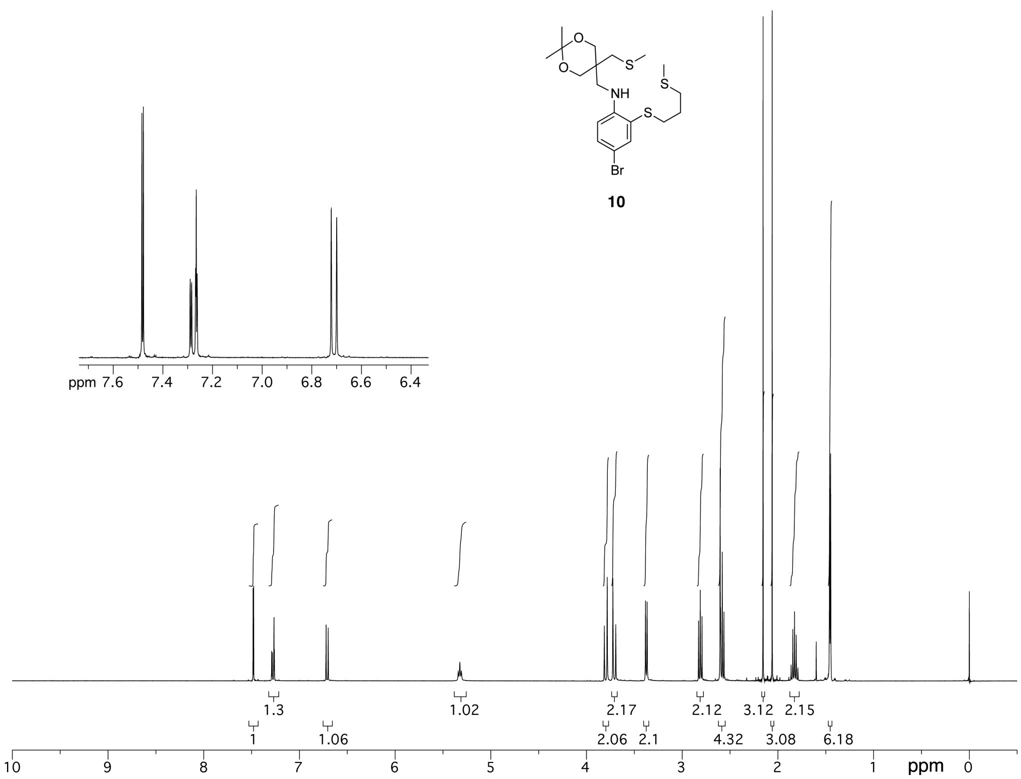

^{13}C-NMR ($CDCl_3$, 100 MHz)

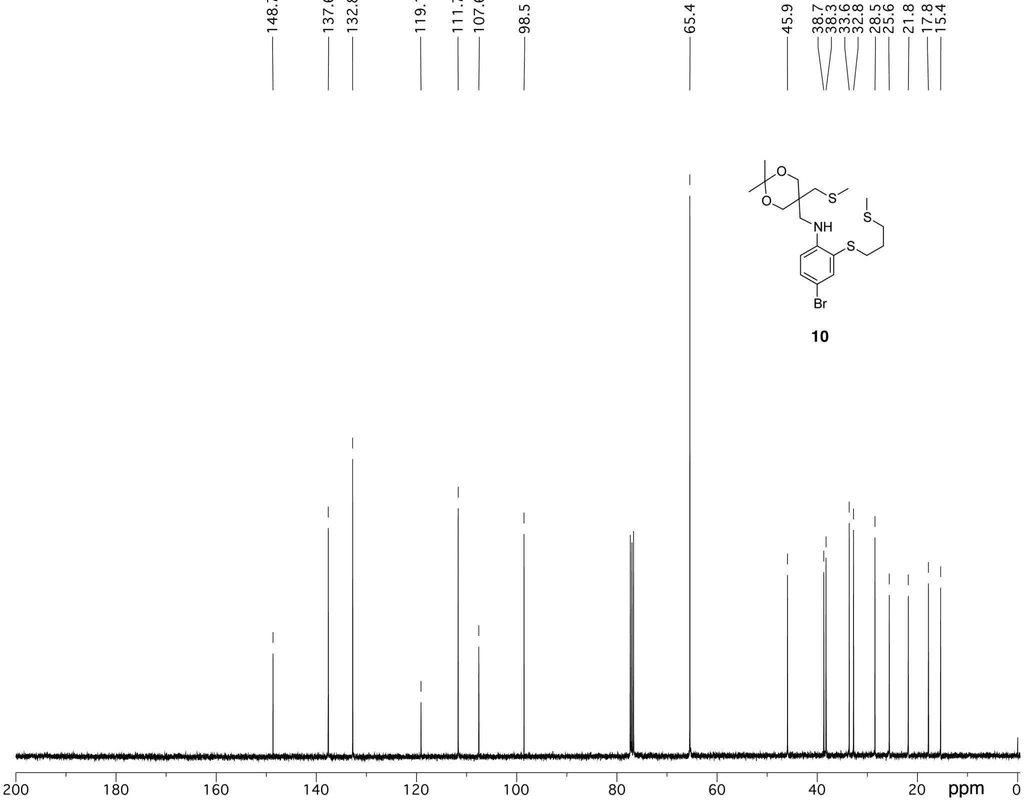

^{1}H-NMR ($CDCl_3$, 400 MHz)

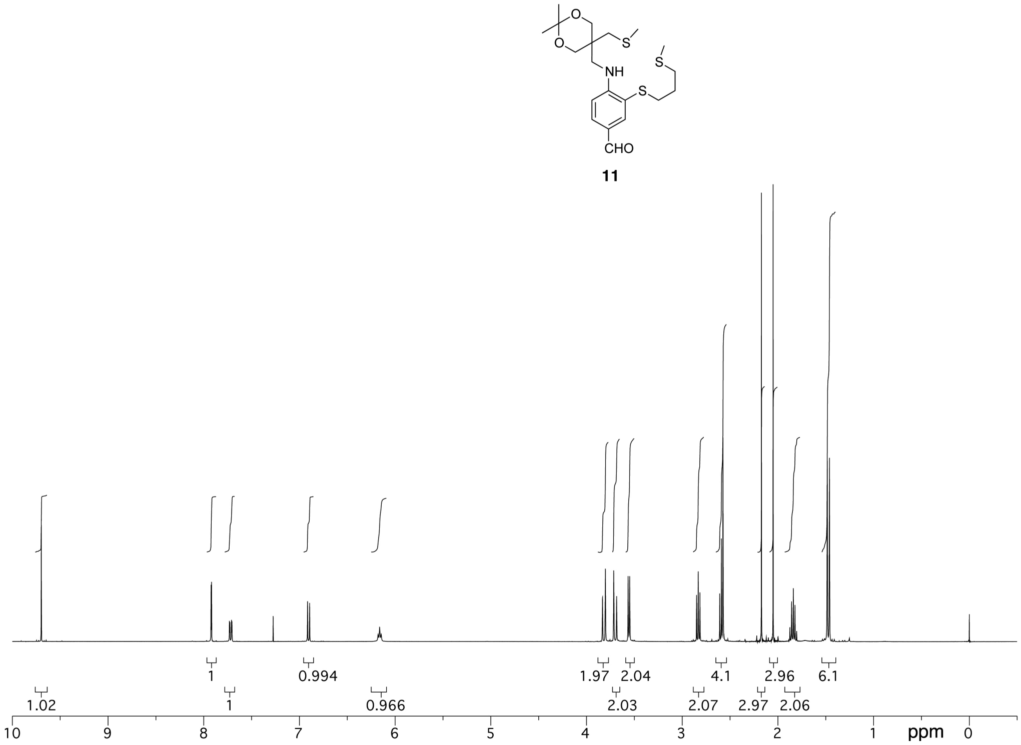

^{13}C-NMR ($CDCl_3$, 100 MHz)

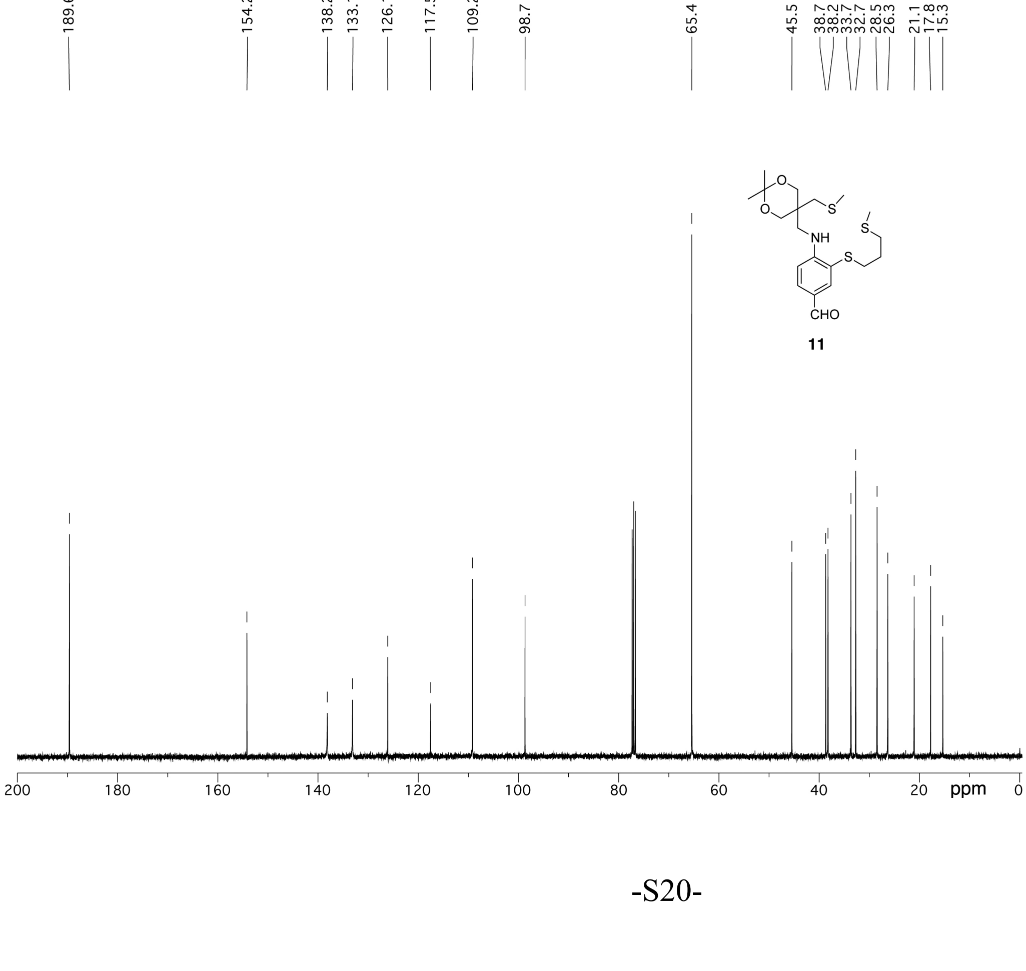

^{1}H-NMR ($CDCl_3$, 400 MHz)

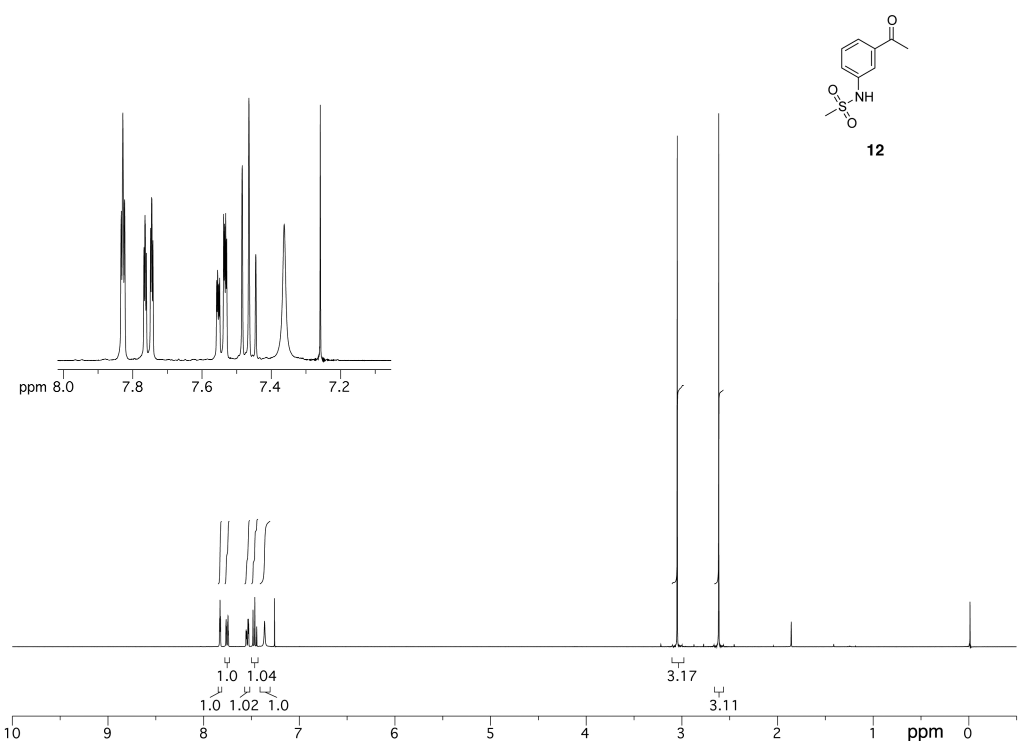

^{13}C-NMR ($CDCl_3$, 100 MHz)

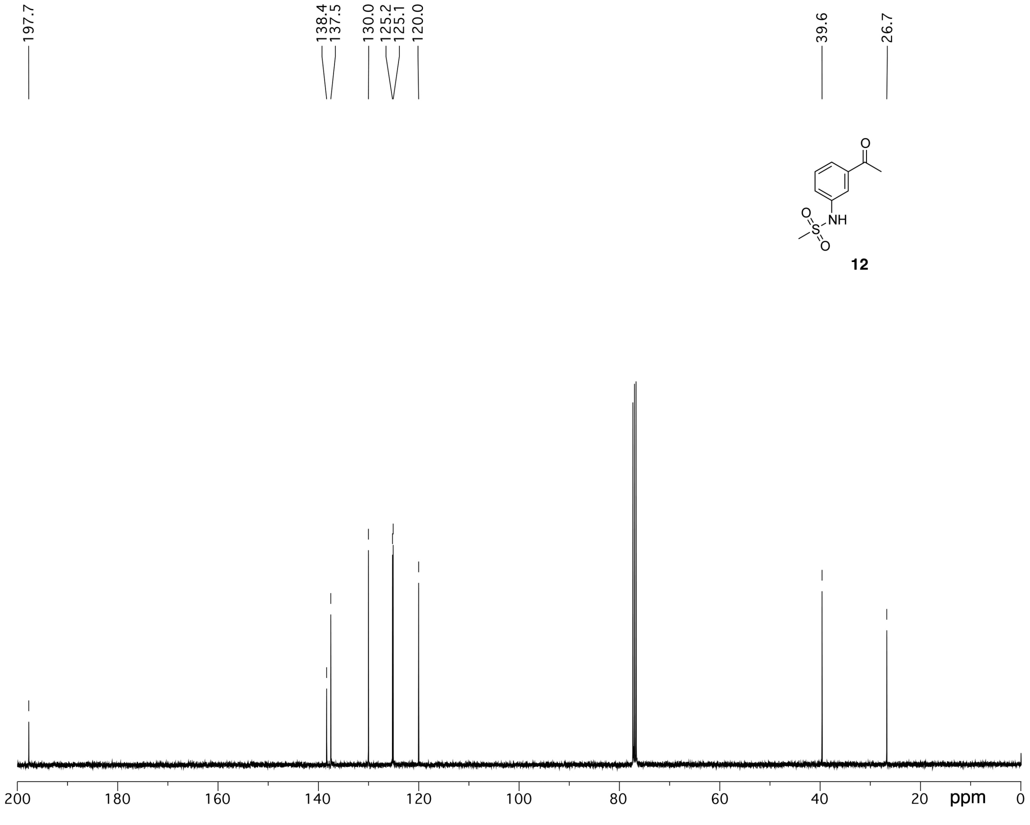

^{1}H-NMR ($CDCl_3$, 400 MHz)

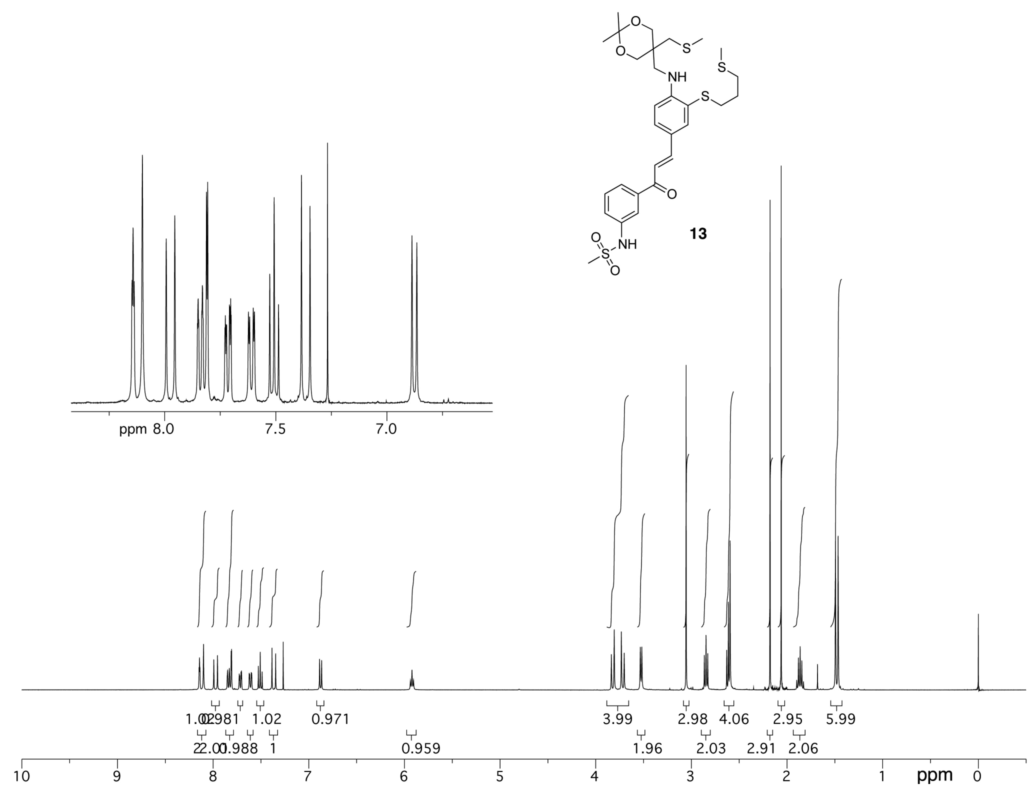

13

^{13}C-NMR ($CDCl_3$, 100 MHz)

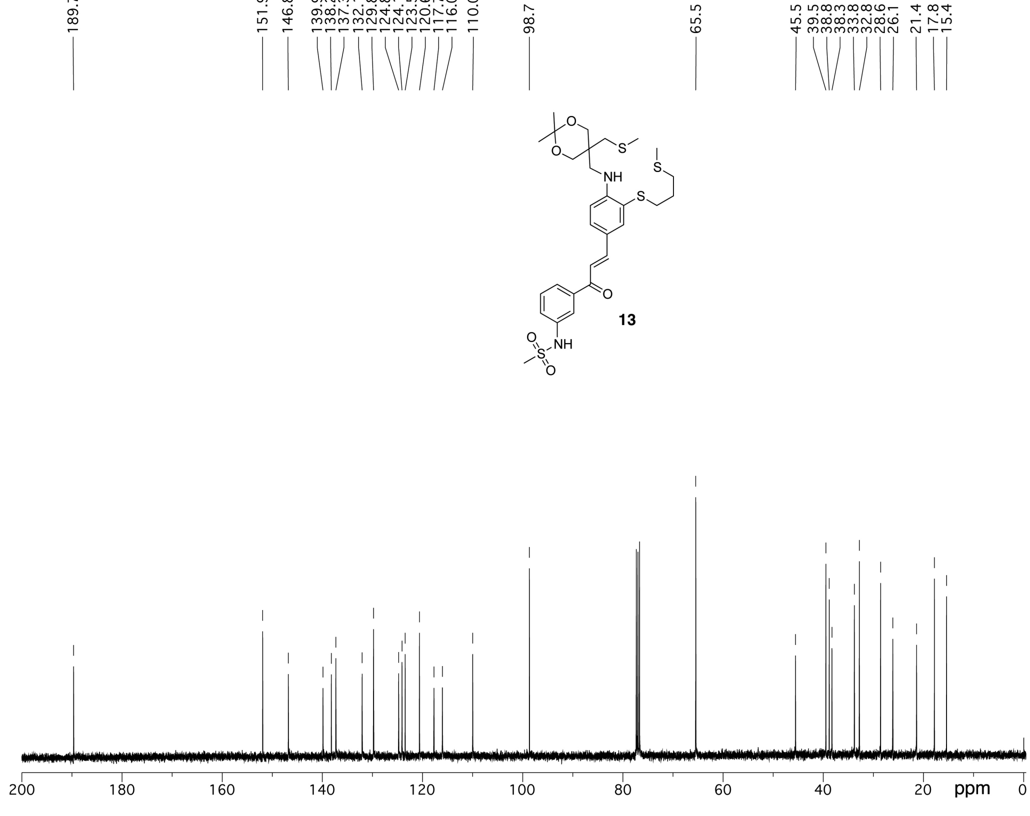

13

^{1}H-NMR (DMSO-d$_6$, 400 MHz)

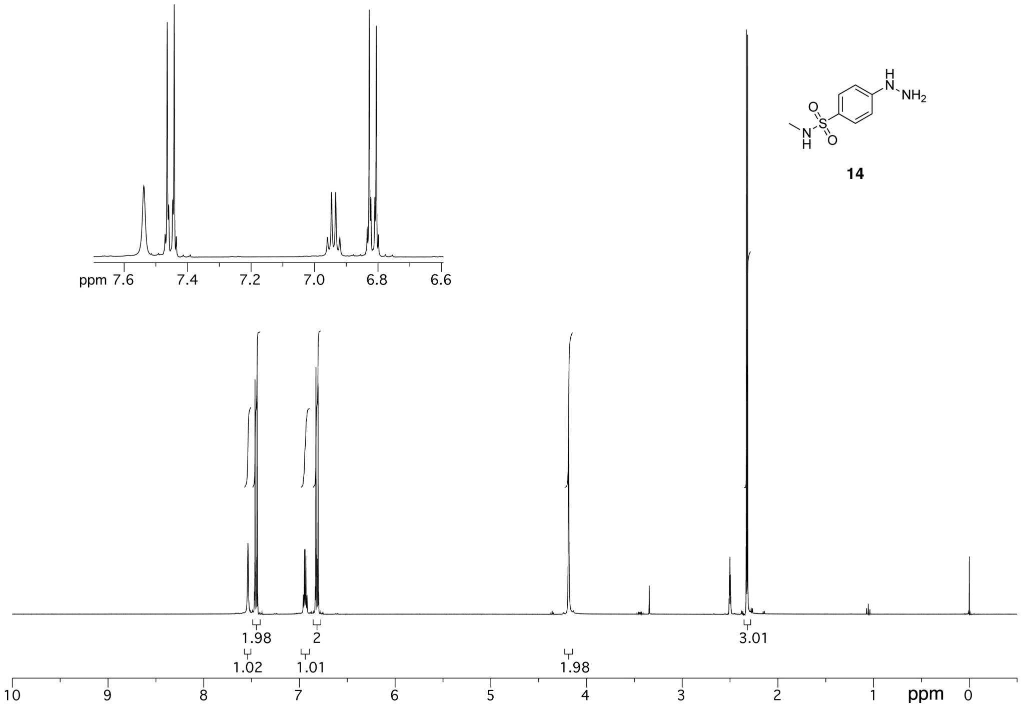

^{13}C-NMR (DMSO-d$_6$, 100 MHz)

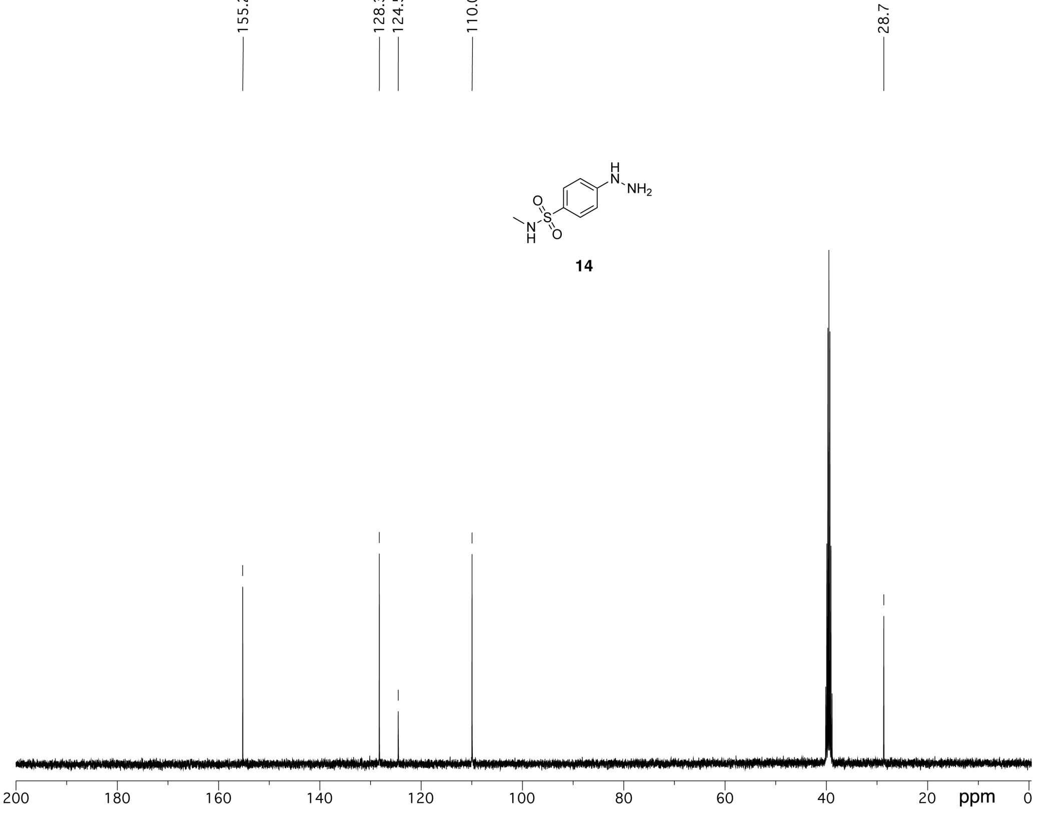

^{1}H-NMR ($CDCl_3$, 400 MHz)

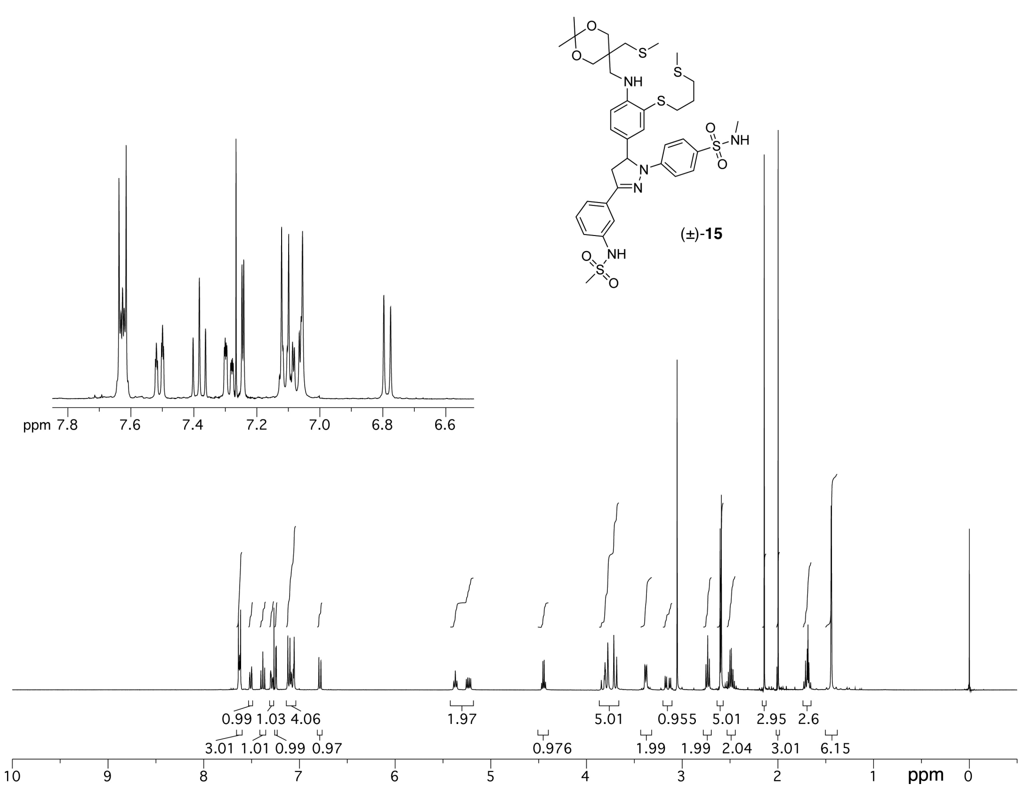

^{13}C-NMR ($CDCl_3$, 100 MHz)

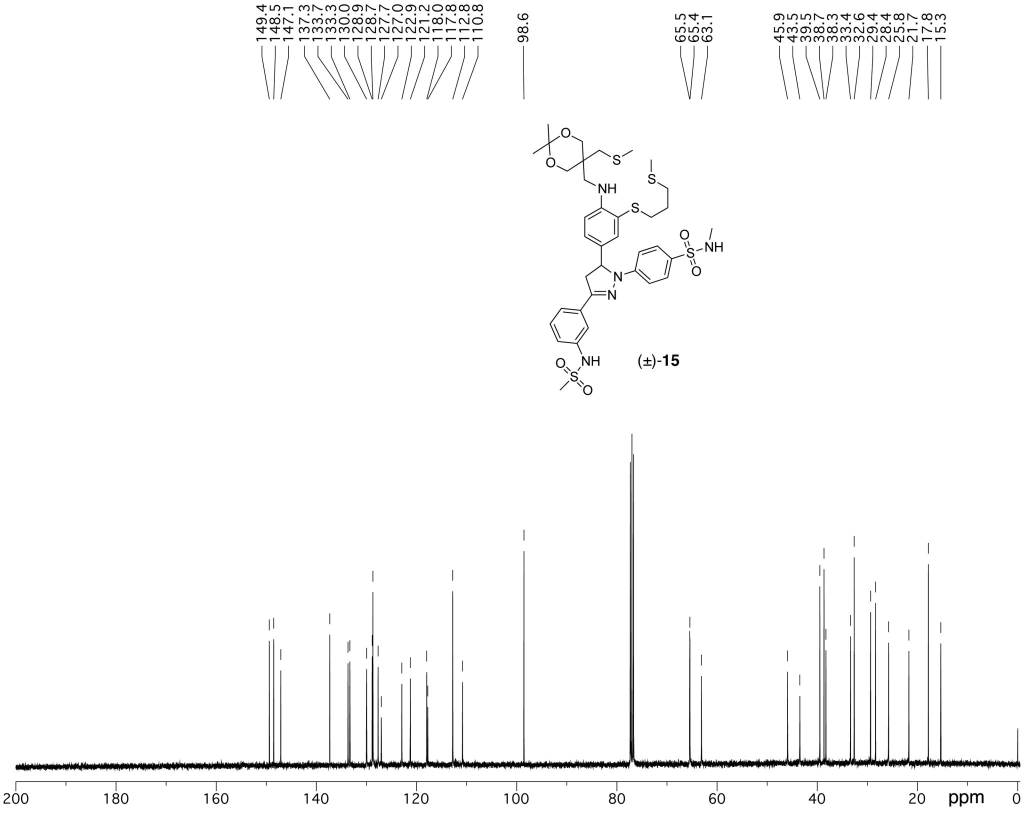

^{1}H-NMR ($CDCl_3$, 400 MHz)

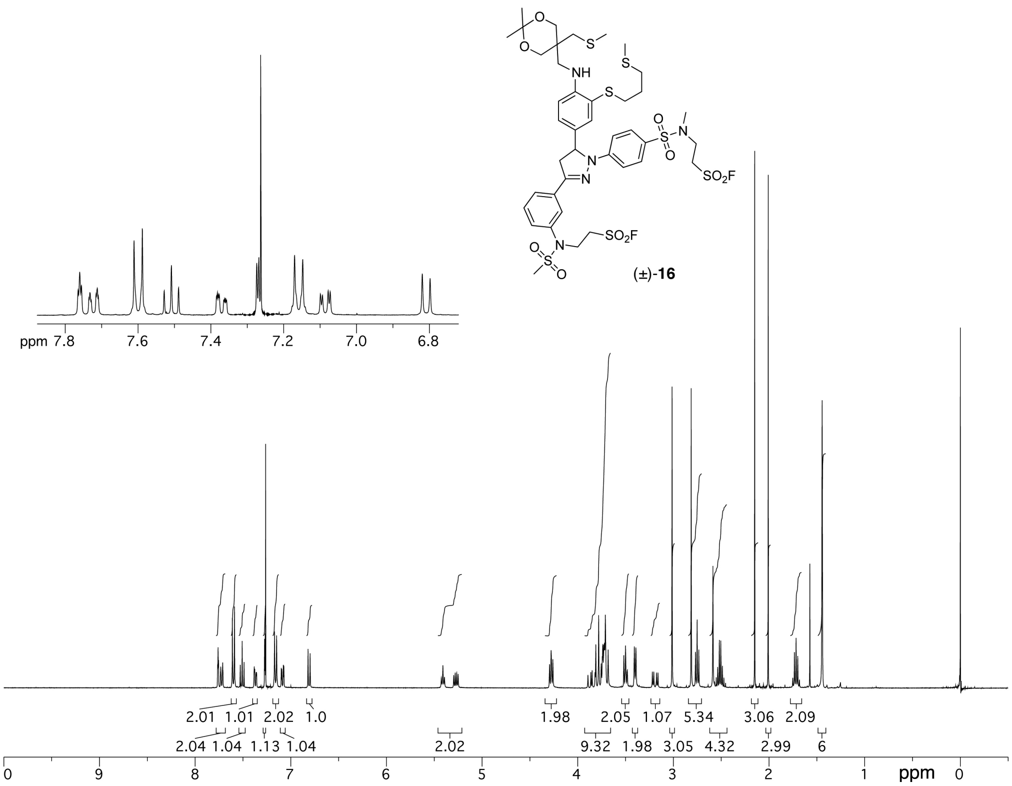

^{19}F-NMR ($CDCl_3$, 376 MHz)

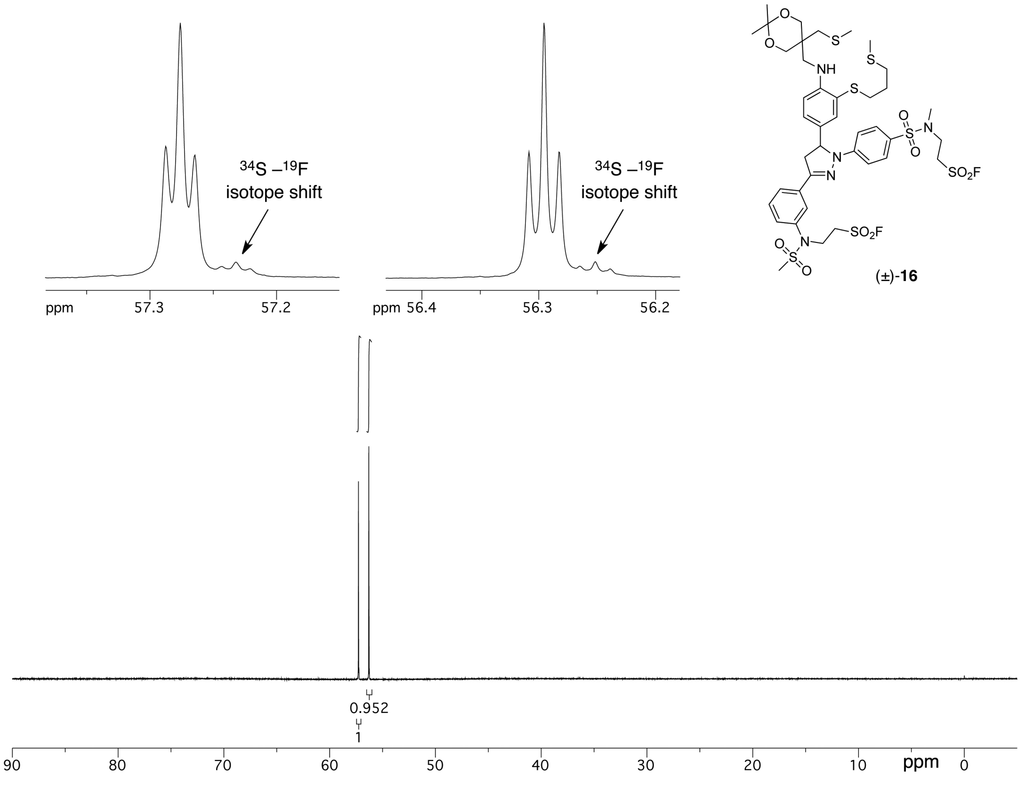

^{1}H-NMR (CDCl$_3$, 400 MHz)

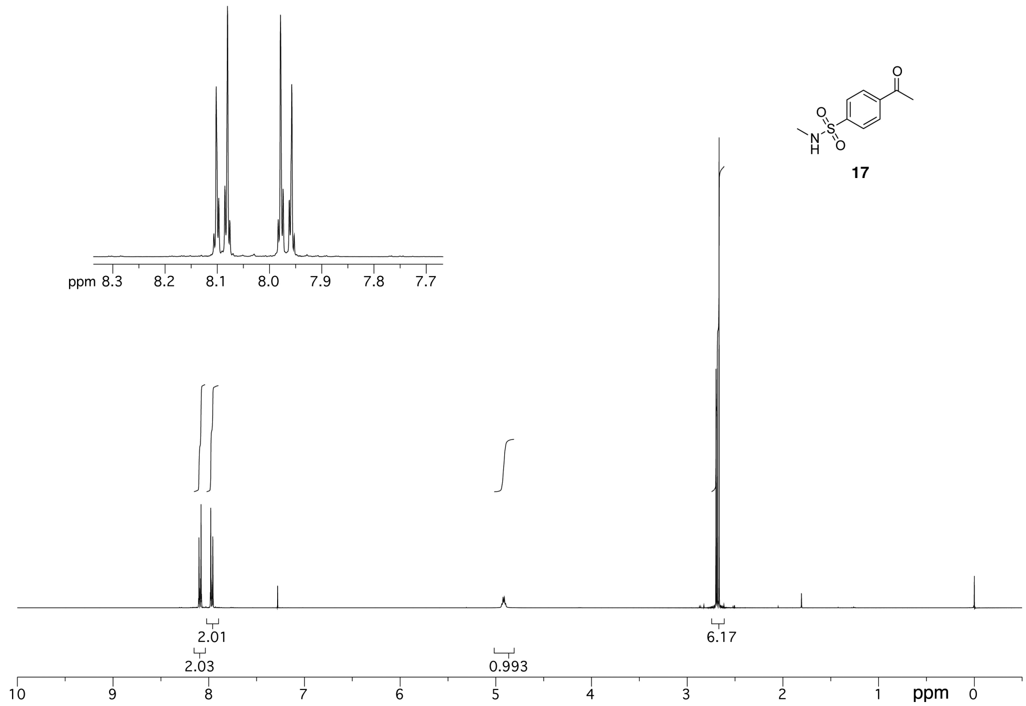

^{13}C-NMR (CDCl$_3$, 100 MHz)

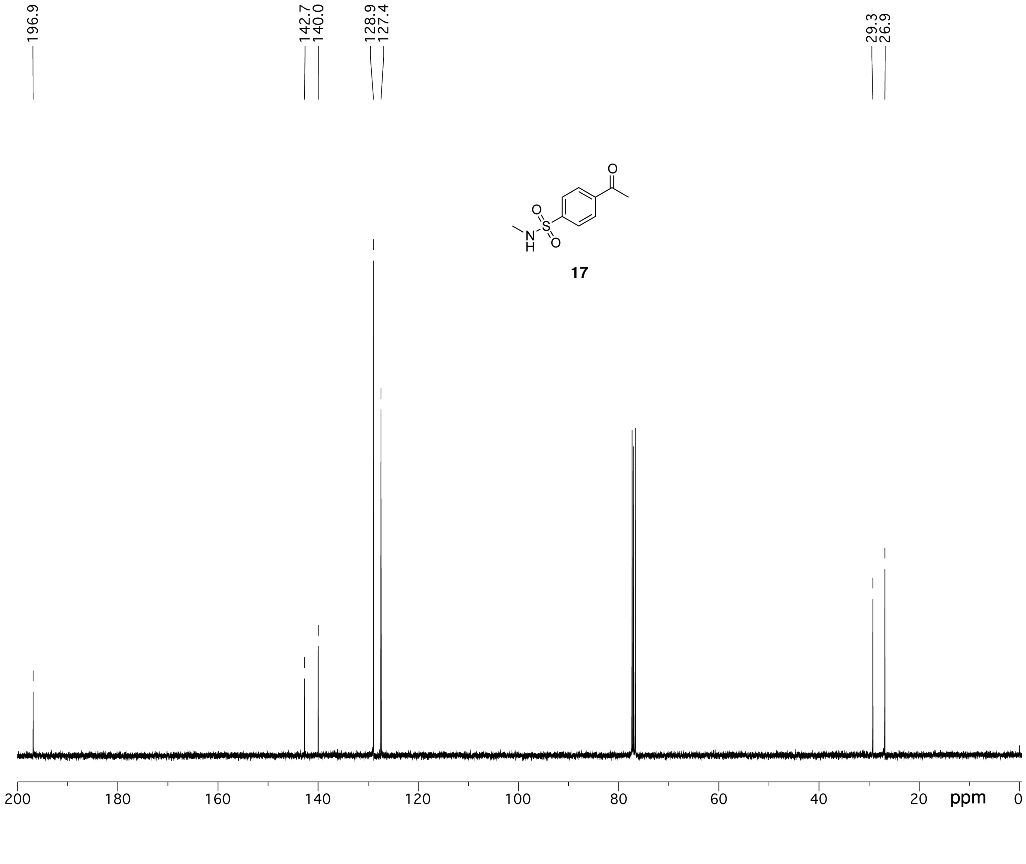

^{1}H-NMR (CDCl$_3$, 400 MHz)

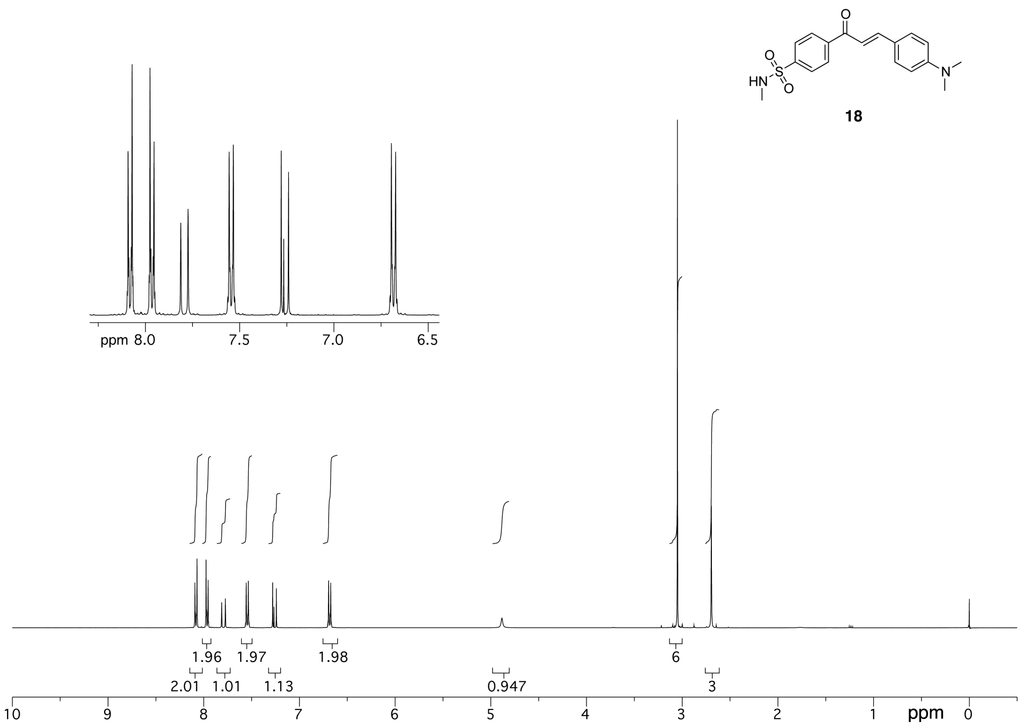

^{13}C-NMR (CDCl$_3$, 100 MHz)

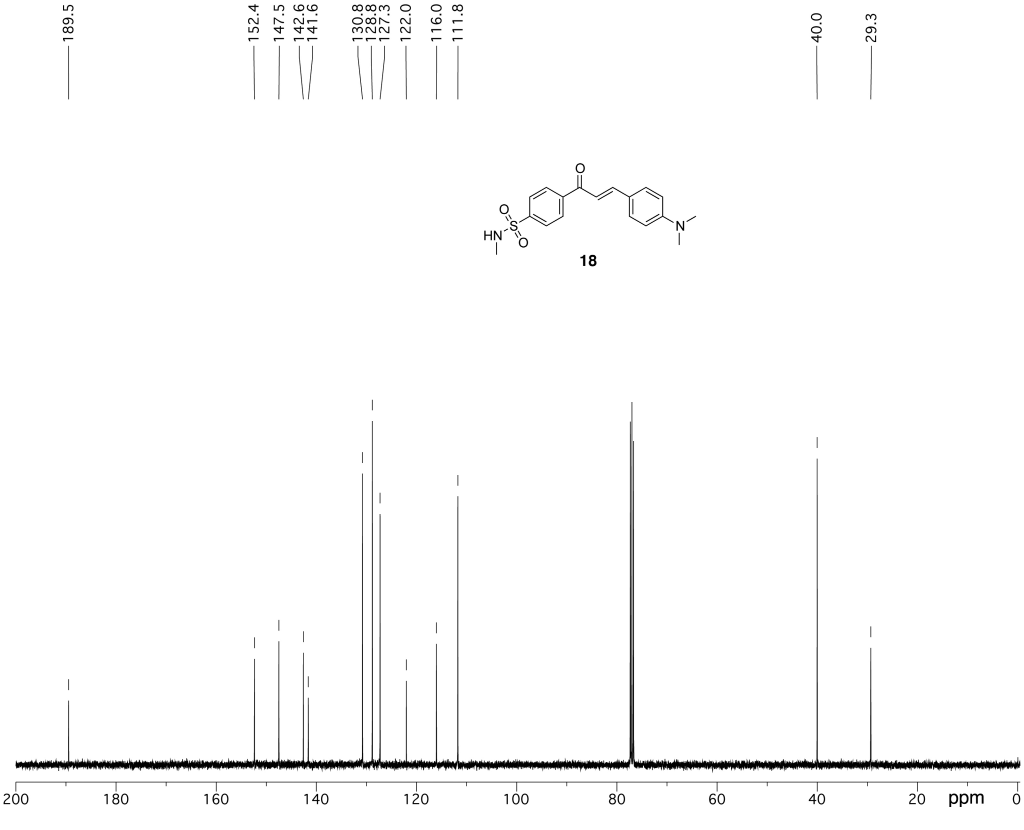

^{1}H-NMR ($CDCl_3$, 400 MHz)

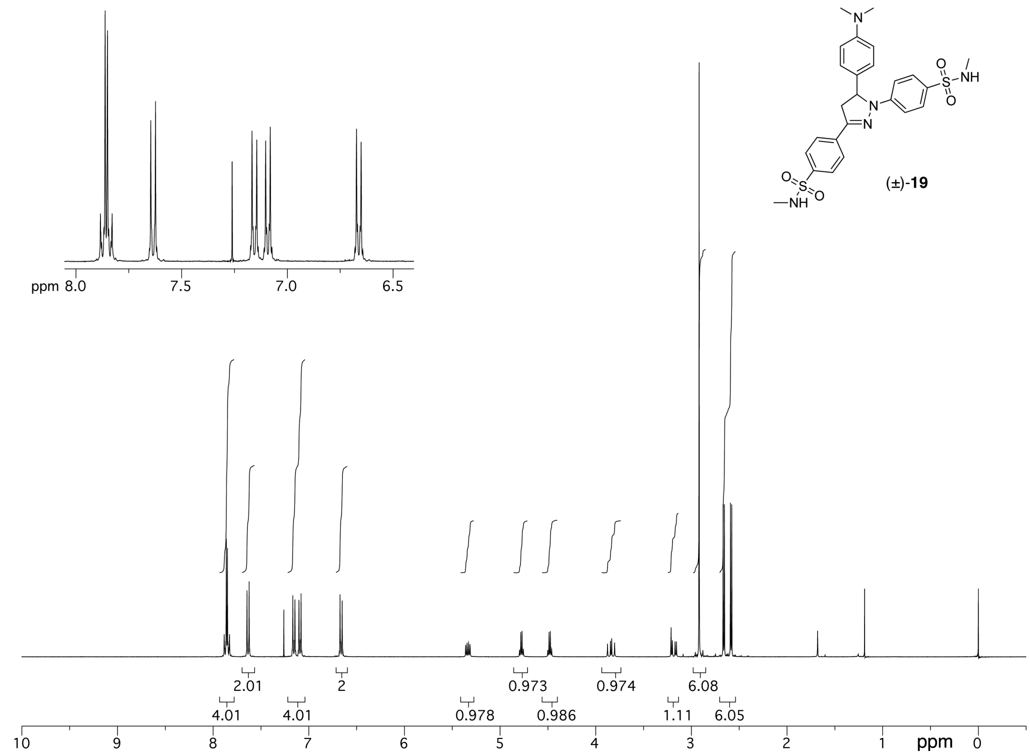

(±)-**19**

^{13}C-NMR ($CDCl_3$, 100 MHz)

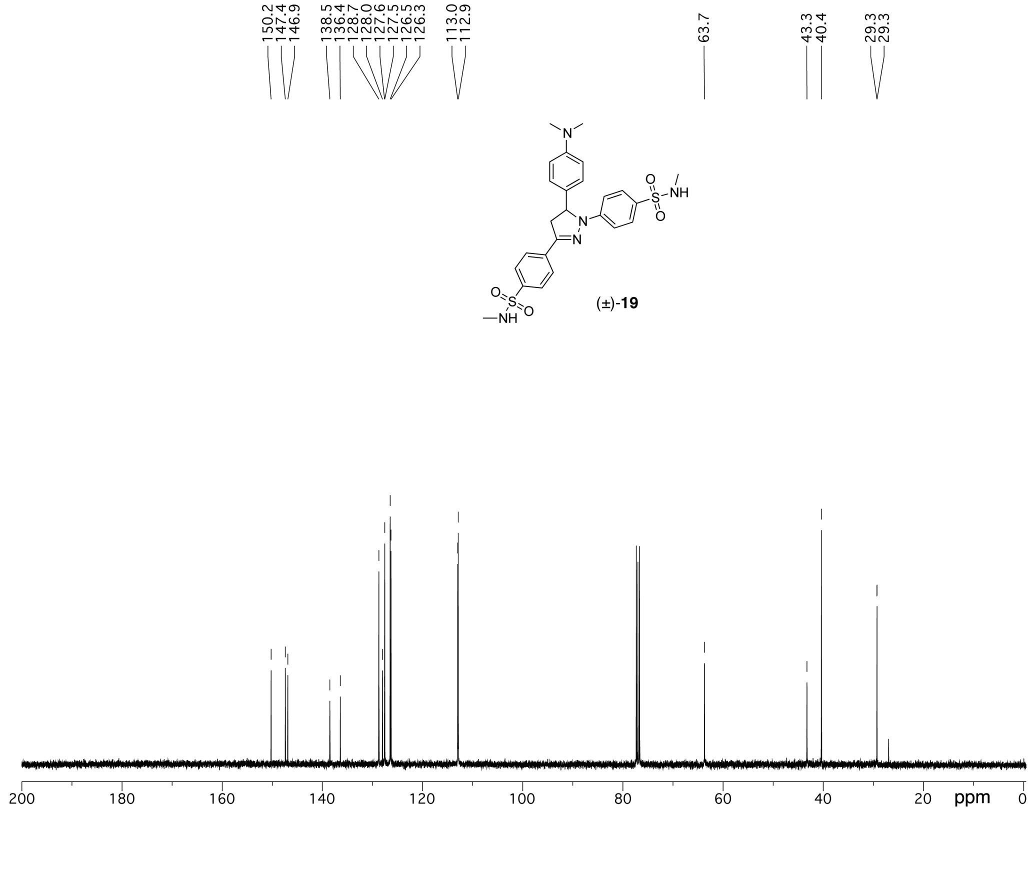

(±)-**19**

^{1}H-NMR ($CDCl_3$, 400 MHz)

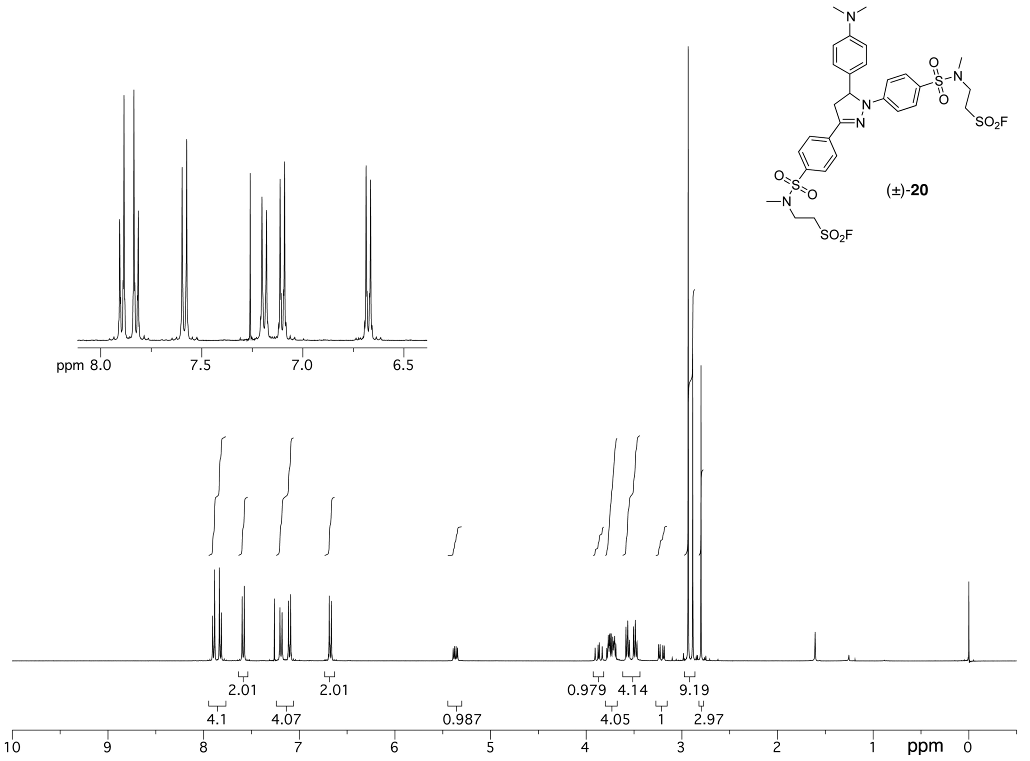

^{19}F-NMR ($CDCl_3$, 376 MHz)

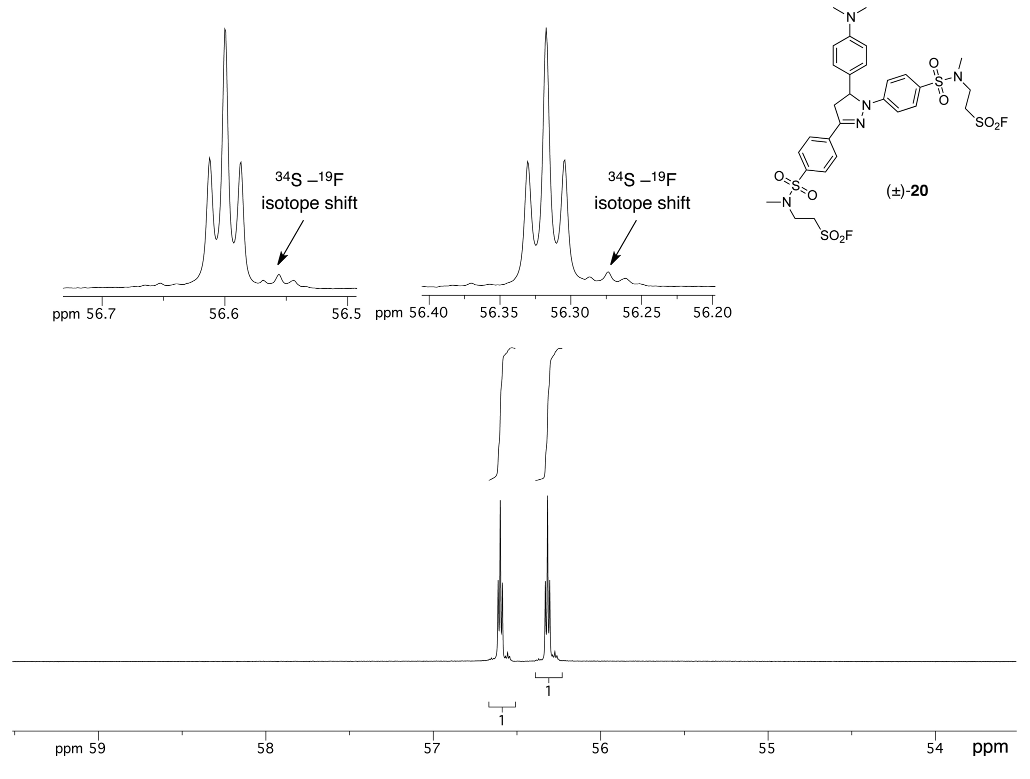

^{1}H-NMR (CDCl$_3$, 400 MHz)

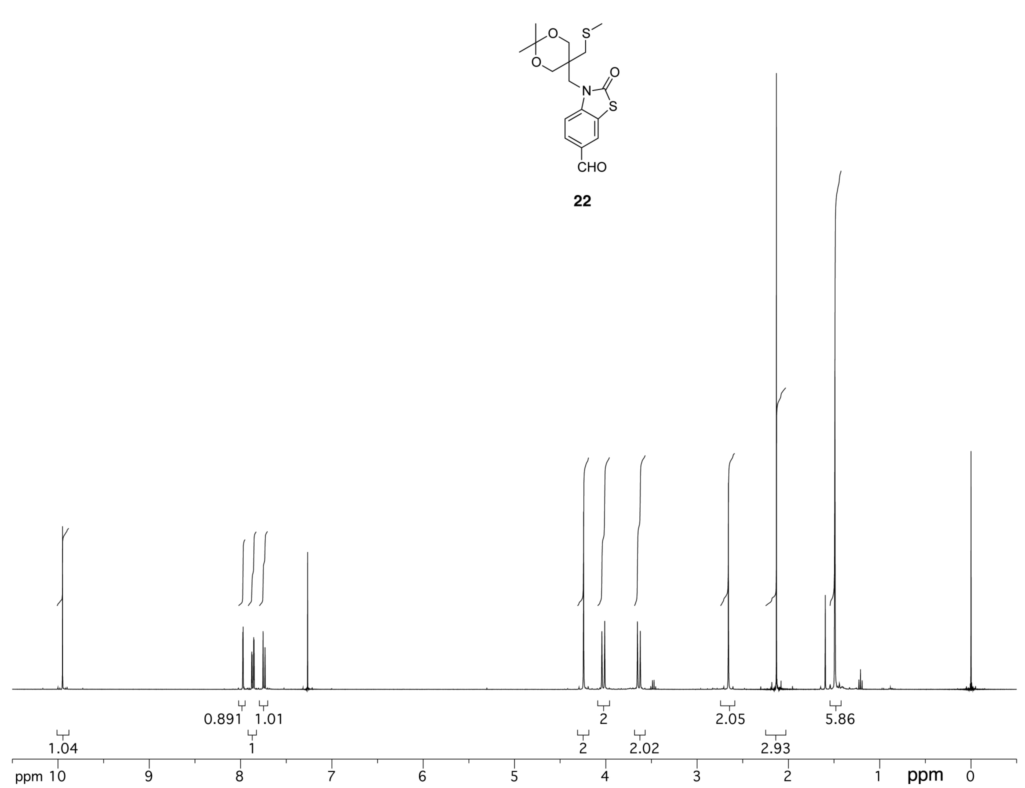

^{13}C-NMR (CDCl$_3$, 100 MHz)

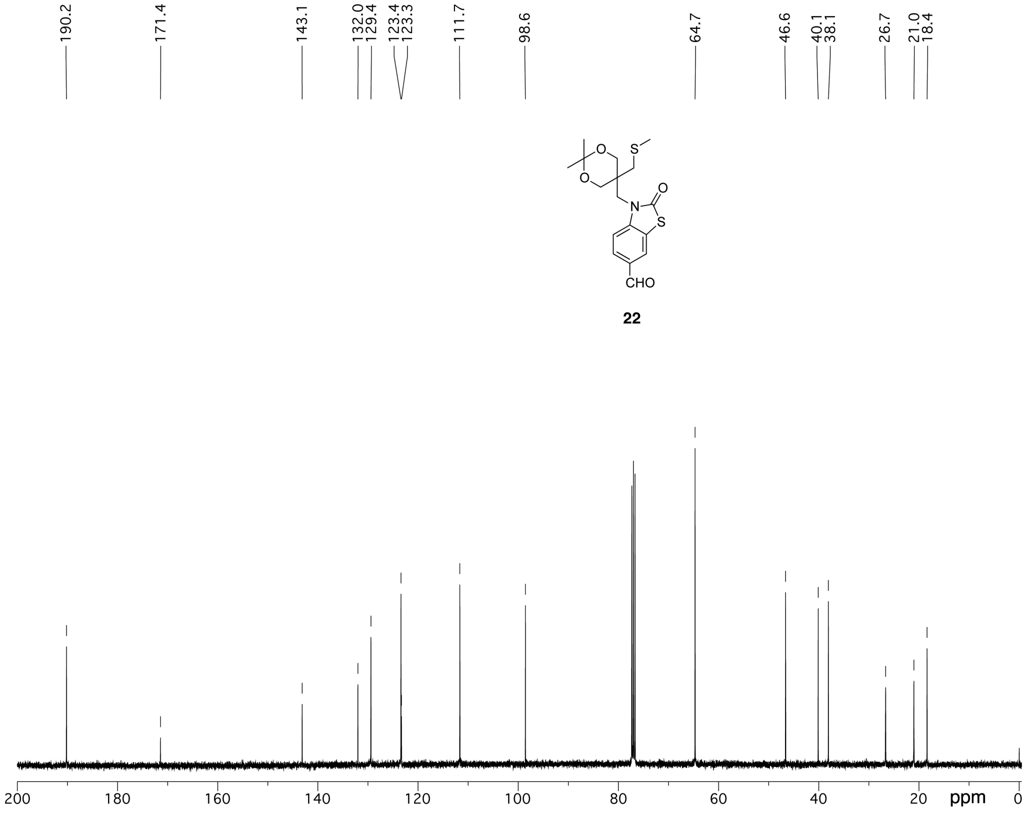

^{1}H-NMR (CDCl$_3$, 400 MHz)

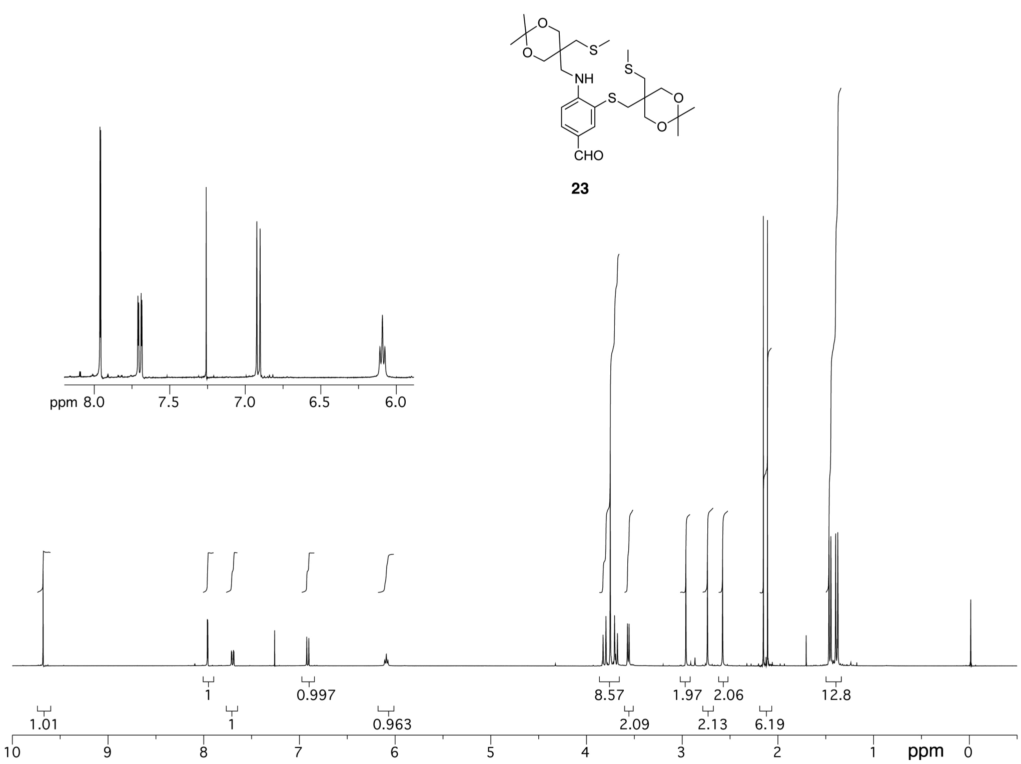

^{13}C-NMR (CDCl$_3$, 100 MHz)

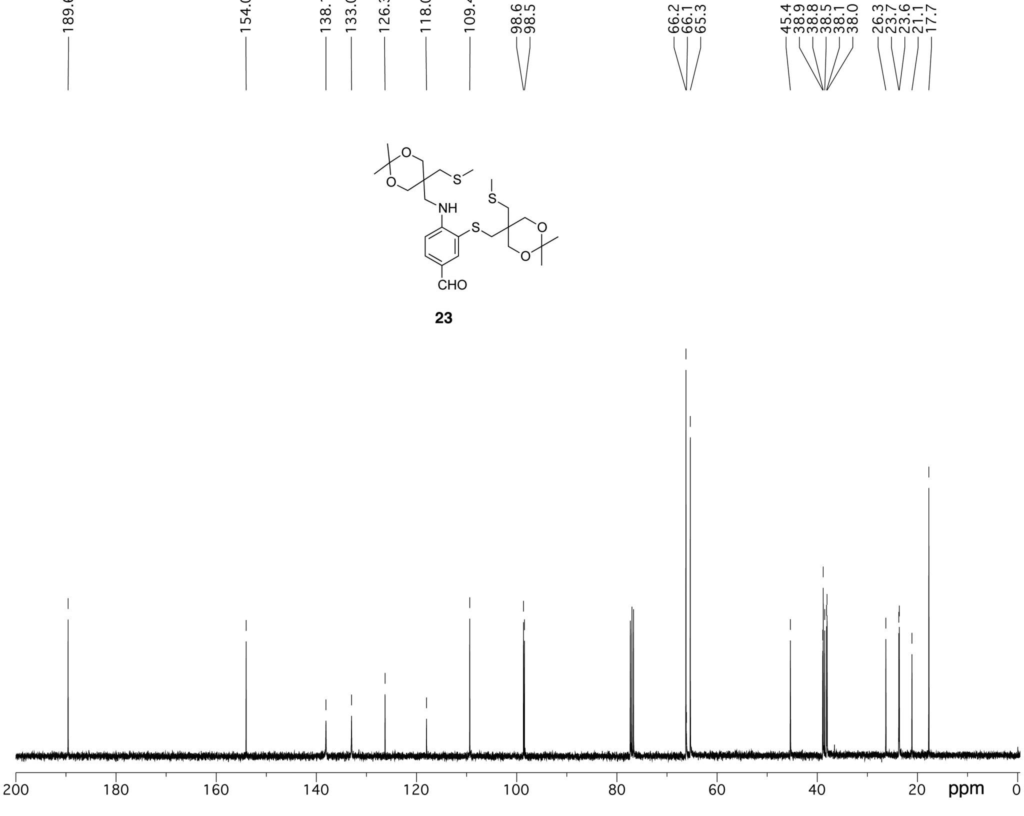

^{1}H-NMR (CDCl$_3$, 400 MHz)

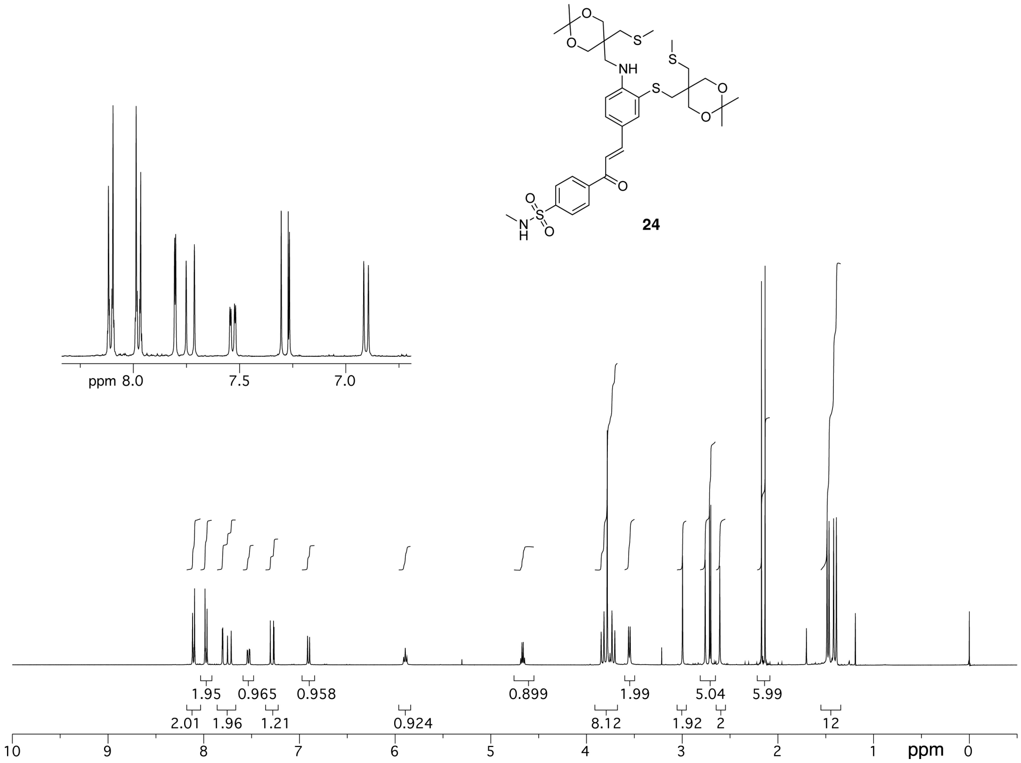

^{13}C-NMR (CDCl$_3$, 100 MHz)

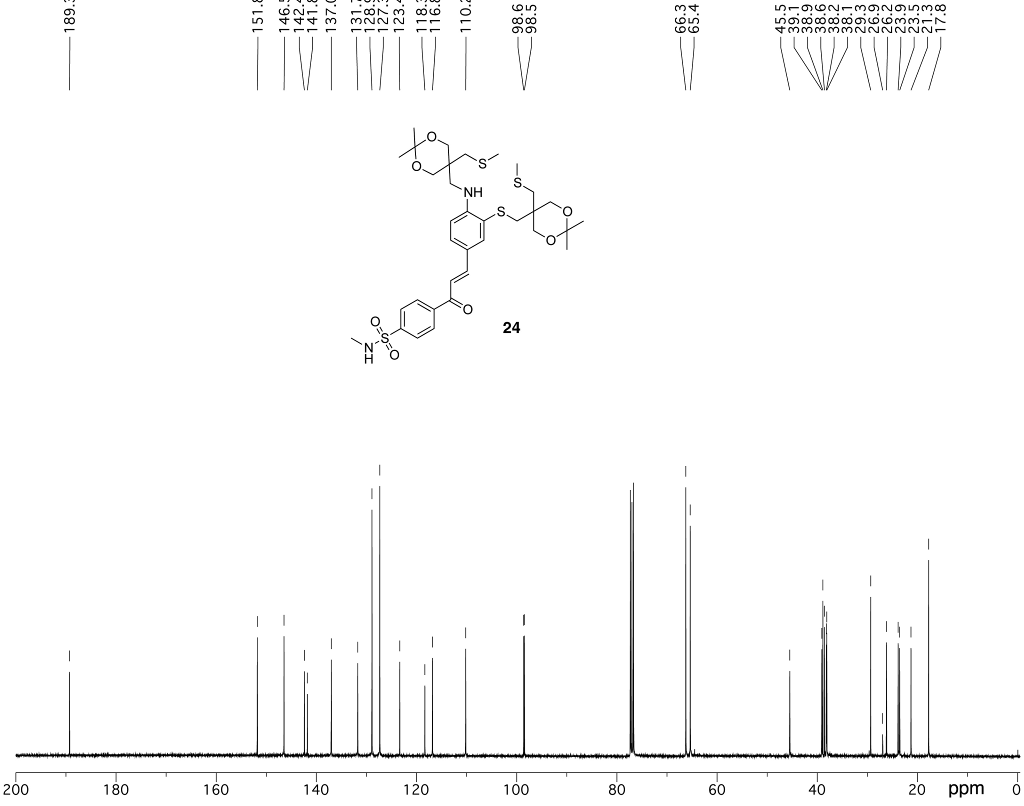

^{1}H-NMR (CDCl$_3$, 400 MHz)

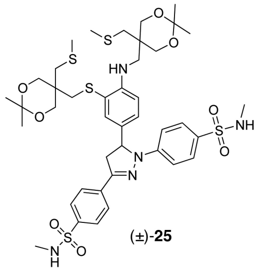

(±)-**25**

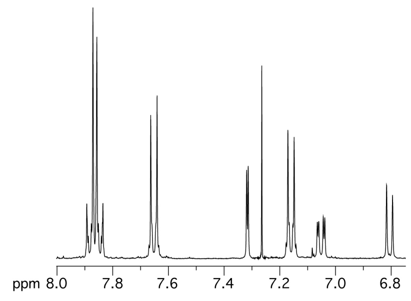

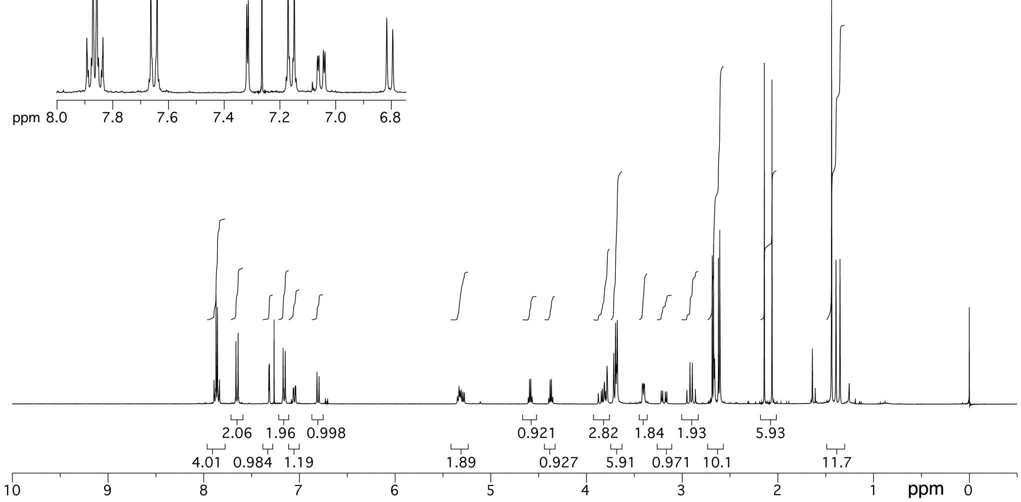

^{13}C-NMR (CDCl$_3$, 100 MHz)

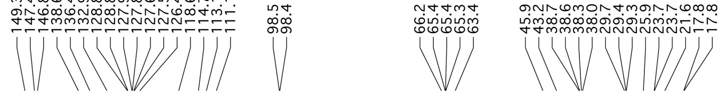

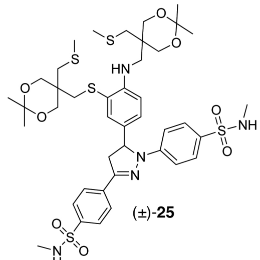

(±)-**25**

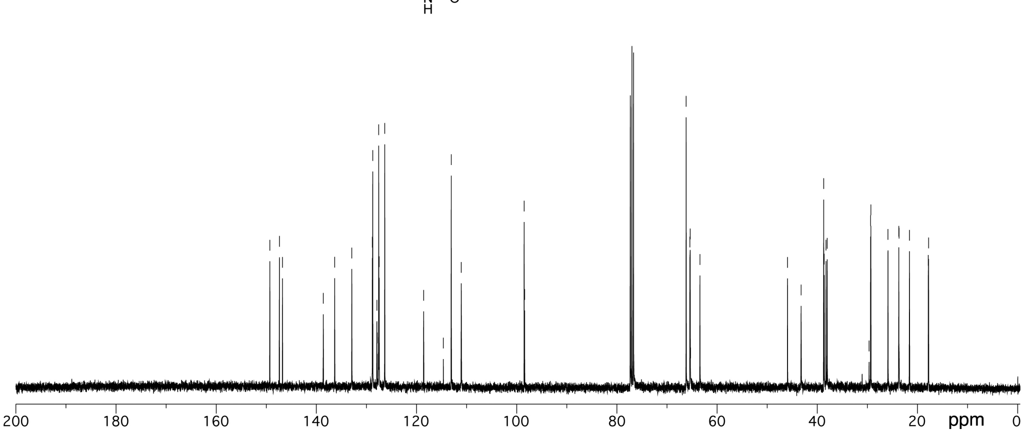

^{1}H-NMR ($CDCl_3$, 400 MHz)

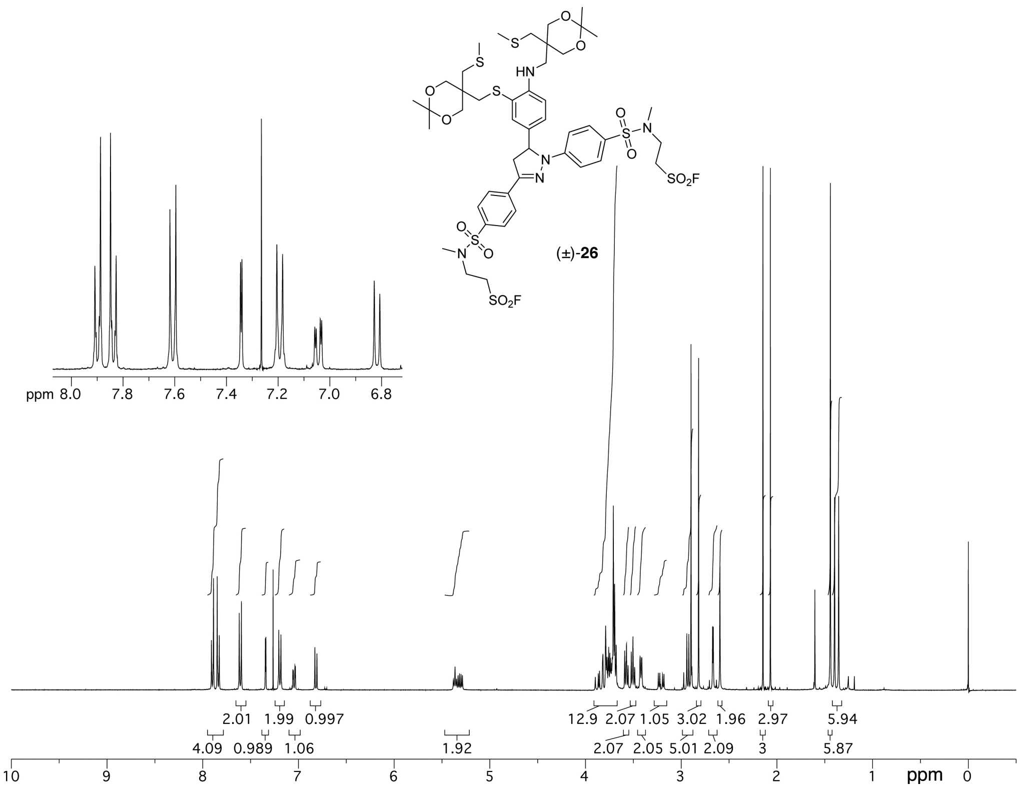

^{19}F-NMR ($CDCl_3$, 376 MHz)

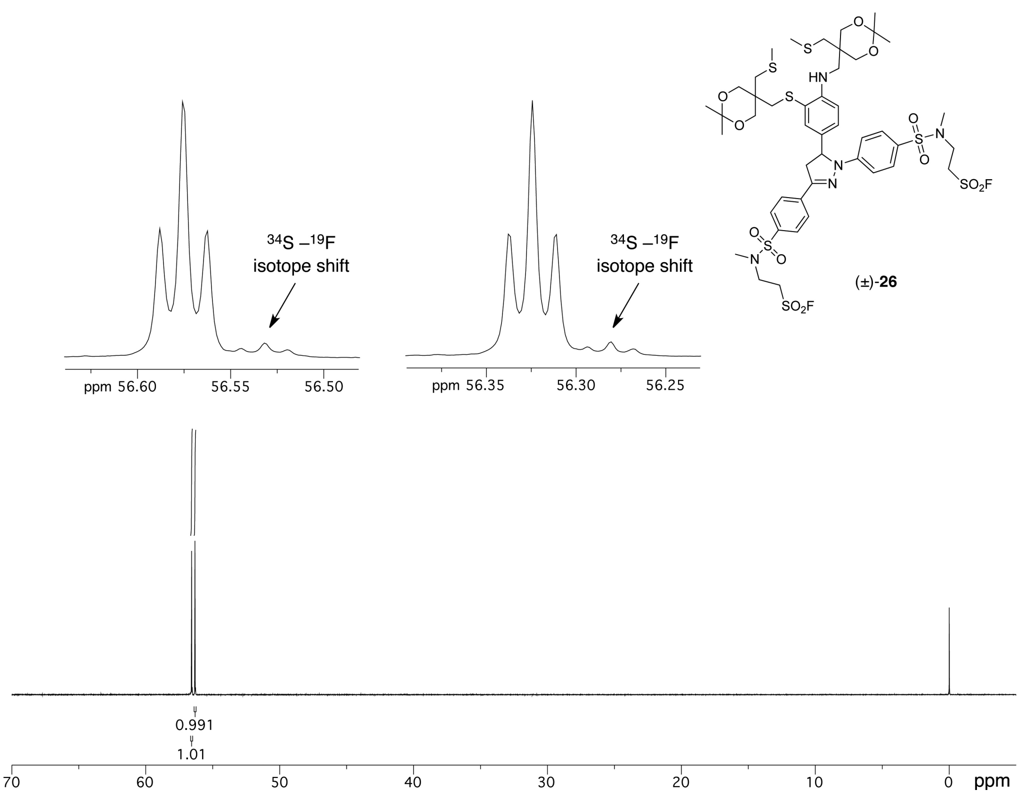

^{1}H-NMR (CDCl$_3$, 400 MHz)

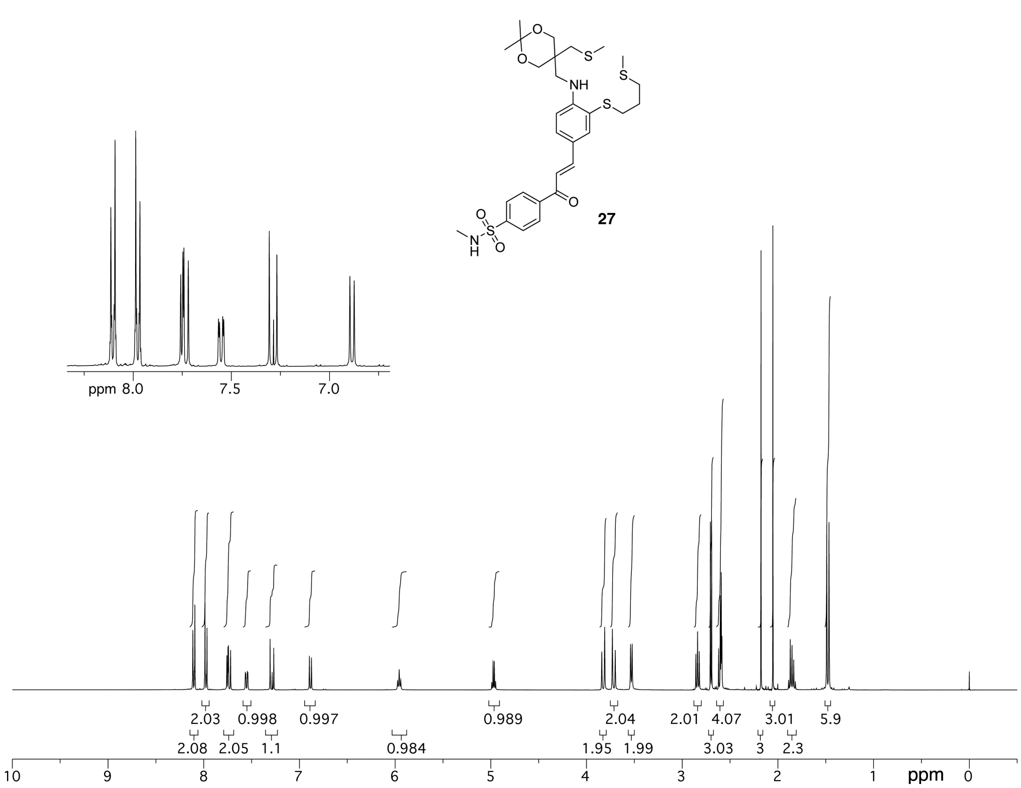

^{13}C-NMR (CDCl$_3$, 100 MHz)

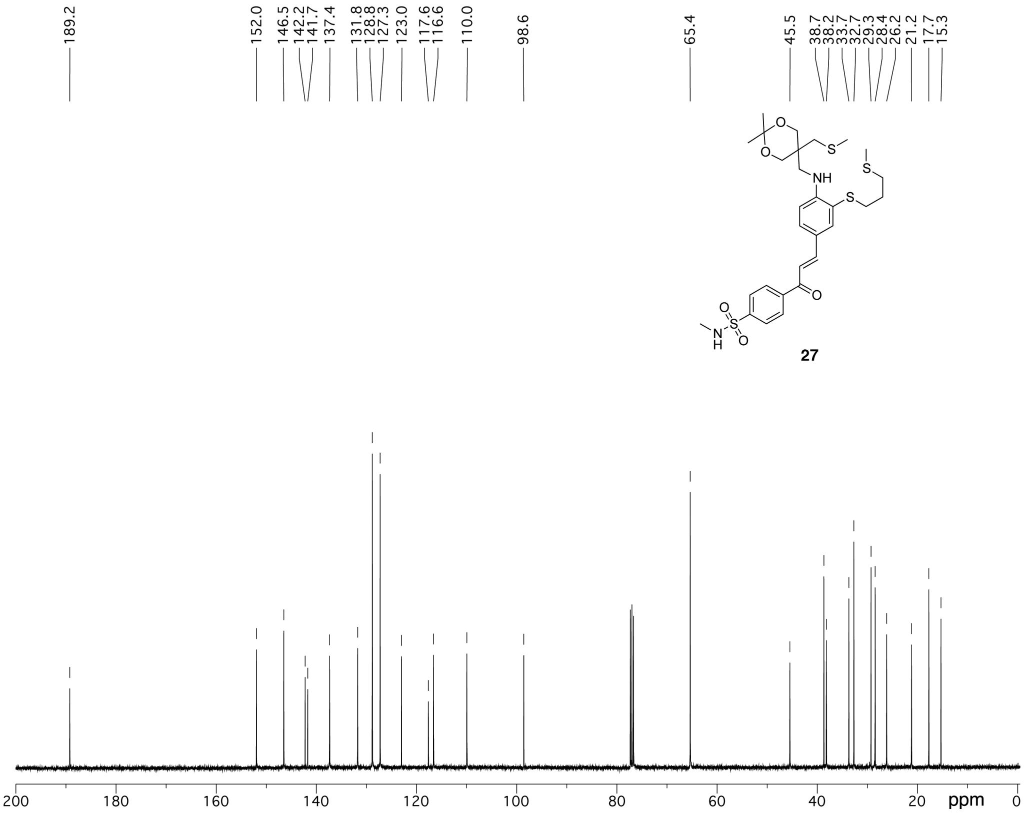

^{1}H-NMR ($CDCl_3$, 400 MHz)

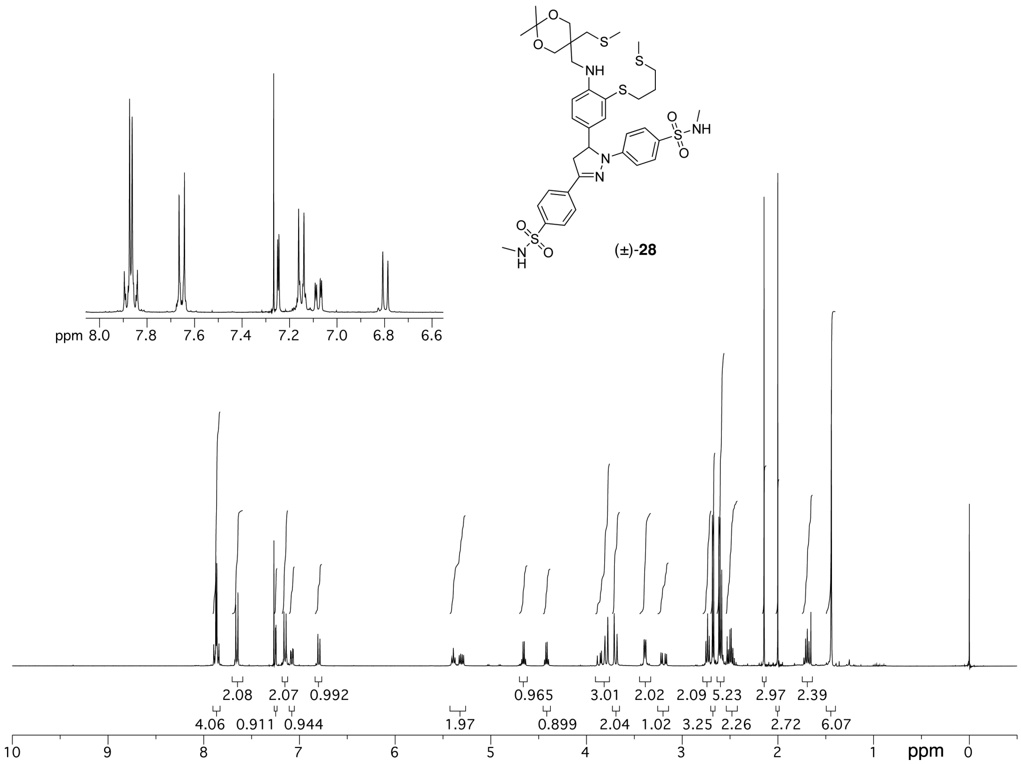

^{13}C-NMR ($CDCl_3$, 100 MHz)

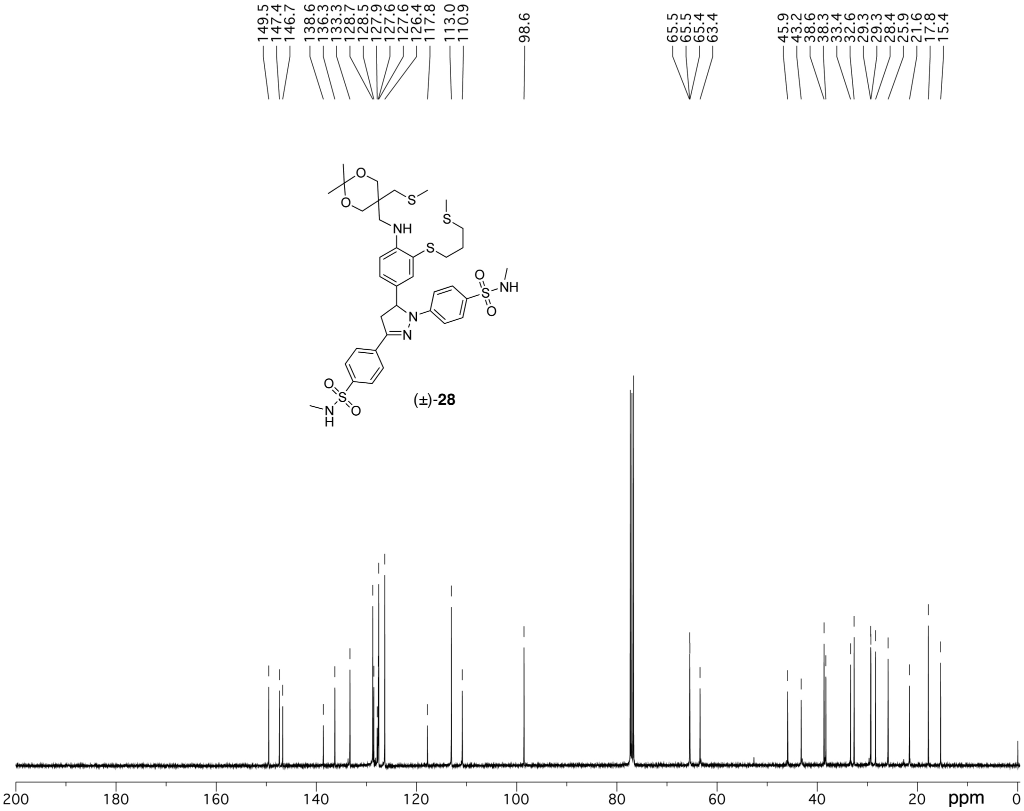

^{1}H-NMR (CDCl$_3$, 400 MHz)

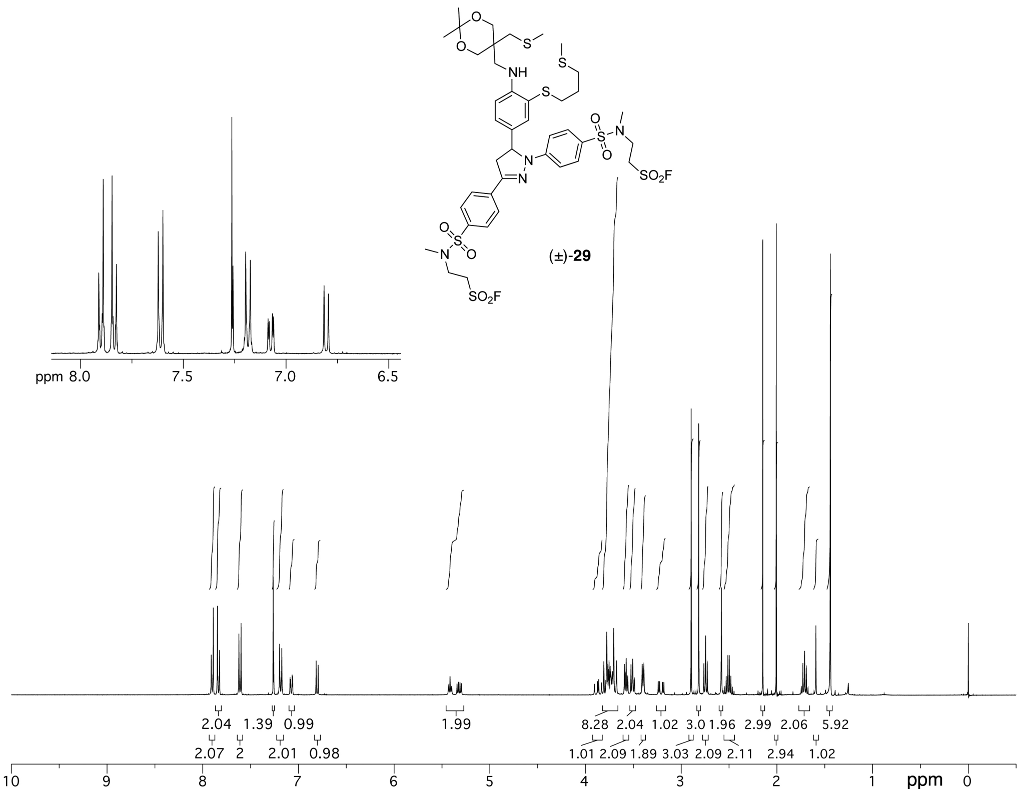

^{19}F-NMR (CDCl$_3$, 376 MHz)

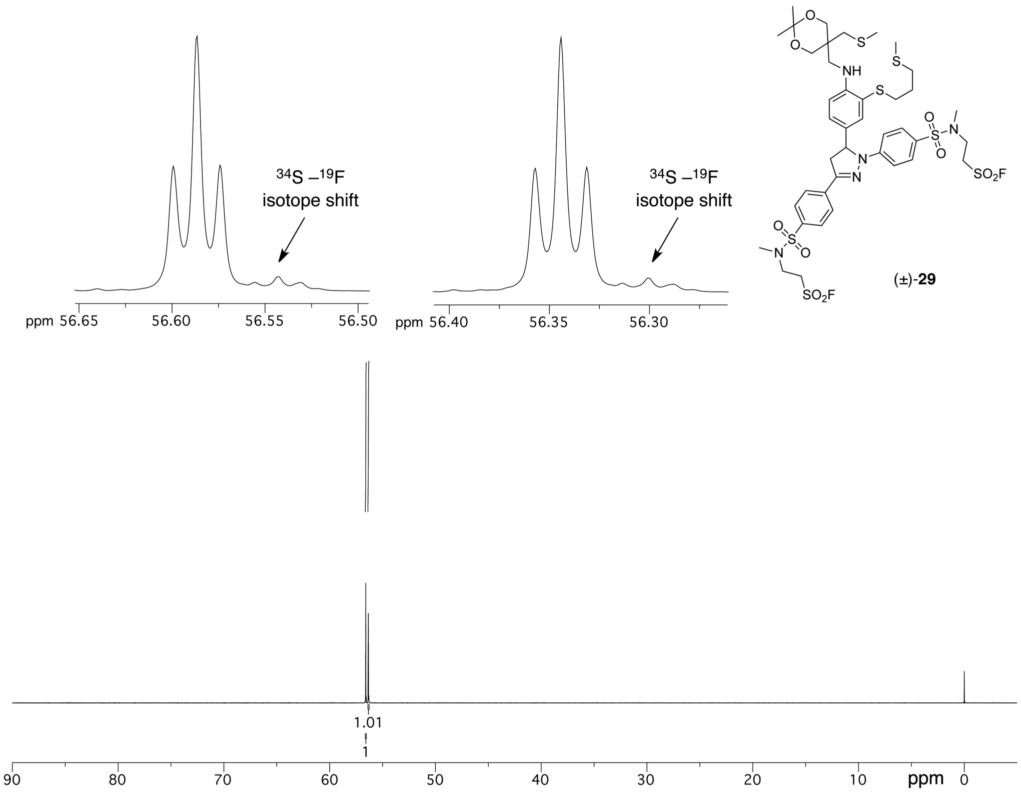

2. Absorption and Fluorescence Spectroscopy

General. All buffers and probe stock solutions were prepared using JT Baker HPLC-grade water or 18.2 MΩ·cm Mili-Q water. Nevertheless, we frequently observed an increased background fluorescence due to traces of adventitious copper. It was therefore necessary to add a small amount of the high-affinity Cu(I) ligand MCL-1[6] as a sequestrant in most experiments as described in detail below. Deoxygenation, where specified, was achieved by bubbling with argon. Initial detection limit experiments indicated that the brass gas regulator could serve as an additional source of copper contamination, which was prevented by passing the argon through a 5 μM filter. UV-vis absorption spectra were acquired at 25°C with a Varian Cary Bio50 spectrophotometer with constant temperature accessory. Fluorescence spectra were recorded with a PTI fluorimeter equipped with a 75 W xenon arc lamp excitation source and model 814 photomultiplier detection system (PMT voltage 1100 V for all measurements). The fluorescence spectra were corrected for the spectral response of the detection system and for the spectral irradiance of the excitation source (via a calibrated photodiode). The path length was 1 cm for absorbance and fluorescence spectra and 10 cm for absorbance measurement for quantum yield determination.

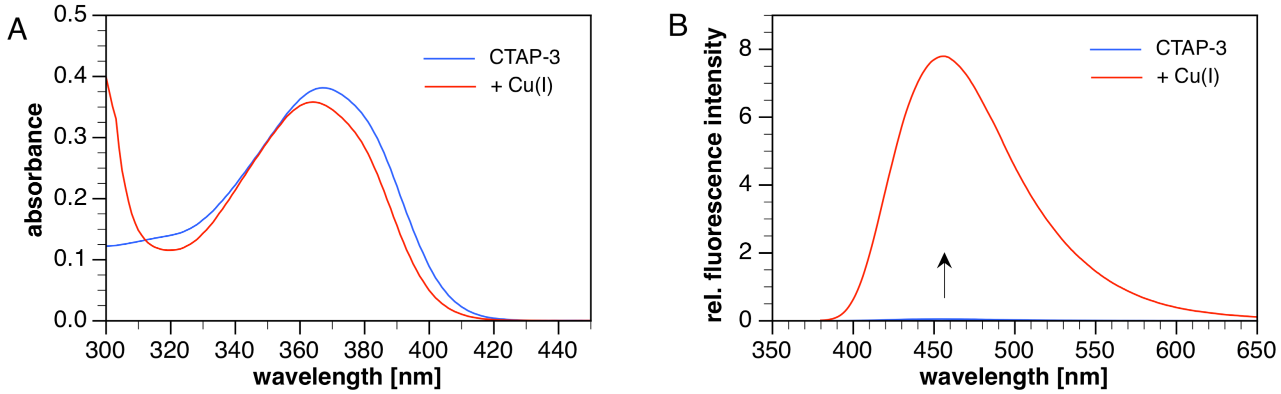

Figure S1: UV-vis absorption (A) and fluorescence emission (B) spectra of CTAP-3 (12.5 μM) in 10 mM MOPS buffer (100 μM sodium ascorbate, 1 μM MCL-1) at pH 7.2 (25°C) before (blue traces) and after (red traces) addition of 15 μM Cu(I), generated by in situ reduction of Cu(II)SO_4 with sodium ascorbate.

Fluorescence Enhancement Factors and Quantum Yields. Fluorescence enhancement factors were determined at 1-5 μM probe concentration in pH 7.2 MOPS buffer containing 1 μM MCL-1[6] as a sequestrant for traces of background copper and 100 μM sodium ascorbate as a reducing agent. Excitation was provided at 365 nm for CTAP-3 and at 380 nm for probes **4** and **5**. Emission spectra were recorded before and after saturation with Cu(I) generated by in situ reduction of $CuSO_4$ with ascorbate. After background subtraction, spectra were integrated over the range of $\lambda_{max} \pm 10$ nm, and the ratio of the integrated intensities before and after Cu(I) saturation were taken as the enhancement factor. There was no observable difference in emission maximum between the free and Cu(I)-saturated form of each probe. In each case, complete reversal of the fluorescence response to Cu(I) was observed upon addition of excess MCL-1. Fluorescence quantum yields were determined using norharmane in 0.1 N H_2SO_4 (Φ_f 0.58)[7] as standard. During the optimization process, the quantum yields of the Cu(I)-saturated probes were initially determined via single-point measurement at OD 0.1, and that of CTAP-3 was subsequently verified by a four point measurement over the OD range of 0.1-0.4 (10 cm path length).

Time-resolved Fluorescence Spectroscopy. Fluorescence decay profiles were acquired at the respective emission maximum of each fluorophore using a single photon counting spectrometer (Edinburgh Instruments, LifeSpec Series) equipped with a pulsed laser diode as the excitation source (372 nm, FWHM = 80 ps, 10 MHz repetition rate, 1024 channel resolution). Sample solutions of the Cu(I)-saturated probes were prepared via in situ reduction of $CuSO_4$ with ascorbate in deoxygenated pH 7.2 MOPS-K^+ buffer (10 mM, 25°C). The time decay data were analyzed by non-linear least squares fitting with deconvolution of the instrumental response function using the FluoFit software package.[8]

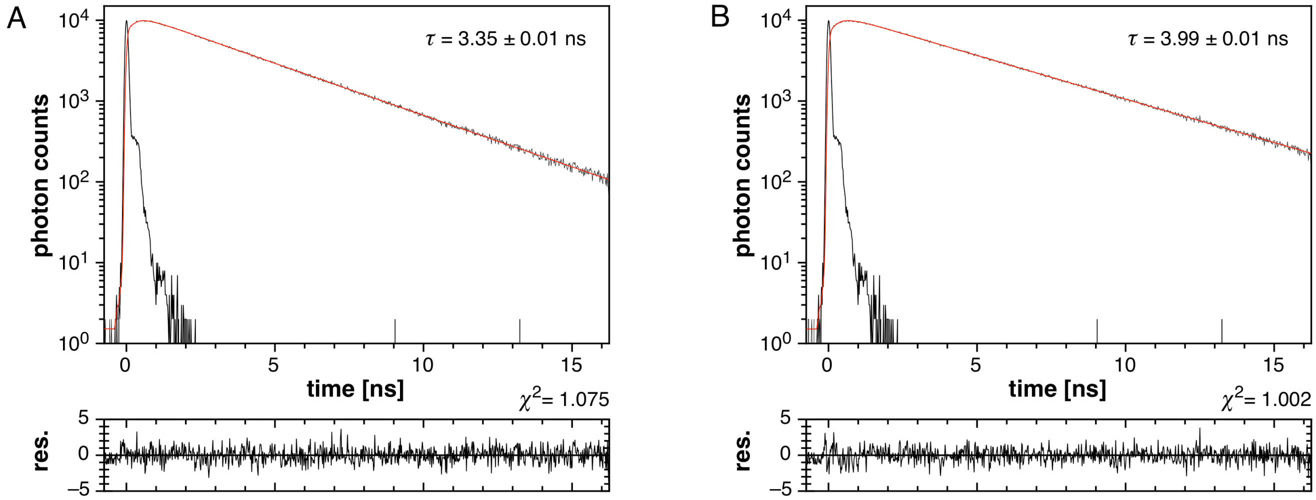

Figure S2: Fluorescence decay profile of compound (±)-**3** in 1 mM HCl (A) and 1 mM DCl (B) at 25°C.

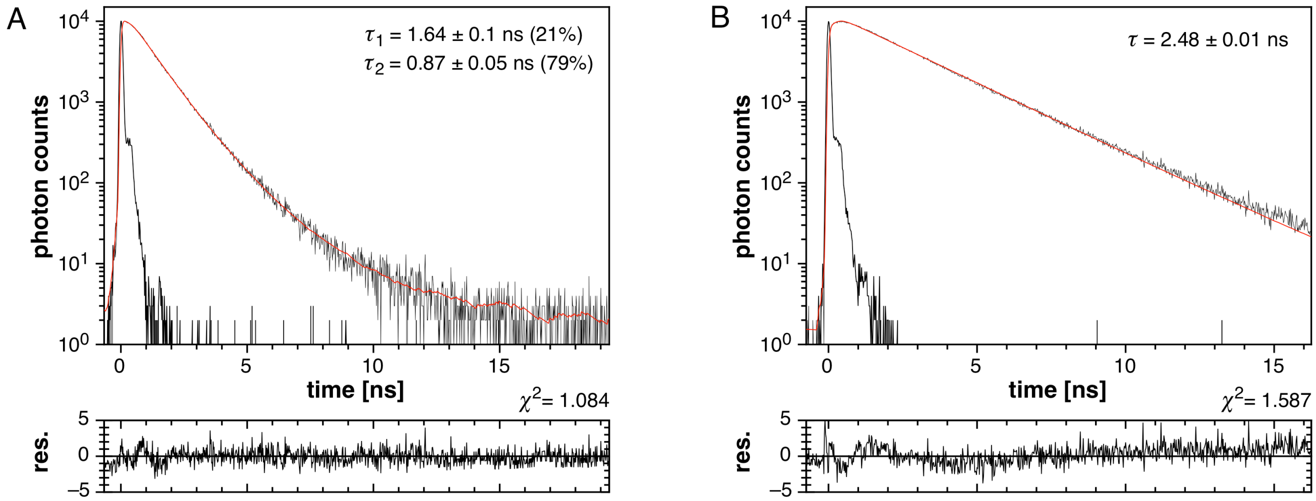

Figure S3: Fluorescence decay profile of Cu(I)-saturated pyrazoline derivative (±)-**5** (A) and CTAP-3 (B) in aqueous buffer (10 mM MOPS, pH 7.2) at 25°C.

Analyte Selectivity (CTAP-3). A 4 μM solution (50 mL) of CTAP-3 was prepared in 10 mM pH 7.0 PIPES-K^+ buffer containing 100 nM MCL-1. The fluorescence spectrum was recorded over the emission range of 380 to 650 nm with 365 nm excitation before and after saturation with Cu(I), generated in situ from $CuSO_4$ and 100 μM ascorbate (25°C). Each divalent cation tested (20 μM for transition metals, 10 mM for Ca(II) and Mg(II)) was added to a 3 mL aliquot of the probe solution, which was mixed thoroughly by inversion, and the fluorescence spectrum was recorded after a 1 minute equilibration period. Sodium ascorbate (100 μM) was added, followed by $CuSO_4$ (5 μM), and the emission spectrum recorded again. Emission spectra were integrated over the range of 445-465 nm, and the resulting intensities were divided by that of the Cu(I)-saturated probe. No divalent cations gave rise to emission outside of this range, and in all cases the fluorescence response to Cu(I) in the presence of the competing cation was within 3% of the normal value. Metal cations were supplied as aqueous stock solutions of the following salts: Mg(II), Ca(II), Co(II), and Ni(II) as nitrates, Hg(II) as $Hg(OAc)_2$, and Mn(II), Fe(II), Cu(II) and Zn(II) as sulfates. To avoid aerial oxidation, Fe(II) stock solution was prepared immediately before use.

Molar-ratio Titration of CTAP-3 with Cu(I). A magnetically stirred 4 μM solution of CTAP-3 in deoxygenated pH 6.0 MES-K^+ containing 100 nM MCL-1 and 100 μM sodium ascorbate was titrated with 0.4 μM aliquots of $CuSO_4$ from a 300 μM aqueous stock solution (25 °C). After a 30 second mixing period, the fluorescence spectrum at each titration point was recorded from 380 to 650 nm with 365 nm excitation.

Determination of the Detection Limit. A nitric acid washed quartz cuvette was filled with 3 mL of a filtered, deoxygenated buffer consisting of HPLC-grade water, ACS-grade ascorbic acid (200 μM), KOH (170 μM), and the pH-independent Cu(I) chelator MCL-3 (100 pM) as a sequestrant for background copper. The solution was magnetically stirred, and immediately prior to the start of the titration the lid was removed from the cuvette to allow CTAP-3 and copper stock solutions to be added via plastic Eppendorf tips rather than metal needles. The fluorimeter excitation and emission monochromator slits were opened to 10 nm bandpass, and a 370/36 nm sharp cutoff bandpass filter was placed in the excitation beam path to remove a trace of white light leakage. The excitation monochromator was centered at 355 nm and the emission collected at 455 nm with a digital integration time of 5 seconds and a total scan time of 30 seconds for each titration point. To begin the titration, CTAP-3 (2 nM) was added, and the fluorescence recorded after a 30 second mixing period. Copper was added in 15 pM aliquots from a 30 nM aqueous $CuSO_4$ stock solution, which in turn was prepared by serial dilution of the same stock solution used for the molar ratio titration. Following each addition, the fluorescence intensity was recorded after a mixing time of at least 30 seconds. After the final titration point, excess MCL-3 (1 μM) was added to confirm the reversibility of the fluorescence response.

Glass Electrode Calibration. The protonation constant of CTAP-3 was determined as concentration constant ($\log K_H$) based on potentiometric titrations using a combination glass electrode with double junction. The glass electrode was calibrated at 25°C (under argon atmosphere) by titration of a strong acid (5 mM HCl, prepared by dilution of a 0.1 M standardized solution, Aldrich) with a strong base (0.1 M KOH, standardized solution, Aldrich) in the presence of 0.1 M KCl as ionic background using a water-jacketed temperature controlled titration vessel (Metrohm). From the experimental emf data, the endpoint, electrode potential and slope were determined using Gran's method as implemented in the GLEE software package (pK_w = 13.78, 0.1 M KCl).[9] The calibration

procedure was performed prior to the potentiometric titrations, and the experimental electrode potential and slope were derived as the average of the data from three independent titrations.

Determination of the Protonation Constant of CTAP-3. A 5 μM solution of CTAP-3 in aqueous 0.1 M KCl was prepared and the solution pH was adjusted to 6.0. To remove interfering dust particles or fibers, the solution was passed through a 0.2 μm membrane filter. A combined potentiometric and fluorimetric titration was carried out in a quartz cuvette with 1 cm pathlength by addition of HCl to adjust the pH between 6.0 and 0.5 (constant temperature accessory set to 25°C). After addition of each aliquot acid, the solution was allowed to equilibrate, the potential was recorded (in mV), and the fluorescence intensity was measured at 455 nm with excitation at 365 nm. The data were analyzed by non-linear least-squares fitting using equation (1)

$$F = \frac{F_{\max} K_{a1}[\mathrm{H_3O^+}] + F_{\min} K_{a1} K_{a2}}{[\mathrm{H_3O^+}]^2 + [\mathrm{H_3O^+}] K_{a1} + K_{a1} K_{a2}} \qquad (1)$$

where F is the measured fluorescence intensity at $pH_c = -\log[H_3O^+]$, F_{max} and F_{min} are the limiting fluorescence intensities, and K_{a1} and K_{a2} are the first and second protonation constants, respectively.

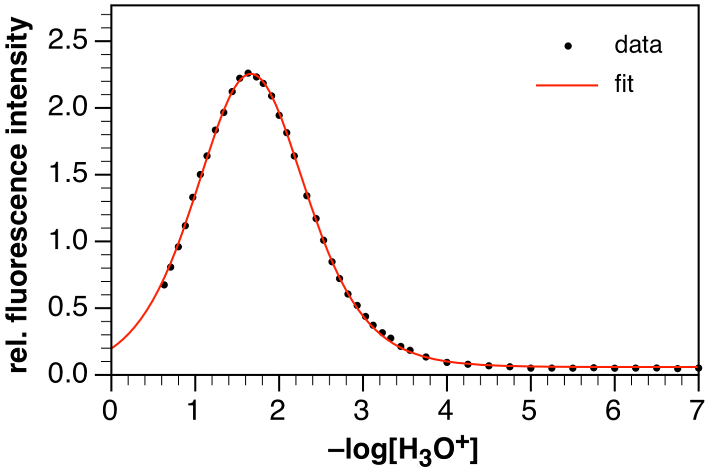

Figure S4: Fluorimetric pH titration of CTAP-3 at 25 °C in 0.1 M KCl. The fluorescence intensity at λ_{max} = 455 nm was recorded as a function of pH_c ($-\log[H_3O^+]$) and the data were analyzed by non-linear least squares fitting to yield the average pK_a values of pK_{H1} = 1.99 ± 0.01, and pK_{H2} = 1.32 ± 0.01.

Cu(I) Binding Affinity of CTAP-3. The Cu(I) stability constant for CTAP-3 was determined through a fluorimetric titration using the affinity standard MCL-2 as competing ligand.[6] A solution of $[(MCL\text{-}2)Cu]Na_3PF_6 \cdot 7.5\ H_2O$ (5 μM) and CTAP-3 (5 μM) in aqueous buffer (pH 6.0, 10 mM MES, 0.1 M $KClO_4$, 25°C) was equilibrated and then titrated with MCL-2 (3 mM stock solution in water) from 10-400 μM. After the addition of each aliquot, a fluorescence emission spectrum was acquired

with excitation at 365 nm over the range of 380-700 nm. The data were analyzed by non-linear least squares fitting using Specfit.[10] Based on the MCL-2 formation constant of logβ = 13.08 and a pK_a of 8.98 (corrected upward by 0.11 units to account for 0.1 M ionic strength),[11] an average log$K$ of 10.29 ± 0.06 was obtained.

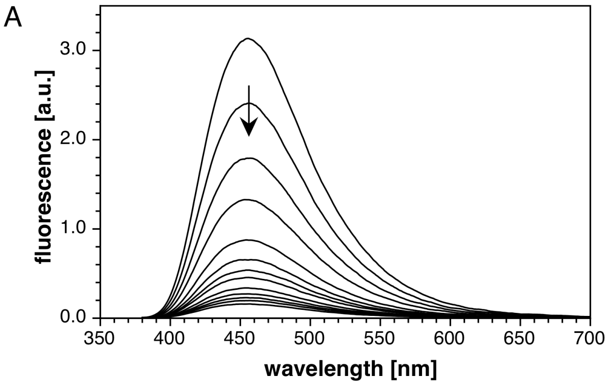

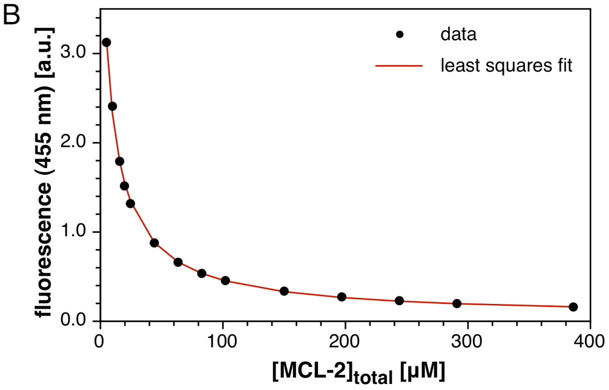

Equilibrium System Definition:

Species	Cu(I)	CTAP-3	MCL-2	H	logβ
Cu(I)	1	0	0	0	0.0
CTAP-3	0	1	0	0	0.0
MCL-2	0	0	1	0	0.0
(MCL-2)H	0	0	1	1	9.09
Cu(I)(MCL-2)	1	0	1	0	13.08
Cu(I)(CTAP-3)	1	1	0	0	*10.29 ± 0.06*

Figure S5: Fluorimetric determination of the Cu(I) stability constant of CTAP-3 (±)-**2** via competition with MCL-2. A solution of [(MCL-2)Cu]Na_3PF_6•7.5 H_2O (5 µM) and CTAP-3 (5 µM) was equilibrated in MES buffer at pH 6.0 (10 mM, 0.1 M $KClO_4$, 25°C) and then titrated with MCL-2 to a final concentration of 0.4 mM. The fluorescence traces (A) were analyzed by non-linear least squares fitting over the entire spectral range to yield an average log$K_{Cu(I)L}$ of 10.29 ± 0.06 (n = 3). Figure B shows the fluorescence intensity change and corresponding fit at 455 nm.

Preparation of Liposomes. A 2 mM solution of a 4:1 mixture of 1,2-dimyristoyl-*sn*-glycero-3-phosphocholine (DMPC) and 1,2-dimyristoyl-*sn*-glycero-3-phospho-(1'-rac-glycerol) (DMPG) was prepared according to the thin-film hydration method.[12] After dissolving DMPC (32.3 mg) and DMPG (8.21 mg) in 4:1 dichloromethane-methanol (20 mL), the solvent was evaporated under reduced pressure (Rotavap) to produce a thin film of the lipid. Remaining solvent traces were removed by drying under high vacuum for 2 hours. The lipid film was then hydrated by addition of 29.8 mL of buffer (10 mM PIPES, 0.1 M $KClO_4$, 100 nM MCL-1, pH 7.0) that had been deoxygenated by purging with argon. The resulting solution was sonicated for 5 min and passed 21 times through an extruder (LiposoFast, Avestin) equipped with a polycarbonate membrane (200 nm pore size). The freshly extruded DMPC/DMPG liposomes were immediately used for the experiments described below.

Liposome Size Distribution. Liposome diameters were measured based upon the Tunable Resistive Pulse Sensing (TRPS) principle using an Izon qNano particle analyzer (Izon Science Ltd., Burnside, New Zealand). The instrument was calibrated with monodisperse carboxylated polystyrene particles (Izon, 200 nm diameter) in deionized water following the manufacturer's recommendations. Calibration and particle analysis runs were conducted with a 46 mm nanopore stretch (NP150), an electric potential of 0.44 V, and an applied pressure of 10 mm Hg (variable pressure module). Each measurement was performed by detecting a minimum of 1000 particles. The data were analyzed using the Izon Control Suite 2.1 software to determine the mean diameter and size distribution of the liposomes.

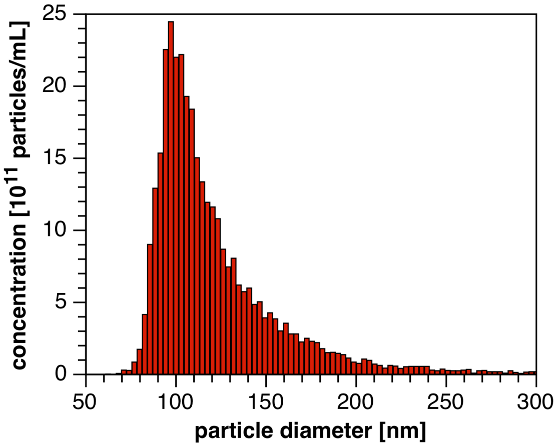

Figure S6: Size distribution of DMPC/DMPG (4:1) liposomes prepared in 10 mM PIPES buffer (pH 7.0, 0.1 M $KClO_4$, 25°C) through mechanical extrusion (200 nm pore size polycarbonate membrane). The average diameter of the liposomes is 120 nm and the size distribution peaks at a diameter of 100 nm.

Fluorescence Response of CTAP-2, CTAP-3, and CS3 towards Cu(I) in the Presence of DMPC/DMPG (4:1) Liposomes. The fluorescence response of CTAP-3 upon addition of Cu(I) (prepared by in situ reduction of $CuSO_4$) in the presence of 4:1 DMPC/DMPG liposomes was measured in PIPES buffer at pH 7.0 (10 mM, pH 7.0, 0.1 M $KClO_4$, 100 nM MCL-1). A freshly prepared 2 mM DMPC/DMPG (4:1) liposome stock solution in 10 mM PIPES (pH 7.0, 0.1 M $KClO_4$) was diluted into PIPES buffer and mixed by gentle inversion to yield a final liposome concentration of 100 μM. The resulting solution (3.0 mL) was transferred to a quartz cuvette (1 cm path length) and

CTAP-3 was added (300 μM stock solution in diH_2O) to a final concentration of 2 μM. The solution was supplemented with 250 μM sodium ascorbate as reducing agent, which was directly diluted into the cuvette from a 250 mM stock solution (diH_2O). The resulting solution was equilibrated for 30 min and $CuSO_4$ was added from a 6 mM stock solution (diH_2O) to yield final concentration of 3 μM. A fluorescence emission spectrum was acquired over the spectral range from 380-700 nm with excitation at 365 nm before and after addition of $CuSO_4$. The fluorescence response of CTAP-2 and CS3 towards Cu(I) was assessed under the same conditions described above for CTAP-3, except that CTAP-2 was diluted into the PIPES buffer from a 5 mM stock solution in diH_2O, and CS3 was diluted into the PIPES buffer from a 2 mM stock solution in DMSO. Fluorescence spectra for CTAP-2 were acquired over a spectral window of 400-700 nm with excitation at 380 nm, and fluorescence spectra for CS3 were acquired over a spectral window of 540-700 nm with excitation at 530 nm.

3. References

(1) Krutak, J. J.; Burpitt, R. D.; Moore, W. H.; Hyatt, J. A. *J. Org. Chem.* **1979**, *44*, 3847.
(2) Morgan, M. T.; Bagchi, P.; Fahrni, C. J. *J. Am. Chem. Soc.* **2011**, *133*, 15906.
(3) Guenadil, F.; Aichaoui, H.; Kapanda, C. N.; Lambert, D. M.; McCurdy, C. R.; Poupaert, J. H. *Monatsh. Chem.* **2011**, *142*, 67.
(4) DeOrazio, R. J.; Maeng, J.-H.; Manning, D. D.; Sherer, B. A.; Scott, I. L.; Nikam, S. S. *Synth. Commun.* **2011**, *41*, 3551.
(5) Lauria, A.; Abbate, I.; Gentile, C.; Angileri, F.; Martorana, A.; Almerico, A. M. *J. Med. Chem.* **2013**, *56*, 3424.
(6) Bagchi, P.; Morgan, M. T.; Bacsa, J.; Fahrni, C. J. *J. Am. Chem. Soc.* **2013**, *135*, 18549.
(7) Pardo, A.; Reyman, D.; Poyato, J. M. L.; Medina, F. *J. Luminesc.* **1992**, *51*, 269.
(8) Enderlein, J.; Erdmann, R. *Optics Commun.* **1997**, *134*, 371.
(9) Gans, P.; O'Sullivan, B. *Talanta* **2000**, *51*, 33.
(10) *SPECFIT Global Analysis System*, Spectrum Software Associates, Marlborough MA 01752, 2001
(11) Martell, A. E.; Smith, R. M. *Critical Stability Constants*; Plenum Press: New York, 1974.
(12) Bangham, A. D.; Standish, M. M.; Watkins, J. C. *J. Mol. Biol.* **1965**, *13*, 238.
